# Water, Sanitation, Hygiene, and Soil-Transmitted Helminth Infection: A Systematic Review and Meta-Analysis

**DOI:** 10.1371/journal.pmed.1001620

**Published:** 2014-03-25

**Authors:** Eric C. Strunz, David G. Addiss, Meredith E. Stocks, Stephanie Ogden, Jürg Utzinger, Matthew C. Freeman

**Affiliations:** 1 Children Without Worms, The Task Force for Global Health, Decatur, Georgia, United States of America; 2 Department of Environmental Health, Rollins School of Public Health, Emory University, Atlanta, Georgia, United States of America; 3 International Trachoma Initiative, The Task Force for Global Health, Decatur, Georgia, United States of America; 4 Department of Epidemiology and Public Health, Swiss Tropical and Public Health Institute, Basel, Switzerland; 5 University of Basel, Basel, Switzerland; University of Otago, New Zealand

## Abstract

**Background:**

Preventive chemotherapy represents a powerful but short-term control strategy for soil-transmitted helminthiasis. Since humans are often re-infected rapidly, long-term solutions require improvements in water, sanitation, and hygiene (WASH). The purpose of this study was to quantitatively summarize the relationship between WASH access or practices and soil-transmitted helminth (STH) infection.

**Methods and Findings:**

We conducted a systematic review and meta-analysis to examine the associations of improved WASH on infection with STH (*Ascaris lumbricoides*, *Trichuris trichiura*, hookworm [*Ancylostoma duodenale* and *Necator americanus*], and *Strongyloides stercoralis*). PubMed, Embase, Web of Science, and LILACS were searched from inception to October 28, 2013 with no language restrictions. Studies were eligible for inclusion if they provided an estimate for the effect of WASH access or practices on STH infection. We assessed the quality of published studies with the Grades of Recommendation, Assessment, Development and Evaluation (GRADE) approach. A total of 94 studies met our eligibility criteria; five were randomized controlled trials, whilst most others were cross-sectional studies. We used random-effects meta-analyses and analyzed only adjusted estimates to help account for heterogeneity and potential confounding respectively.

Use of treated water was associated with lower odds of STH infection (odds ratio [OR] 0.46, 95% CI 0.36–0.60). Piped water access was associated with lower odds of *A. lumbricoides* (OR 0.40, 95% CI 0.39–0.41) and *T. trichiura* infection (OR 0.57, 95% CI 0.45–0.72), but not any STH infection (OR 0.93, 95% CI 0.28–3.11). Access to sanitation was associated with decreased likelihood of infection with any STH (OR 0.66, 95% CI 0.57–0.76), *T. trichiura* (OR 0.61, 95% CI 0.50–0.74), and *A. lumbricoides* (OR 0.62, 95% CI 0.44–0.88), but not with hookworm infection (OR 0.80, 95% CI 0.61–1.06). Wearing shoes was associated with reduced odds of hookworm infection (OR 0.29, 95% CI 0.18–0.47) and infection with any STH (OR 0.30, 95% CI 0.11–0.83). Handwashing, both before eating (OR 0.38, 95% CI 0.26–0.55) and after defecating (OR 0.45, 95% CI 0.35–0.58), was associated with lower odds of *A. lumbricoides* infection. Soap use or availability was significantly associated with lower infection with any STH (OR 0.53, 95% CI 0.29–0.98), as was handwashing after defecation (OR 0.47, 95% CI 0.24–0.90).

Observational evidence constituted the majority of included literature, which limits any attempt to make causal inferences. Due to underlying heterogeneity across observational studies, the meta-analysis results reflect an average of many potentially distinct effects, not an average of one specific exposure-outcome relationship.

**Conclusions:**

WASH access and practices are generally associated with reduced odds of STH infection. Pooled estimates from all meta-analyses, except for two, indicated at least a 33% reduction in odds of infection associated with individual WASH practices or access. Although most WASH interventions for STH have focused on sanitation, access to water and hygiene also appear to significantly reduce odds of infection. Overall quality of evidence was low due to the preponderance of observational studies, though recent randomized controlled trials have further underscored the benefit of handwashing interventions. Limited use of the Joint Monitoring Program's standardized water and sanitation definitions in the literature restricted efforts to generalize across studies. While further research is warranted to determine the magnitude of benefit from WASH interventions for STH control, these results call for multi-sectoral, integrated intervention packages that are tailored to social-ecological contexts.

*Please see later in the article for the Editors' Summary*

## Abstract 2

In a systematic review and meta-analysis, Eric Strunz and colleagues examine whether improvements in water, sanitation, and hygiene (WASH) practices are associated with reduced risk of infections with soil-transmitted helminths.

*Please see later in the article for the Editors' Summary*

## Editors' Summary

**Background:**

Worldwide, more than a billion people are infected with soil-transmitted helminths (STHs), parasitic worms that live in the human intestine (gut). These intestinal worms, including roundworm, hookworm, and whipworm, mainly occur in tropical and subtropical regions and are most common in developing countries, where personal hygiene is poor, there is insufficient access to clean water, and sanitation (disposal of human feces and urine) is inadequate or absent. STHs colonize the human intestine and their eggs are shed in feces and enter the soil. Humans ingest the eggs, either by touching contaminated ground or eating unwashed fruit and vegetables grown in such soil. Hookworm may enter the body by burrowing through the skin, most commonly when bare-footed individuals walk on infected soil. Repeated infection with STHs leads to a heavy parasite infestation of the gut, causing chronic diarrhea, intestinal bleeding, and abdominal pain. In addition the parasites compete with their human host for nutrients, leading to malnutrition, anemia, and, in heavily infected children, stunting of physical growth and slowing of mental development.

**Why Was This Study Done?:**

While STH infections can be treated in the short-term with deworming medication, rapid re-infection is common, therefore a more comprehensive program of improved water, sanitation, and hygiene (WASH) is needed. WASH strategies include improvements in water access (e.g., water quality, water quantity, and distance to water), sanitation access (e.g., access to improved latrines, latrine maintenance, and fecal sludge management), and hygiene practices (e.g., handwashing before eating and/or after defecation, water treatment, soap use, wearing shoes, and water storage practices). WASH strategies have been shown to be effective for reducing rates of diarrhea and other neglected tropical diseases, such as trachoma; however, there is limited evidence linking specific WASH access or practices to STH infection rates. In this systematic review and meta-analysis, the researchers investigate whether WASH access or practices lower the risk of STH infections. A systematic review uses predefined criteria to identify all the research on a given topic; a meta-analysis is a statistical method that combines the results of several studies.

**What Did the Researchers Do and Find?:**

The researchers identified 94 studies that included measurements of the relationship between WASH access and practices with one or more types of STHs. Meta-analyses of the data from 35 of these studies indicated that overall people with access to WASH strategies or practices were about half as likely to be infected with any STH. Specifically, a lower odds of infection with any STH was observed for those people who use treated water (odd ratio [OR] of 0.46), have access to sanitation (OR of 0.66), wear shoes (OR of 0.30), and use soap or have soap availability (OR of 0.53) compared to those without access to these practices or strategies. In addition, infection with roundworm was less than half as likely in those who practiced handwashing both before eating and after defecating than those who did not practice handwashing (OR of 0.38 and 0.45, respectively).

**What Do These Findings Mean?:**

The studies included in this systematic review and meta-analysis have several shortcomings. For example, most were cross-sectional surveys—studies that examined the effect of WASH strategies on STH infections in a population at a single time point. Given this study design, people with access to WASH strategies may have shared other characteristics that were actually responsible for the observed reductions in the risk of STH infections. Consequently, the overall quality of the included studies was low and there was some evidence for publication bias (studies showing a positive association are more likely to be published than those that do not). Nevertheless, these findings confirm that WASH access and practices provide an effective control measure for STH. Controlling STHs in developing countries would have a huge positive impact on the physical and mental health of the population, especially children, therefore there should be more emphasis on expanding access to WASH as part of development guidelines and targets, in addition to short-term preventative chemotherapy currently used.

**Additional Information:**

Please access these websites via the online version of this summary at http://dx.doi.org/10.1371/journal.pmed.1001620.

- The US Centers for Disease Control and Prevention also provides detailed information on roundworm, whipworm, and hookworm infections
- The World Health Organization provides information on soil-transmitted helminths, including a description of the current control strategy
- Children Without Worms (CWW) partners with Johnson & Johnson, GlaxoSmithKline, the World Health Organization, national ministries of health and education, non-governmental organizations, and others to promote treatment and prevention of soil-transmitted helminthiasis. CWW advocates a four-pronged, comprehensive control strategy—Water, Sanitation, Hygiene Education, and Deworming (WASHED) to break the cycle of reinfection
- The Global Network for Neglected Tropical Diseases, an advocacy initiative dedicated to raising the awareness, political will, and funding necessary to control and eliminate the most common neglected tropical diseases, provides information on infections with roundworm (ascariasis), whipworm (trichuriasis), and hookworm
- WASH for the Neglected Tropical Diseases is a repository of information on WASH and the neglected tropical diseases (NTDs) such as soil-transmitted helminthiasis, and features a resource titled “WASH and the NTDs: A Manual for WASH Implementers.”
- Two international programs promoting water sanitation are the World Health Organization Water Sanitation and Health program and the World Health Organization/United Nations Childrens Fund Joint Monitoring Programme for Water Supply and Sanitation

## Introduction

More than a billion people are infected with soil-transmitted helminths (STHs) and many more live in high risk areas [1]. The global burden of STH infection is estimated at between 5 and 39 million disability-adjusted life years, largely attributable to anemia, stunting, and reduced cognitive development [2]–[4]. Humans are infected after ingesting eggs (*A. lumbricoides* and *T. trichiura*) or through penetration of the skin by infective larvae in the soil (hookworm [*A. duodenale* and *N. americanus*] and *S. stercoralis*) [1]. Current control strategies have focused on preventive chemotherapy through mass drug administration (MDA), in which at-risk populations are treated once or twice per year with benzimidazoles, primarily albendazole (usually given as a single oral dose of 400 mg) or mebendazole (500 mg) [5]. While preventive chemotherapy can greatly reduce morbidity from helminth infection, reinfection typically occurs rapidly after treatment [6].

Long-term STH control and eventual elimination require improvements to water, sanitation, and hygiene (WASH) access and practices [7]. The history of STH in the United States of America, South Korea, and Japan—where WASH improvements acted in concert with deworming to eliminate STH as a public health problem—supports the need for an integrated control paradigm [8]–[10]. WASH interventions are diverse, potentially including improvements in water access (e.g., water quality, water quantity, and distance to water), sanitation access (e.g., access to improved latrines, latrine maintenance, and fecal sludge management), and hygiene practices (e.g., handwashing before eating and/or after defecation, water treatment, soap use, wearing shoes, and water storage practices) [11]–[20]. Interventions often include multiple components, e.g., building ventilated-improved pit latrines while also providing hygiene education. Work in the WASH sector is often motivated by the view that access to clean water and adequate sanitation is a human right, but health outcomes are also broadly considered, with diarrheal disease burden representing a common measure of impact [21]–[23].

The successful integration of WASH into a disease control program has already been demonstrated for trachoma, which—like STH—is also considered a neglected tropical disease (NTD). The World Health Organization (WHO) endorses the “SAFE” strategy for trachoma control: surgery to correct advanced stages of trachoma, antibiotics to treat active infection, facial cleanliness to reduce disease transmission, and environmental change (including increased access to water and improved sanitation) [24]. The SAFE strategy explicitly calls for the implementation of improved access to, and use of, water, sanitation, and hygiene through improvements in delivery and/or specific interventions.

Such a fully integrated strategy—including guidelines and targets—does not yet exist for STH control, in part because evidence examining the relationship between WASH and STH is limited. A seminal review by Esrey and colleagues found few investigations that evaluated the association between WASH and STH infection [25]. A recent systematic review and meta-analysis by Ziegelbauer and colleagues found that individuals who have access to and use of sanitation facilities were at lower odds of infection with STH compared to individuals without sanitation [26]. Additional empirical evidence that links WASH improvements to reductions in STH infection is scarce, and an improved evidence-base may lead to better coordination between the NTD and WASH sectors [27],[28].

To fill this gap, we conducted a systematic review and set of meta-analyses to examine evidence of association between STH infection and WASH. We expanded the study's focus to include up-to-date meta-analyses for water and hygiene components, in addition to sanitation. We only used adjusted effect estimates in meta-analyses to help account for potential confounding and followed the Preferred Reporting Items for Systematic Reviews and Meta-Analyses (PRISMA) guidelines for systematic reviews. Our use of the Grades of Recommendation, Assessment, Development and Evaluation (GRADE) approach for quality assessment also provides a comprehensive accounting of the limitations of available evidence. We hypothesized that improvements in WASH would be associated with reductions in odds of STH infection. Thus, the purpose of this study was to quantitatively summarize the relationship between WASH access or practices on STH infection, while also synthesizing available data that did not qualify for meta-analysis.

## Methods

### Search Strategy, Inclusion Criteria, and Data Extraction

Our review adheres to the PRISMA and Meta-analysis of Observational Studies in Epidemiology (MOOSE) reporting guidelines (see Texts S1 and S2) [29]–[31]. The methods protocol is available in Text S3. A study investigator (ECS) and two research assistants (Rachel Stelmach [RS] and Claire Still [CS]) systematically searched PubMed, Embase, Web of Science, and LILACS for relevant articles from inception to October 28, 2013. We also indexed relevant studies from the bibliography of reviews by Ziegelbauer and colleagues [26] and Asaolu and Ofoezie [32]. Abstracts without published articles were considered eligible for inclusion. Additionally, we requested available unpublished research from the US Centers for Disease Control and Prevention, The Carter Center, The Task Force for Global Health, the WHO regional offices, and the authors' personal collections.

The native search engines within PubMed, Embase, Web of Knowledge, and LILACS were used to search each respective database using Boolean operators. The search included two clusters of terms: one for STH (i.e., helminth, soil-transmitted helminth, geohelminth, ascaris, lumbricoides, trichuris, trichiura, hookworm, ancylostoma, duodenale, necator, americanus, strongyloid*, stercoralis) and one for WASH (i.e., sanitation, sanitary engineering, water supply, waste management, environment*, excre*, faec*, fecal, feces, hand washing, handwashing, hygiene, latrine*, toilet*, water, soap). Results had to contain at least one term from both clusters. “Extensive search” was enabled when searching with Embase. Because Embase only allowed for exporting up to 5,000 records, results were stratified by date in order to screen and export all results in smaller segments. All search records were exported to bibliographic files and imported into Endnote X5 (Thomson Reuters), which was used to manage and screen search results. Titles, and when available, abstracts were scanned by an investigator (ECS) and also independently by research assistants (RS and CS) to determine possible relevance. Final selection was based on the full text of all potentially applicable articles. Ambiguous articles were examined by a senior reviewer (MCF).

Publications in all languages were considered. Studies in English, Spanish, Portuguese, and French were screened by investigators directly. Chinese-language articles were reviewed by a study collaborator (Shuyuan Huang [SH]) who assessed eligibility and extracted relevant data for the research team. Relevant data from all eligible studies was abstracted by a reviewer (ECS) and independently by assistants (RS and CS). Extracted data included study design, setting, year, population characteristics, WASH components measured, diagnostic approach, STH species, and relevant effect measures. Odds ratios (ORs) served as the primary effect measure in the reviewed literature. We collected both crude and adjusted estimates if available. Excel 2007 (Microsoft) was used to input and manage data using a long format to accommodate multiple effect estimates per study.

An article was eligible for inclusion if it presented a measure of effect between WASH and STH (e.g., an OR). For studies that pooled multiple intestinal parasites (e.g., *Giardia intestinalis* and STH) into one outcome measure, we contacted authors to request disaggregated data. We did not exclude studies based on methodology or population characteristics. Studies that evaluated multiple WASH components were included, as long as the components could be assessed separately from deworming medications and other non-WASH interventions.

There are few standard definitions for WASH access and practices, and it is difficult to measure WASH behaviors objectively [33]. We were unable to consistently connect water and sanitation variables reported in retrieved studies to the WHO and UNICEF Joint Monitoring Program's water and sanitation ladder definitions [34],[35]. For this review, “treated water” is defined as the use of any chemical or physical treatment of water to change its potability, whether conducted at the source or at the point of use. Two specific forms of treatment included boiling and filtering water at home. “Piped water” describes access to, or use of, water collected from a piped infrastructure, regardless of where the water is accessed (public/private) or how well maintained the infrastructure may be. “Sanitation access” was our primary sanitation exposure, defined as access to, or use of, any latrine. We did not exclude studies that lacked information about latrine quality, so access to sanitation could refer to anything from simple pit latrines to flush toilets. For hygiene, “washing after defecation” refers to the availability of handwashing resources (e.g., a wash basin) near sanitation facilities or reported handwashing behavior after defecation. “Soap use or availability” could refer to washing with water alone or no washing as the comparison group. Further, these definitions do not incorporate any criteria for compliance or consistency, since such details were rare in retrieved literature.

### Statistical Methods

We conducted meta-analyses for groups of effect estimates that related similar WASH access or practices (e.g., latrine availability and/or use became “sanitation access”) to a common outcome. Potential outcomes included infection with a specific STH (i.e., *A. lumbricoides*, *T. trichiura*, hookworm, and *S. stercoralis*) or any STH generally. Note that “any STH” reflected infection with an individual species or co-infection with multiple species when authors reported aggregated STH infection results. Meta-analyses were performed for groups of independent effect estimates that numbered three or greater and shared a similar exposure and infection outcome. A study that measured several WASH components could contribute to multiple meta-analyses, but could only supply one effect estimate for any single meta-analysis.

We employed random-effects models to account for the expected heterogeneity between studies [36]. Only adjusted estimates were utilized to limit the impact of confounding on pooled effect measures [37]. When necessary, we inverted estimates to reflect the effect of WASH, rather than the absence of WASH. This inversion was necessary in order to ensure enough study estimates were available for meta-analysis, but could have resulted in additional heterogeneity. For example, the inverse of “no sanitation access” may be similar to, but distinct from, “sanitation access” when assessed by questionnaire due to bias associated with socially desirable responses. Further, the presence of WASH access or practices may not necessarily be the same as the inverse effect of their absence, especially if important confounders or effect modifiers remain unexplored. Estimates of effect not included in meta-analyses were summarized in the text. The meta-analysis package MAIS for Stata version 12 (StataCorp) was used to perform the random-effects meta-analyses with the DerSimonian and Laird method [38]. The natural log of reported ORs was the dependent variable. CIs use the 95% level unless otherwise noted.

### Bias Assessment and Evidence Quality

We used the GRADE framework to assess potential sources of bias within studies and determine overall strength of evidence for each meta-analysis [39]. The GRADE approach is used to contextualize or justify intervention recommendations with four levels of evidence quality, ranging from very low to high. These levels correspond to how likely it would be for further research to alter conclusions drawn from the current evidence. “High quality” suggests that it is very unlikely for conclusions about effect estimates to change, whereas “very low quality” suggests that any estimate of effect is highly uncertain [40]. We formed our key bias categories from the literature, GRADE recommendations [41], and two instruments highlighted by the Cochrane Collaboration [42]: the Downs and Black tool [43] and the Newcastle-Ottawa scale [44]. We focused on five potential sources of bias in our assessment of individual studies: (i) diagnostic approach for assessing STH infection; (ii) exposure assessment; (iii) confounding assessment; (iv) response rate; and (v) selective reporting. Each study received one of three rankings for each source of bias: low risk, unclear risk, or high risk. Detailed criteria for these categories are available in Table 1. Bias was assessed independently by ECS and one of the two research assistants (RS and CS), compared, and reviewed by a senior assessor (DGA or MCF) if necessary.

**Table 1:** Criteria for study bias assessment.

Criteria	Description
**Infection diagnostics**	Is a diagnostic assay clearly mentioned? Is there any form of quality control in the diagnostic process (e.g., a senior technician doing spot-checks)?
**Exposure assessment**	Was exposure assessment (e.g., access to clean water, washing hands) ascertained via a self-reported survey response (unreliable) or observed directly by investigators (more reliable)? Is there any attempt to gauge proper use of water, hygiene, or some form of “quality control” for the exposures?
**Confounding assessment**	Are only crude estimates computed? Has matching and/or multiple logistic regression been undertaken to control for important potential confounders?
**Response rate**	Is the response rate (or loss-to-follow-up) similar for infected *versus* non-infected individuals?
**Selective reporting**	Is there evidence of selective reporting within an article (e.g., outlining certain variables of interest in the methods but not providing any data on them in the results)?

We assessed the overall quality of evidence for each meta-analysis after considering seven key characteristics. Each meta-analysis could receive a quality grade of very low, low, moderate, or high [45]. Meta-analyses of observational studies were classified as “low” by default, but could be downgraded (because of imprecision, indirectness, inconsistency, publication bias, and potential confounding) or upgraded (because of magnitude of effect, dose-response relationship, and potential confounding) on the basis of the overall strength of the evidence.

Inconsistency (i.e., heterogeneity) was assessed with Moran's *I* ^2^ and Cochran's *Q*-test [46]. *I* ^2^ provides an estimate of the proportion of variability in a meta-analysis that is explained by differences between the included studies instead of sampling error [47]. If a study exhibited an *I* ^2^ value over 50%, there was potential cause for concern, and the *Q*-test was also checked for a *p*-value less than 0.10. Values for *I* ^2^ over 70% or *Q*-test *p*-values lower than 0.05 resulted in the automatic downgrading of a body of evidence.

Publication bias was assessed through a visual inspection of funnel plots, though Egger's test also informed our interpretation [48]. Evidence quality was downgraded due to “imprecision” if the pooled effect estimate's 95% CI overlapped with the null (i.e., statistical significance at the 0.05 level). Although we provide CIs for pooled point estimates, imprecision remains a valuable criterion since not all consumers of reviews understand the importance of CIs and statistical uncertainty.

Evidence quality was upgraded owing to large magnitude of effect if the meta-analysis yielded a pooled OR less than 0.33 or greater than 3.0 [41]. Traditionally, risk ratios (RRs) are considered to show a large magnitude if they are less than 0.5 or greater than 2.0. However, ORs overstate the effect size compared to RRs, especially when initial risk (i.e., the prevalence of the outcome of interest) is high [49]. Because STH infection is relatively common, a more conservative threshold was needed for ORs in order to qualify as a large magnitude of effect.

Evidence quality could also be upgraded or downgraded on the basis of any unaccounted sources of potential confounding that would likely have a predictable direction on the effect estimate. For example, hygiene behaviors are typically over-reported in surveys, which could reduce the measured strength of effect for hygiene practices since the exposure group includes those who did not practice hygiene [50]–[52].

Due to the breadth of the review, indirectness was not a common concern, but would be more important for future reviews that focus on specific populations, settings, or interventions. Dose-response relationships were assessed by examining studies where exposures were discretized into ranked categories, e.g., analyzing “always washes hands” *versus* both “sometimes” and “never.” A dose-response relationship was considered possible if the point estimates improved between the ordinal categories, especially if relevant CIs did not overlap. Additional details about the meta-analysis GRADE criteria are available in Table 2.

**Table 2:** Criteria for meta-analysis GRADE assessment.

Criteria	Description
**Imprecision**	Caused the evidence quality to be downgraded if the pooled effect estimate's 95% CI overlapped with the null (i.e., one for odds ratios). In this context, imprecision is synonymous with a pooled estimate being statistically non-significant at the 0.05 level. Imprecision is used to downgrade evidence quality because some consumers of reviews (e.g., policymakers and practitioners) often do not fully understand statistical uncertainty.
**Indirectness**	Did not cause any evidence quality to be downgraded. Our review had a broad scope that aimed to collect a wide array of evidence exploring different populations and contexts. Traditionally, indirectness refers to issues that may limit the generalizability of evidence's reported results to the review's specified research question. This could be caused by differences in study population, study design, co-interventions, etc.
**Inconsistency** (i.e., heterogeneity)	Assessed with Moran's *I* ^2^ and Cochran's *Q*-test [46]. If a study exhibited an *I* ^2^ value over 50%, there was potential cause for concern, and the *Q*-test was also checked for a *p*-value less than 0.10. Values for *I* ^2^ over 70% or *Q*-test *p*-values lower than 0.05 resulted in the downgrading of a body of evidence.
**Publication bias**	Assessed through a visual inspection of funnel plots, though Egger's test also informed our interpretation [48]. Detecting publication bias is difficult when dealing with dichotomous outcomes, especially when there is significant between-study heterogeneity. In such circumstances, the popular Egger's test is usually inappropriate, with the potential to result in many false positives. For this reason, qualitative funnel plot analysis served as our primary assessment tool, though we also computed Egger's statistics to inform our judgment. Tests described by Rücker et al. [135] and Peters et al. [136] were also considered, but not performed.
A large **magnitude of effect** (also called “effect size”)	Could upgrade overall evidence quality if pooled odds ratios were less than 0.33 or greater than 3.0 [41]. The standard criteria for risk ratios and hazard ratios is that effect estimates be less than 0.5 or greater than 2.0. However, since odds ratios will show a greater magnitude than risk ratios, especially when an outcome is common, a more conservative cut-off value is needed. No firm rules have been established in the literature, so we increased the relevant effect size magnitude for odds ratios by 50%.
Evidence of a **dose-response relationship**	Can upgrade evidence quality. Dose-response relationships were assessed by examining studies where exposures were discretized into ranked categories, e.g., analyzing “always washes hands” *versus* both “sometimes” and “never.” A dose-response relationship was considered possible if the point estimates improved between the ordinal categories, especially if relevant confidence intervals did not overlap.
**Potential confounding**	Can upgrade a body of evidence if there are plausible factors that may be artificially weakening the observed pooled measurement. In the case of hygiene, individuals are known to overreport handwashing behaviors, which would systematically lower any apparent benefits. Potential downgrades are also possible, however, especially if established confounding variables are not taken into account by an analysis.

## Results

### Retrieved Studies

**Figure 1:**
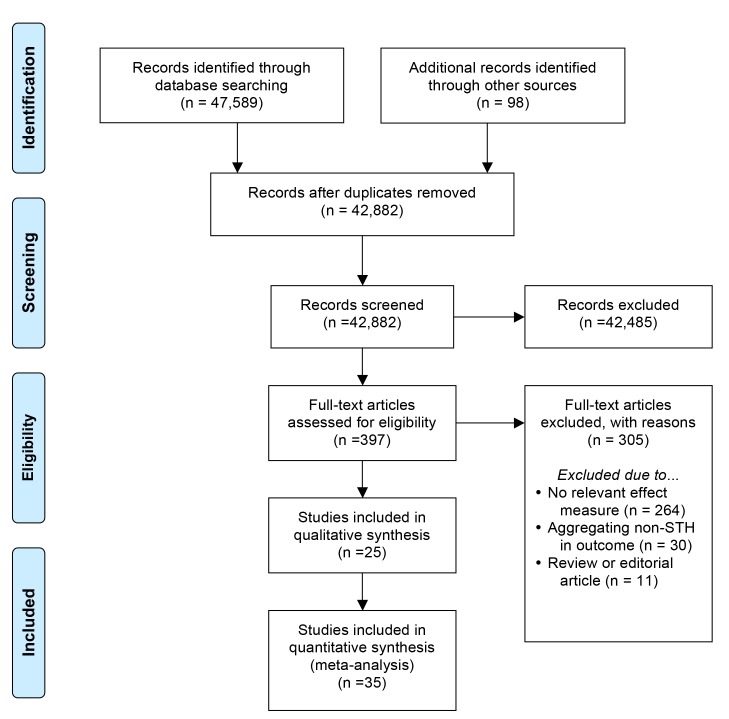
PRISMA flow diagram.

The search yielded a total of 47,589 articles from PubMed (*n* = 21,718), Embase (*n* = 18,188), Web of Knowledge (*n* = 7,502), and LILACS (*n* = 181), with 42,882 unique records. Our PRISMA flow diagram is available in Figure 1. After reviewing titles and abstracts, we examined 397 articles more intensively: 264 were excluded for lacking a relevant effect measure, 30 were excluded for aggregating non-STH infections in the outcome, and 11 were excluded for being review or editorial articles (see Tables 3–5 for included studies and S1 for excluded ones). We contacted 11 authors to obtain additional data [53]–[60], but only three authors responded [61]–[63]. A total of 94 studies ultimately met our inclusion criteria, yielding over 450 estimates of effect. Retrieved data included findings from one unpublished investigation [64] and one publication with information about two related studies [65].

**Table 3:** List of included studies with authors A–F.

Author [cite ID], Year - Country	Title of Article	Setting and Population	Sample Size	Diagnosis Method	Exposure Assessment and Study Method	Main WASH Components	Adjustment or Controlled Variables
Ahmed [111], 2011 - Malaysia^a^	The burden of moderate-to-heavy soil-transmitted helminth infections among rural malaysian aborigines: an urgent need for an integrated control programme	Satak, Raub district, Pahang-Sekolah Kebangsaan Satak school; Aboriginal schoolchildren, 6–13 years old	254	Kato-Katz and Harada Mori	Questionnaire, cross-sectional	Toilet, water source, playing in soil	Source of drinking water, toilet in house, domestic animals in the house, age, playing barefoot in soil
Aimpun [113], 2004 - Belize^a^	Survey for intestinal parasites in Belize, Central America	5 villages in the Toledo district; all ages, Ketchi and Mopan ethnic groups	533	Formalin-ethyl-acetate concentration technique	Questionnaire, cross-sectional	Handwashing, shoes, water, latrine	Race, occupation, years of education, population density, presence of trash pit near home, drinking water source, water treatment, and ownership of electrical appliances
Alemu [137], 2011 - Ethiopia	Soil transmitted helminths and schistosoma mansoni infections among school children in Zarima town, northwest Ethiopia	Elementary school children from Zarima town in NW Ethiopia	319	Kato-Katz	Questionnaire, observation, cross-sectional	Handwashing, shoe wearing, presence of latrine, latrine usage, water source	No adjusted WASH effect estimates identified
Al-Mekhlafi [138], 2007 - Malaysia	An unceasing problem: soil-transmitted helminthiases in rural malaysian communities	18 villages around Pos Betau School, Kuala Lipis; Primary schoolchildren (7–12) of Pos Betau School, Kuala Lipis, Pahang, Malaysia.	277	Kato-Katz and Harada Mori	Questionnaire, cross-sectional	Latrine availability, water access	No adjusted WASH effect estimates identified
Al-Mekhlafi [139], 2008 - Malaysia	Pattern and predictors of soil-transmitted helminth reinfection among aboriginal schoolchildren in rural Peninsular Malaysia	Pos Betau, Kuala Lipis, Pahang; Orang Asli (aborigine) primary schoolchildren, age 7–12	120	Modified cellophane thick smear and Harada Mori	Questionnaire, longitudinal	Toilet, water source	No adjusted WASH effect estimates identified
Alvarado [85], 2006 - Colombia	Social determinants, feeding practices and nutritional consequences of intestinal parasitism in children 7–18 months old in Guapi, Cauca	Guapi, Cauca; children 7–18 months old	136	Direct examination and concentrate Ritchie-Frick modified	Questionnaire, cross-sectional	Latrine type, floor type	No adjusted WASH effect estimates identified
Amahmid [140], 2005 - Morocco	Assessment of the health hazards associated with wastewater reuse: transmission of geohelminthic infections (Marrakech, Morocco)	Children (2–14 years) near Marrakech, Morocco	610	Formol-ether concentration	Questionnaire, observation, cross-sectional	Source of water	No adjusted WASH effect estimates identified
Asaolu [123], 2002 - Nigeria^a^	Effect of water supply and sanitation on the prevalence and intensity of Ascaris lumbricoides among pre-school-age children in Ajebandele and Ifewara, Osun State, Nigeria.	Ajebandele and Ifewara, two peri-urban communities near Ile-Ife, Osun State, Nigeria; children aged 0 to 108 months from mix of different ethnic groups	516	Kato-Katz (modified)	Questionnaire, cross-sectional	Latrine type, water source	Final, full model not given. Used stepwise selection in multiple regression. Initial model included: village, water source, latrine type, mothers' age and education, fathers' age and education, and gender/age of the child
Awasthi [141], 2008 - India	Prevalence and risk factors associated with worm infestation in pre-school children (6–23 months) in selected blocks of Uttar Pradesh and Jharkhand, India	Preschool children (6–23 months) from Uttar Pradesh and Jharkhand, India	909	Formol-ether concentration	Questionnaire, cross-sectional	Drinking water source, toilets in home, washing hands after defecation	No adjusted WASH effect estimates identified
Balen [131], 2011 - China^a^	Risk factors for helminth infections in a rural and a peri-urban setting of the Dongting Lake area, People's Republic of China	Wuyi and Laogang, two administrative villages in the Dongting Lake region of Hunan province; all ages from Wuyi, a rural village	1,298	Kato-Katz	Questionnaire, cross-sectional	Handwashing, water source	Village, occupation, socio-economic status, soil contact, animal ownership, washing hands w/soap before eating/after defecating
Barreto [142], 2010 - Brazil	Impact of a citywide sanitation program in Northeast Brazil on intestinal parasites infection in young children	Children (0–36 months) from Salvador, Brazil	1,920	Kato-Katz	Questionnaire, observation, cross-sectional	Regularity of water supply, hygiene behavior, indoor toilet, household excreta disposal	Different variables depending on model, but could include: drainage type, regularity of water supply, absence of rubbish dumps, paved road/sidewalk, hygiene behavior, indoor toilet, open sewage nearby, household excreta disposal, coverage with program sewerage connections
Basualdo [143], 2007 - Argentina	Intestinal parasitoses and environmental factors in a rural population of Argentina, 2002–2003	Children (<15 years) and adults (≥15 years) from Buenos Aires, Argentina	504	Telemann	Survey, cross-sectional	Type of floors, water supply, public/private faucet, excrement disposal	Final multivariable model unclear
Belo [144], 2005 - São Tomé and Príncipe	Prevalence, behavioural and social factors associated with Schistosoma intercalatum and geohelminth infections in Sao Tome and Principe	Three primary schools in S. Marya, Guadalupe and Kilombo; schoolchildren	130	Kato-Katz and Teleman-Lima	Questionnaire, cross-sectional	Excreta location	No adjusted WASH effect estimates identified
Belyhun [70], 2010 - Ethiopia^a^	Prevalence and risk factors for soil-transmitted helminth infection in mothers and their infants in Butajira, Ethiopia: a population based study	Butajira; infants	908	Formol-ether concentration method	Questionnaire, cross-sectional	Soap use, water source	Place of residence, age, domestic animals living together
Bieri [76], 2013 - China	Health-Education Package to Prevent Worm Infections in Chinese Schoolchildren	Rural Linxiang City District, Hunan province; children 9–10 years old	1,718	Kato-Katz with 10% quality control	Experimental, longitudinal	Handwashing	Clustering, school grade level, sex
Carneiro [145], 2002 - Brazil	The risk of Ascaris lumbricoides infection in children as an environmental health indicator to guide preventive activities in Caparao' and Alto Caparao', Brazil	Rural municipalities of Caparao and Alto Caparao, in Minas Gerais, Brazil; Children under 14 years of age	760	Kato-Katz	Questionnaire, cross-sectional	Sanitation index, hygiene index, water in washbasin	Crowding, water in washbasin, sanitation index, hygiene index, age, socioeconomic index
Chongsuvivatwong [65], 1996 - Thailand^a^	Predictors for the risk of hookworm infection: experience from endemic villages in southern Thailand	One village; All age groups (over 6 years old)	245	Kato-Katz	Questionnaire, observations, cross-sectional	Shoes, latrine availability	Education, income level, location in village, number of houses w/in 20 m, latrine, wearing shoes outside
Chongsuvivatwong [65], 1996 - Thailand^a^	Predictors for the risk of hookworm infection: experience from endemic villages in southern Thailand	Three villages; All age groups (over 6 years old)	456	Kato-Katz	Questionnaire, observations, cross-sectional	Shoes, latrine availability	Education, income level, location in village, number of houses w/in 20 m, latrine, wearing shoes outside
Corrales [124], 2006 - El Salvador^a^	Association between intestinal parasitic infections and type of sanitation system in rural El Salvador	Eight rural and semi-urban communities in the states of La Libertad and La Paz, El Salvador; Heads of households	127	Evergreen Scientific Fecal Parasite Concentrator kit	Questionnaire, cross-sectional	Latrine type	Household clustering, age, anthelmintic meds in past 3 months, having dirt floor, owning pigs
Cundill [67], 2011 - Brazil	Rates and intensity of re-infection with human helminths after treatment and the influence of individual, household, and environmental factors in a Brazilian community	Americaninhas, Minas Gerais State; Individuals aged over 5 years	642	Kato-Katz and formalin ether	Questionnaire, longitudinal	Water source, latrine	Parental education level, electricity access
Dumba [86], 2013 - Uganda	Design and implementation of participatory hygiene and sanitation transformation (PHAST) as a strategy to control soil-transmitted helminth infections in Luweero, Uganda	Children in 19 villages around Luweero, Uganda	558	Kato-Katz	Assignment, questionnaire, experimental	PHAST intervention (participatory hygiene/sanitation transformation)	Multivariable modeling used for one part of study, included maintenance condition of household, level of education
Ellis [146], 2007 - China	Familial aggregation of human susceptibility to co- and multiple helminth infections in a population from the Poyang Lake region, China	Five villages in Poyang Lake region, Jiangxi Province; Individuals aged over 5 years	3,682	Kato-Katz (duplicate)	Questionnaire, cross-sectional	Water contact	No adjusted WASH effect estimates identified
Ensink [147], 2005 - Pakistan	High risk of hookworm infection among wastewater farmers in Pakistan	Males involved in farming with wastewater or regular water or in textile work and their children (2–12 years) in Faisalabad, Pakistan	1,704	Formolin-ether concentration	Questionnaire, observation, cross-sectional	Type of water supply, toilet, wearing shoes	Toilet, house construction, type of water supply
Farook [148], 2002 - India	Intestinal Helminthic Infestations among Tribal Populations of Kottoor and Achankovil Areas in Kerala (India)	Kottoor and Acbankovil; All age groups	258	Formol-ether sedimentation technique	Questionnaire, cross-sectional	Proper handwashing	No adjusted WASH effect estimates identified
Ferreira [149], 2000 - Brazil	Secular trends in child intestinal parasitic diseases in S. Paulo city, Brazil (1984–1996)	Sao Paolo households; children (0–5 years old) in Sao Paulo	1,044	Sedimentation techniques, unstained and Lugol-stained	Questionnaire, Longitudinal	Improved sanitation	Age, year of survey, and maternal education (or, alternatively, per capita income), housing conditions, access to health services
Fonseca [119], 2010 - Brazil^a^	Prevalence and factors associated with geohelminth infections in children living in municipalities with low HDI in North and Northeast Brazil	Ten Brazilian municipalities with low human development indices (HDI); Children	2,523	Kato-Katz and Sedimentation	Questionnaire, cross-sectional	Improved water	Maternal education, family income, presence of garbage near home, household crowding, urban/rural, gender (varied depending on worm outcome)
Freeman [27], 2013 - Kenya	The impact of a school-based hygiene, water quality, and sanitation intervention on soil-transmitted helminth reinfection: a cluster-randomized trial	40 government primary schools in Nyanza Province; school-age children, 7–13 years old	3,120	Kato-Katz w/quality control	Experimental, longitudinal	Integrated WASH intervention	Clustering, baseline infection

a Studies contributed to a meta-analysis.

**Table 4:** List of included studies with authors G–M.

Author [cite ID], Year - Country	Title of Article	Setting and Population	Sample Size	Diagnosis Method	Exposure Assessment and Study Method	Main WASH Components	Adjustment or Controlled Variables
Geissler [150], 1998 - Kenya	Geophagy as a risk factor for geohelminth infections: A longitudinal study of Kenyan primary schoolchildren	Children (standards 5–6)	200	Kato-Katz	Questionnaire, verified, prospective cohort	Geophagy, having toilet at home	No adjusted WASH effect estimates identified
Glickman [151], 1999 - Guinea	Nematode intestinal parasites of children in rural Guinea, Africa: Prevalence and relationship to geophagia	Children (1–18 years) from rural Guinea, Africa	286	Direct smear and centrifugal flotation with sugar solution	Questionnaire, cross-sectional	Source of drinking water, sanitary facilities, geophagia	Age, sex
Gunawardena [130], 2004 - Sri Lanka^a^	Socio-economic and behavioural factors affecting the prevalence of Ascaris infection in a low-country tea plantation in Sri Lanka	Maliboda estate plantation (low country, <275 m above sea level); Tea plant workers, 2–50 years (median = 13 years)	176	Kato-Katz	Questionnaire, cross-sectional	Washing hands, boiling water	Full, final model not provided. Used step-wise variable selection in regression. The following variables were entered into the initial model: age, gender, living quarters, educational status and monthly income of each subject, availability of sanitary facilities, water supply source, use of boiled water, handwashing behavior, and cleanliness of each subject's house and immediate environment.
Gunawardena [152], 2005 - Sri Lanka	Effects of climatic, socio-economic and behavioural factors on the transmission of hookworm (*Necator americanus*) on two low-country plantations in Sri Lanka	The “low country” Maliboda and Ayr plantations; 2–74 years old	477	Kato-Katz	Questionnaire, observations, Longitudinal	Washing behavior, toilet	Occupation, level of education, toilet availability, usage, location, water source, use of footwear, playing with mud (if child), cleanliness of home environment
Gunawardena [153], 2011 - Sri Lanka	Soil-Transmitted Helminth Infections among Plantation Sector Schoolchildren in Sri Lanka: Prevalence after Ten Years of Preventive Chemotherapy	Nuwara Eliya, Badulla, Kegalle, Ratnapura, and Kandy. These five districts are centrally located in the southern half of Sri Lanka; School children (grade 4)	1,890	Kato-Katz	Questionnaire, cross-sectional	Better household sanitation, as reflected by a latrine score of 74 or more	Altitude, time since last school sanitary inspection, mother's education, latrine score, gender
Guo-Fei [154], 2011 - China	Analysis of influencing factors of Trichuris trichiura infection in demonstration plots of comprehensive control of parasitic diseases	Demonstration plots in multiple regions, including Anhui, Jiangxi, Hunan, Guangxi, Hainan, Sichuan, Guizhou, Yunnan; Unclear		Kato-Katz	Questionnaires, cross-sectional	Numerous	Agricultural activity, consumption of raw vegetables, previous anthelmintic treatment; could also have included sex, age, region, education level
Gyorkos [155], 2011 - Peru	Exploring determinants of hookworm infection in Peruvian schoolchildren using a gender analysis	Primary schools in Belen, Peru; Grade 5 children	927	Kato-Katz	Questionnaire, cross-sectional	Shoes, improved water	Dirty fingernails, presence of potable water at home, wearing shoes
Gyorkos [77], 2013 - Peru	Impact of Health Education on Soil-Transmitted Helminth Infections in Schoolchildren of the Peruvian Amazon: A Cluster-Randomized Controlled Trial	Grade 5 schoolchildren in Peruvian Amazon	1,089	Kato-Katz	Assignment, questionnaire, experimental	Hygiene education intervention	Clustering, age, sex, SES status, presence of running water in the home, baseline values of outcome measures (e.g., baseline STH values, baseline knowledge values), time of year of baseline visit, length of follow-up
Habbari [156], 2001 - Morocco	Geohelminthic infections associated with raw wastewater reuse for agricultural purposes in Beni-Mellal, Morocco	Students (7–14) attending primary school in Beni Mallal, Morocco	1,999	Formaldehyde-ether	Questionnaire, cross-sectional	Source of water, toilet at home, hand-washing	No adjusted WASH effect estimates identified
Hall [72], 1994 - Bangladesh	*Strongyloides stercoralis* in an urban slum community in Bangladesh: factors independently associated with infection	Urban slum in Dhaka; older than 1 year	880	Ether sedimentation technique	Questionnaire, longitudinal	Sanitation, water source, soil	No adjusted WASH effect estimates identified
Halpenny [157], 2013 - Panama	Regional, Household and Individual Factors that Influence Soil Transmitted Helminth Reinfection Dynamics in Preschool Children from Rural Indigenous Panama	The comarca Ngabe-Bugle, a semi-autonomous political region; children from 0–48 months of age	356	FLOTAC and Kato-Katz	Questionnaire, longitudinal	Sanitation	Clustering, other covariates depended on worm outcome, but could include household density, child HAZ score, maternal education
Henry [158], 1988 - St. Lucia	Reinfection with Ascaris lumbricoides after chemotherapy: a comparative study in three villages with varying sanitation	Children (0–36 months) from St. Lucia	219	Formol-ether concentration	Questionnaire, observation, prospective cohort	Having piped water, having a water-sealed toilet	No adjusted WASH effect estimates identified
Hidayah [122], 1997 - Malaysia^a^	Socio-environmental predictors of soil-transmitted helminthiasis in a rural community in Malaysia	Bachok; children	363	Formol-ether method	Questionnaire, cross-sectional	Hygiene, indiscriminate defecation	Age, location of household
Hohmann [80], 2001 - Lao PDR^a^	Relationship of intestinal parasites to the environment and to behavioral factors in children in the Bolikhamxay province of Lao PDR	Bolikhamxay province; children aged below 15 years	709	Kato-Katz	Questionnaire, cross-sectional	Washing hands	Mountainous region, age, material possessions, cleaning after defecation
Huat [159], 2012 - Malaysia	Prevalence and Risk Factors of Intestinal Helminth Infection Among Rural Malay Children	Beris Lalang, a rural Muslim community; children 7–9 years old	79	Saline wet mounting technique	Questionnaire, cross-sectional	Eating raw salad	BMI, mother's education level
Hughes [73], 2004 - Pacific Islands^a^	Environmental influences on helminthiasis and nutritional status among Pacific schoolchildren	27 primary schools in 13 Pacific Island countries; Primary school children, aged 5–12 years	1,996	Kato-Katz	Questionnaire, observations, cross-sectional	Water supply, soap available, sanitation facilities (many covariates)	All estimates age, sex, nutritional status and school/cluster.
Humphries [132], 2011 - Ghana^a^	Epidemiology of Hookworm Infection in Kintampo North Municipality, Ghana: Patterns of Malaria Coinfection, Anemia, and Albendazole Treatment Failure	Four communities in Kintampo North Municipality: Jato-Akuraa (JA), Cheranda (C), Kawampe (KA), and Gulumpe (GU); study results include only those >15 years old (adults)	126	Kato-Katz	Questionnaire, cross-sectional	Latrine use, shoes	Age, gender, and community.
Ivan [116], 2013 - Rwanda^a^	Helminthic infections rates and malaria in HIV-infected pregnant women on anti-retroviral therapy in Rwanda	HIV-positive pregnant women	980	Kato-Katz	Questionnaire, cross-sectional	Water source, shoe wearing, washing hands after defecation	ART, employment, handwashing, CD4 count
Jiraanankul [133], 2011 - Thailand^a^	Incidence and Risk Factors of Hookworm Infection in a Rural Community of Central Thailand	Tungsor Hongsa community, Chachoengsao Province, 228 km east of Bangkok, Thailand; all ages	585	Kato-Katz, water-ethyl acetate sedimentation technique	Questionnaire, longitudinal	Latrine use, shoes, washing hands	Age, raising cats or buffalo
Khieu [87], 2013 - Cambodia	Diagnosis, Treatment and Risk Factors of Strongyloides stercoralis in Schoolchildren in Cambodia	Semi-rural villages in Kandal province; Primary school children	458	Kato-Katz, KAP culture, and Baermann technique	Questionnaire, cross-sectional	Sanitation, handwashing, shoes	No adjusted WASH effect estimates identified
Knopp [125], 2011 - Zanzibar^a^	From morbidity control to transmission control: time to change tactics against helminths on Unguja Island, Zanzibar	Individuals on the island of Unguja	2,858	Kato-Katz, koga agar plate method (KAP), and Baermann technique (BM)	Questionnaire, interview, cross-sectional	Latrine at home, washing hands before eating, washing hands after defecation	Sex, age, and village
Kounnavong [68], 2011 - Lao PDR	Soil-transmitted helminth infections and risk factors in preschool children in southern rural Lao People's Democratic Republic	Three rural remote districts of Savannakhet Province in southern Lao PDR; Pre-school children aged 12–59 months	570	Kato-Katz	Questionnaire, cross-sectional	Latrine access, improved water access	No adjusted WASH effect estimates identified
Koura [160], 2011 - Benin	Prevalence and risk factors for soil-transmitted helminth infection in Beninese women during pregnancy	Pregnant women at two maternity wards	300	Kato-Katz	Questionnaire, cross-sectional	Wearing shoes	No adjusted WASH effect estimates identified
Lee [161], 2007 - Brunei	Hookworm infections in Singaporean soldiers after jungle training in Brunei Darussalam	Singaporean soldiers returning from jungle training in Brunei Darussalam	113	Fecal screens via microscopy	Questionnaire, interview, cross-sectional	Water supply source, crawling on ground/soil, shoe use	No adjusted WASH effect estimates identified
Luoba [162], 2005 - Kenya	Earth-eating and reinfection with intestinal helminths among pregnant and lactating women in western Kenya	Pregnant women in Nyanza Province	824	Kato-Katz	Interview, prospective cohort (longitudinal intervention)	Geophagy	No adjusted WASH effect estimates identified
Mahmud [127], 2013 - Ethiopia^a^	Risk factors for intestinal parasitosis, anaemia, and malnutrition among school children in Ethiopia	12 primary schools; School children aged 6–15	600	Kato-Katz and direct saline wetmount, formalin ethyl concentration technique	Questionnaire, observations, cross-sectional	Latrine, hygiene, water source	Age and sex
Matthys [71], 2007 - Côte d'Ivoire	Risk factors for Schistosoma mansoni and hookworm in urban farming communities in western Côte d'Ivoire	Six agricultural zones in the town of Man, western Côte d'Ivoire; Households	716	Kato-Katz	Questionnaire, cross-sectional	Water source, latrine use	Clustering, sex, age, education level, socioeconomic status, household crowding
Mihrshahi [128], 2009 - Vietnam^a^	The effectiveness of 4 monthly albendazole treatment in the reduction of soil-transmitted helminth infections in women of reproductive age in Viet Nam	Women of reproductive age in Yen Bai province	366	Kato-Katz	Questionnaire, cross-sectional	Sanitary latrine system, shoe use	Age, education status, work (inside/outside), number of children, meat consumption, shoe use, latrine type, socio-economic status, and handwashing
Moraes [163], 2004 - Brazil	Impact of drainage and sewerage on intestinal nematode infections in poor urban areas in Salvador, Brazil	Nine poor urban areas of the city of Salvador (pop. 2.44 million), capital of Bahia State, in Northeast Brazil; children aged between 5 and 14 years old	1,893	Kato-Katz	Questionnaire, cross-sectional	Sanitation	Child's sex, child's age, number of children aged 5–14 years in the household, crowding (number of people per room), years of schooling of the household head, monthly per capita income, religion, animals in the house, and the house floor material
Moraes [164], 2007 - Brazil	[Household solid waste bagging and collection and their health implications for children living in outlying urban settlements in Salvador, Bahia State, Brazil].	Nine peri-urban settlements of the city of Salva-pain, Bahia, Brazil; Children 5–14 years old	1,893	Kato-Katz	Questionnaire, longitudinal	Solid waste collection	Age and sex of the child, number of household members, number of persons/room, monthly family income per capita, religion, presence of lavatory, floor of the home, and excreta disposal of sewage
Morales-Espinoza [117], 2003 - Mexico^a^	Intestinal parasites in children, in highly deprived areas of the border region of Chiapas, Mexico	Chiapas, 32 communities; children under 15 years of age	1,148	Faust Method	Questionnaire, cross-sectional	Water source, latrine	Age, overcrowding, living conditions, and educational level

a Studies contributed to a meta-analysis.

**Table 5:** List of included studies with authors N–Z.

Author [cite ID], Year - Country	Title of Article	Setting and Population	Sample Size	Diagnosis Method	Exposure Assessment and Study Method	Main WASH Components	Adjustment or Controlled Variables
Narain [84], 2000 - India^a^	Prevalence of Trichuris trichiura in relation to socio-economic and behavioral determinants of exposure to infection in rural Assam	Dibrugarh district in upper Assam; adults and children aged <15 years	580	Formol-ether concentration technique	Questionnaire, cross-sectional	Floor material, improved latrine, improved water	Age, open defecation, type of flooring, family size, number of children in household
Nasr [66], 2013 - Malaysia^a^	Towards an effective control programme of soil-transmitted helminth infections among Orang Asli in rural Malaysia. Part 1: Prevalence and associated key factors	13 villages in Lipis district, Pahang; Orang Asli children aged ≤15 years	484	Formalin-ether sedimentation, Kato Katz, and Harada Mori	Questionnaire, cross-sectional	Handwashing, water, sanitation	Age, family size, other WASH practices
Nguyen [165], 2006 - Vietnam	Intestinal helminth infections among reproductive age women in Vietnam: prevalence, co-infection and risk factors	53 provinces; reproductive-age women	5,127	Kato-Katz	Questionnaire, cross-sectional	Latrine, manure fertilizer use	Adjusted for infection with *A. lumbricoides*, *T. trichiura*, and interaction term between them.
Nishiura [81], 2002 - Pakistan	Ascaris lumbricoides among children in rural communities in the Northern Area, Pakistan: prevalence, intensity, and associated socio-cultural and behavioral risk factors	Five rural villages in the northern area of Pakistan; school children	492	Kato-Katz	Questionnaire, cross-sectional	Washing hands, latrine, eating soil, soap	Age, sex, living with child under age of 5, other WASH practices
Norhayati [166], 1999 - Malaysia	Some risk factors of Ascaris and Trichuris infection in Malaysian aborigine (Orang Asli) children	Children ages 1–13	205	Kato-Katz and Harada Mori	Questionnaire, cross-sectional	Usage of well-water, usage of toilets	No adjusted WASH effect estimates identified
Nwaneri [83], 2012 - Nigeria	Intestinal helminthiasis in children with chronic neurological disorders in Benin City, Nigeria: intensity and behavioral risk factors	Benin City child neurology clinic; Children with chronic neurological disorders	155	Kato-Katz	Questionnaire, case-control with matching on age/sex	Hygiene practices	Age, sex
Olsen [167], 2001 - Kenya	A study of risk factors for intestinal helminth infections using epidemiological and anthropological approaches	Villages in Kisumu District, Nyanza Province, Kenya; All inhabitants over the age of 4 years	333	Kato-Katz (duplicate)	Questionnaire, cross-sectional	Latrine, soap	Adjusted for crowding in households, children under five years of age, soap use, latrine presence.
Ortiz Valencia [168], 2005 - Brazil	Spatial ascariasis risk estimation using socioeconomic variables.	Children ages 1–9	1,550	Unclear	Interview, cross-sectional	Water filtration	No adjusted WASH effect estimates identified
Parajuli [129], 2009 - Nepal^a^	Behavioral and Nutritional Factors and Geohelminth Infection Among Two Ethnic Groups in the Terai Region, Nepal	Parsauni village in the Sakhawaparsauni Village Development Committee (VDC) of Parsa district, Nepal; Mushar and Tharu (ethnic groups) inhabitants, aged 20–60 years	95	Direct wetmount Lugol's iodine thin-smear method	Questionnaire, cross-sectional	Soap, walking barefoot	Adjusts for age, ethnicity, gender, height.
Pham-Duc [115], 2013 - Vietnam^a^	Ascaris lumbricoides and Trichuris trichiura infections associated with wastewater and human excreta use in agriculture in Vietnam	Nhat Tan and Hoang Tay communes in Kim Bang district, Hanam province; Individuals over 1 year old	1,425	Kato-Katz thick smear and formalin-ether concentration techniques	Questionnaire, cross-sectional	Water, sanitation, handwashing	Age, sex, and season.
Phiri [134], 2000 - Malawi^a^	Urban/rural differences in prevalence and risk factors for intestinal helminth infection in southern Malawi	Two sites in the Blantyre area of Malawi: Ndirande a densely populated, poor, urban township in Blantyre city; and Namitambo, a poor rural community in Chiradzulu district; children between the age of 3–14 years	273	Stoll's egg count technique	Questionnaire, cross-sectional	Sewage, walking barefoot	Age, sex, mother's education, school attendance, sewage around house
Quintero [69], 2012 - Venezuela	Household social determinants of ascariasis and trichuriasis in North Central Venezuela	55 municipalities of the North Central Venezuela states Aragua, Carabobo, Miranda, Vargas and Capital District; Children and adults (3 months–60 years old)	3,388; ∼4.7 million with weights	Kato-Katz	Questionnaire, cross-sectional	Improved water, soil floor, sewage disposal	Rural/urban, house vulnerability, waste disposal practices
Riess [169], 2013 - Tanzania	Hookworm Infection and Environmental Factors in Mbeya Region, Tanzania: A Cross-Sectional, Population-Based Study	Participants from nine different sites in Mbeya region, south-western Tanzania	6,375	Kato-Katz	Questionnaire	Latrine coverage, latrine type	Age, previous anthelmintic treatment, clustering
Rísquez [170], 2010 - Venezuela	Condiciones higiénico-sanitarias como factores de riesgo para las parasitosis intestinales en una comunidad rural venezolana	Students in the Panaquire-Miranda school district	69	Formol-ether concentration	Questionnaire	Defecation practices	No adjusted WASH effect estimates identified
Roy [114], 2011 - Bangladesh^a^	Patterns and risk factors for helminthiasis in rural children aged under 2 in Bangladesh	10 villages in Rural Mirzapur; Rural children under 2 years old	252	Formalin-ether sedimentation technique	Questionnaire, longitudinal	Improved water, excreta disposal	Adjusted by age, sex, breastfeeding, seasonality, and disposal site of child feces
Saathoff [171], 2002 - South Africa	Geophagy and its association with geohelminth infection in rural schoolchildren from northern KwaZulu-Natal, South Africa	Pupils in third grade (average age of 10.7 years)	1,161	Kato-Katz	Interview, cross-sectional	Geophagy	Family
Schmidlin [126], 2013 - Côte d'Ivoire^a^	Effects of hygiene and defecation behavior on helminths and intestinal protozoa infections in Taabo, Côte d'Ivoire	People in villages/hamlets in south-central that were small populations and similar pop. structure	1,894	Kato-Katz	Questionnaire, interview, cross-sectional	Sanitation behavior, hygiene behavior	Socioeconomic status, age group, and sex
Scolari [172], 2000 - Brazil	Prevalence and distribution of soil-transmitted helminth (STH) infections in urban and indigenous schoolchildren in Ortigueira, State of Parana, Brasil: implications for control	School children ages 5–15	236	Kato-Katz	Questionnaires (verified by local field assistant), cross-sectional	Toilet ownership, location of toilet, safe water access	No adjusted WASH effect estimates identified
Sherkhonov [173], 2013 - Tajikistan	National intestinal helminth survey among schoolchildren in Tajikistan: Prevalences, risk factors and perceptions	Schools from across country; school children, 7–11 years old	1,642	Kato-Katz	Questionnaire, cross-sectional	Water, sanitation, handwashing	Clustering, other final covariates unclear
Soares Magalhaes [174], 2011 - Ghana, Mali, and Burkina Faso	Geographical analysis of the role of water supply and sanitation in the risk of helminth infections of children in West Africa	West African children	18,812	Kato-Katz	Questionnaire (health survey), cross-sectional	Water source, toilet, floor material	No adjusted WASH effect estimates identified
Steenhard [175], 2009 - Guinea-Bissau	Concurrent infections and socioeconomic determinants of geohelminth infection: a community study of schoolchildren in periurban Guinea-Bissau	Poor semirural area (Bandim II and Belem, near Bissau); school children aged 4–12	706	McMaster technique, formol-ether technique	Questionnaire, cross-sectional	Improved water, improved sanitation	No adjusted WASH effect estimates identified
Steinmann [79], 2010 - Kyrgyzstan^a^	Rapid appraisal of human intestinal helminth infections among schoolchildren in Osh oblast, Kyrgyzstan	Osh oblast; school children (grades 2 or 3, age: 6–15 years)	1,262	Kato-Katz	Questionnaire, cross-sectional	Washing vegetables, water source, toilet use	Age, sex, ethnic group, washing vegetables before eating, clustering
Stothard [120], 2008 - Zanzibar^a^	Soiltransmitted helminthiasis among mothers and their preschool children on Unguja Island, Zanzibar with emphasis upon ascariasis	10 Ungujan villages; mothers and their pre-SAC, 322 mothers, 359 children	681	Kato-Katz	Questionnaire, cross-sectional	Latrine access, wearing shoes, playing on ground	Clustering, having infected household member
Teixeira [176], 2004 - Brazil	Environmental factors related to intestinal helminth infections in subnormal settled areas, Juiz de Fora, MG	Children (1–5 years old) in the subnormal settlement areas in the municipality of Juiz de Fora, Mina Gerais.	753	Hoffmann-Pons-Janer method	Questionnaire	Water quality complaints, feces disposal	Family income, age of child
Trang [121], 2007 - Vietnam^a^	Helminth infections among people using wastewater and human excreta in peri-urban agriculture and aquaculture in Hanoi, Vietnam	Yen So commune (population 10,500 at the time of study), a rural area located about 10 km south of central Hanoi; adults of 15–70 years of age engaged in agricultural activities and preschool children (less than 72 months of age)	807	Direct smear method	Questionnaire, cross-sectional	Water source, latrine	Age, sex, socioeconomic status, other WASH practices
Trang [177], 2006 - Vietnam	Low risk for helminth infection in wastewater-fed rice cultivation in Vietnam	All females and males from 15–94 years old from 2 communes using different irrigation for rice cultivation (wastewater and river water)	1,139	Direct smear method	Questionnaire, interview, cross-sectional	Latrine availability, latrine status, handwashing (soap), availability of drinking water	Clustering, age, gender, excreta agricultural use
Traub [118], 2004 - India^a^	The prevalence, intensities and risk factors associated with geohelminth infection in tea-growing communities of Assam, India	Three tea-growing communities in Assam, India; tea-growing communities of rural Assam (no age restrictions)	328	Kato-Katz	Questionnaire, cross-sectional	Shoes, water source, latrine use	Socioeconomic status, age, household crowding, level of education, religion, use of footwear when outdoors, defecation practices, pig ownership, water source
Ugbomoiko [178], 2009 - Nigeria	Socio-environmental factors and ascariasis infection among school-aged children in Ilobu, Osun State, Nigeria	Small rural village of Ilobu in Irepodu Local Government Area of Osun State, Nigeria; children below 16 years of age	440	Kato-Katz	Questionnaire, cross-sectional	Water source, latrine, distance to waste disposal	Sex, age, which parent reside with child, number of playmates <6 or >5 years old, period of residency, and previous treatment status.
Walker [179], 2011 - Bangladesh	Individual Predisposition, Household Clustering and Risk Factors for Human Infection with Ascaris lumbricoides: New Epidemiological Insights	Dhaka; households	2,929	Ether sedimentation technique	Questionnaire, longitudinal	Shared latrines, shared water sources, floor material	Clustering, age, sex, household socioeconomic status, ethnicity, and household characteristics
Wang [112], 2012 - China^a^	Soil-Transmitted Helminth Infections and Correlated Risk Factors in Preschool and School-Aged Children in Rural Southwest China	141 impoverished rural areas of Guizhou and Sichuan Provinces in Southwest China; SAC and Pre-sac (3–5-year-old group and an 8–10-year-old group)	1,707	Kato-Katz	Questionnaire, cross-sectional	Washing hands, boiling water, latrine type, use of manure fertilizer	STH treatment history, individual characteristics, health and sanitation behaviors, and household characteristics
Wordemann [97], 2006 - Cuba^a^	Prevalence and risk factors of intestinal parasites in Cuban children	San Juan y Martinez and Fomento; Cuban schoolchildren aged 4–14	1,320	Kato-Katz	Questionnaire, cross-sectional	Water source, latrine use	Age, sex, municipality, urban/rural background, and interaction between municipality and urban/rural background
Worrell [74], 2013 - Kenya	Water, Sanitation, and Hygiene-Related Risk Factors for Soil-Transmitted Helminth Infection in Urban School- and Pre-School-Aged Children in Kibera, Nairobi	Kibera; pre-school and school-aged children	676	Kato-Katz (three stools)	Questionnaire, observations, cross-sectional	Numerous	Age, presence of an infected sibling(s) in the household, household crowding, deworming in the last year, ability to meet water needs, treating water, and soap use
Xu [75], 2001 - China	On cleanliness of hands in diminution of Ascaris lumbricoides infection in children	Shaowu, Fujian Province; Children (pupils in preliminary school)	654	Kato-Katz	Experimental, longitudinal	Handwashing	No adjusted WASH effect estimates identified
Yajima [180], 2009 - Vietnam	High latrine coverage is not reducing the prevalence of soil-transmitted helminthiasis in Hoa Binh province, Vietnam	Residents of Tien Xuan commune, Hoa Binh province, Vietnam	155	Kato-Katz	Questionnaire, cross-sectional	Latrine at home	No adjusted WASH effect estimates identified
Yori [88], 2006 - Peru	Seroepidemiology of strongyloidiasis in the Peruvian Amazon	Residents of Santo Tomas, Peru	908	Direct smear, Baermann, simple sedimentation agar plate, serologic assays (ELISA)	Questionnaire, cross-sectional	Source and storage of drinking water, human waste disposal, wearing of shoes	Age
Young [82], 2007 - Tanzania^a^	Association of geophagia with Ascaris, Trichuris and hookworm transmission in Zanzibar, Tanzania	Pemba Island, Zanzibar; pregnant women	970	Kato-Katz	Questionnaire, cross-sectional	Geophagy, improved sanitation	Geophagia during current pregnancy, age, urban/rural, number of durable goods, pit toilet in HH, formal education

a Studies contributed to a meta-analysis.

HAZ, height for age Z score; SES, socioeconomic status.

**Figure 2:**
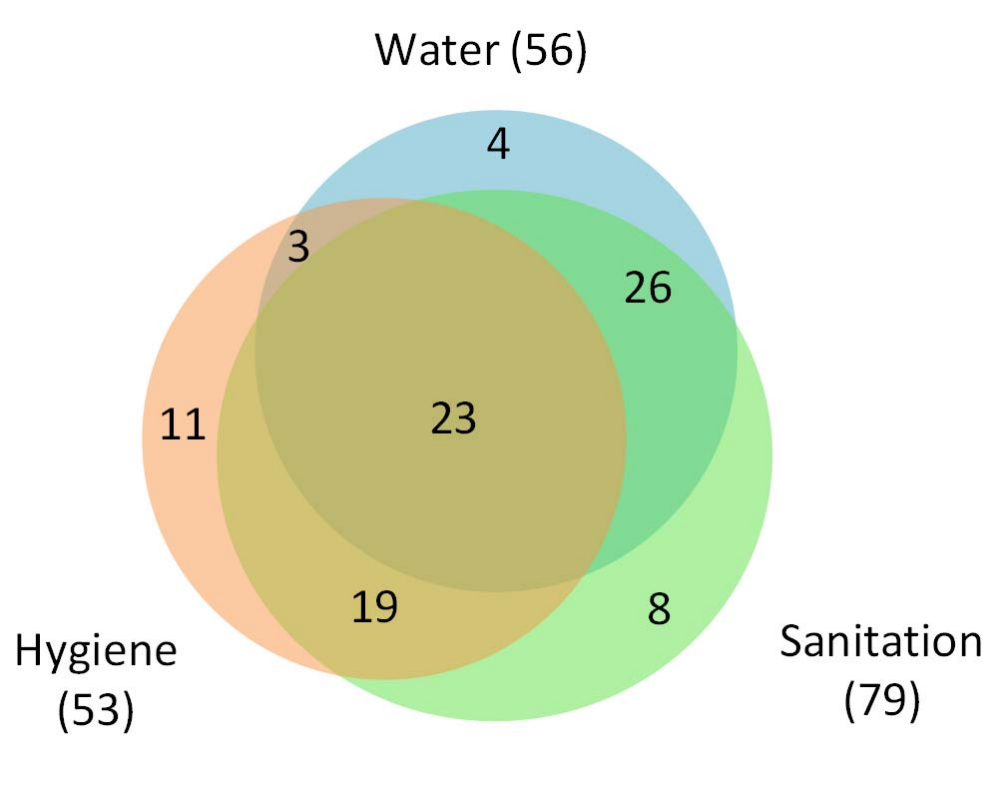
Retrieved articles by WASH group.

Most included studies were published in English (*n* = 86), though articles in Portuguese (*n* = 4), Chinese (*n* = 2), and Spanish (*n* = 2) were also included. Studies researched populations in Asia (*n* = 42), Africa (*n* = 29), and the Americas (*n* = 23). Studies investigated access and practices relating to water (*n* = 56), sanitation (*n* = 79), and hygiene (*n* = 53) (Figure 2); the most commonly explored were access to sanitation (*n* = 63), access to water (*n* = 45), handwashing (*n* = 30), and wearing shoes (*n* = 27). Studies reported investigating infection with *A. lumbricoides* (*n* = 69), *T. trichiura* (*n* = 60), hookworm (*n* = 63), *S. stercoralis* (*n* = 12), and any STH collectively (*n* = 52). Tables 6 and 7 illustrate the number of articles in which both specific WASH components and helminth infections were investigated.

**Table 6:** Number of studies (*n* = 94) that investigated STH species and WASH domains.

Studies	Water	Sanitation	Hygiene	Water and Sanitation	Water and Hygiene	Sanitation and Hygiene	Water, Sanitation, and Hygiene
Any STH (grouped)	34	44	28	32	16	21	15
*A. lumbricoides*	43	59	37	38	20	30	18
Hookworm	34	53	37	28	17	30	14
*T. trichiura*	38	52	34	34	20	28	18
*S. stercoralis*	10	11	6	9	4	5	3
Total (all studies)	56	79	53	49	26	42	23

Each cell indicates the number of reviewed studies that investigated both an STH species (or any STH) and WASH domains. Higher numbers suggest that certain WASH-STH relationships are more commonly explored in the literature.

**Table 7:** Number of studies that investigated STH species and WASH access and practices.

STH Species	Water			Sanitation				Hygiene					
	Water Access	Water Types^a^	Treat Water	Sanit. Access	Latrine Types^a^	Sharing Latrines	Latrine Maint.	Washing Hands	Soap	Washing Vegetables	Shoe Use	Geophagy	Hygiene Education
Any STH	30^b^	5	9^b^	34^b^	8	3	2	17^b^	7^b^	2	13^b^	4	4
*A. lumbricoides*	33^b^	3	15	45^b^	13	5	2	20^b^	9	2	14	8	4
Hookworm	28	2	11	44^b^	11	3	2	16	5	1	20^b^	8	2
*T. trichiura*	31^b^	3	12	41^b^	12	3	2	18	7	2	12	7	3
*S. stercoralis*	8	1	5	11	2	1	0	5	2	0	3	1	0

Cells with high numbers but no meta-analysis (no footnote) indicate that effect measures were not reported (selective reporting), reported measures were not statistically adjusted, or that the WASH access and practice was too diverse to be effectively grouped in a meta-analysis (e.g., handwashing can be measured before eating or after defecating).

a Water Types and Latrine Types refer to studies that measured multiple sanitation comparisons, not just “latrine versus no latrine.” For example, a study could examine water collected from rivers, wells, or piped connections.

b Gray cells indicate that a meta-analysis was conducted for that WASH variable and STH outcome.

Of 94 studies, 89 were observational: 75 used a cross-sectional epidemiologic design, 13 were prospective, and the remaining was a case-control study. Most studies investigated multiple potential risk factors for STH infection. Exposure status for WASH access and practices was typically determined through self-report, although 15 studies also used some form of observation to validate self-reported information. All included studies reported the diagnostic method used to assess helminth infection, with the Kato-Katz technique most frequently mentioned (*n* = 63). To assess the independent effect of WASH components on STH infection, authors typically used multiple regression analysis (*n* = 68), though adjusted effect estimates were often not reported for WASH covariates if they were not statistically significant. Not all multivariable models were reported with a full list of included covariates either. Slightly more than one-third of the studies (*n* = 33) reported at least one non-significant adjusted effect estimate. Study bias assessment is presented in Table S2. Meta-analysis results are available in Table 8 and grades summarized in Table 9.

**Table 8:** Meta-analysis results.

Meta-Analysis	Odds Ratio (95% CI)	Tau Squared	*Q p*-Value	*I* ^2^ (95% Uncertainty)	Egger's Test P	*n* Studies	GRADE
Piped water use (any STH)	0.93 (0.28–3.11)	1.86	<0.01	98.6 (98–99)	<0.01	5	Very low
Piped water use (*A. lumbricoides*)	0.40 (0.39–0.41)	0	0.62	0 (0–85)	0.08	4	Low
Piped water use (*T. trichiura*)	0.57 (0.45–0.72)	0	0.93	0 (0–90)	0.67	3	Low
Treated water use (any STH)	0.46 (0.36–0.60)	0	0.82	0 (0–90)	0.36	3	Low
Wearing shoes (hookworm)	0.29 (0.18–0.47)	0.09	0.09	30 (0–73)	0.03	5	Moderate
Wearing Shoes (any STH)	0.30 (0.11–0.83)	0.60	0.02	74 (12–92)	0.29	3	Low
Soap use/availability (any STH)	0.53 (0.29–0.98)	0.07	0.28	21 (0–92)	0.98	3	Low
Handwashing before eating (*A. lumbricoides*)	0.38 (0.26–0.55)	0	0.90	0 (0–90)	0.59	3	Low
Handwashing after defecation (*A. lumbricoides*)	0.45 (0.35–0.58)	0	0.55	0 (0–90)	0.29	3	Low
Handwashing after defecation (any STH)	0.47 (0.24–0.90)	0.44	<0.01	88 (74–94)	0.58	5	Very low
Sanitation access (any STH)	0.66 (0.57–0.76)	0	0.70	0 (0–68)	0.57	8	Low
Sanitation access (*T. trichiura*)	0.61 (0.50–0.74)	0.01	0.29	19 (0–62)	0.49	7	Low
Sanitation access (*A. lumbricoides*)	0.62 (0.44–0.88)	0.05	0.22	28 (0–70)	0.83	6	Low
Sanitation access (hookworm)	0.80 (0.61–1.06)	0.01	0.34	11 (0–77)	0.13	6	Very low

**Table 9:** Meta-analysis grades.

Meta-Analysis Group	Internal Bias	Inconsistency	Indirect	Imprecise	Publication Bias	Large Effect	Dose Response	Confounding Towards Null	Overall
Piped water access (any STH)	Moderate, used help of observations to assess exposure and used adjusted estimates	Yes, *I* ^2^ = 98.6%	Nothing serious	Yes, 95% CI includes null	Likely, but unclear due to strong heterogeneity	Nothing strong	Not found	Nothing strong	Very low, due to heterogeneity and wide confidence interval
Piped water access (*A. lumbricoides*)	Moderate, observational studies but all use adjusted estimates	*I* ^2^ = 0%, 95% CI (0%–85%)	Nothing serious	Nothing serious	Likely, but direction suggests slightly more protective effect	Nothing strong	Not found	Nothing strong	Low
Piped water access (*T. trichiura*)	Moderate, observational studies but all use adjusted estimates	*I* ^2^ = 0%, 95% CI (0%–90%)	Nothing serious	Nothing serious	Undetected	Nothing strong	Not found	Nothing strong	Low
Treated water use (any STH)	Moderate, observational studies but all use adjusted estimates	*I* ^2^ = 0%, 95% CI (0%–90%)	Nothing serious	Nothing serious	Undetected	Nothing strong	Not found	Nothing strong	Low
Wearing shoes (hookworm)	Moderate, observational studies but all use adjusted estimates	*I* ^2^ = 29.7%, 95% CI (0%–73%)	Nothing serious	Nothing serious	Likely	Strong effect evident (OR 0.29)	Not found	Yes, hygiene behaviors overreported	Moderate, due to strong effect size
Wearing shoes (any STH)	Moderate, observational studies but all use adjusted estimates	Yes, *I* ^2^ = 74%	Nothing serious	Nothing serious	Likely, but unclear due to strong heterogeneity	Strong effect evident (OR 0.30)	Not found	Yes, hygiene behaviors overreported	Low, upgraded from effect size, downgraded from heterogeneity
Soap use/availability (any STH)	Moderate, observational studies but all use adjusted estimates	*I* ^2^ = 20.8%, 95% CI (0%–92%)	Nothing serious	Nothing serious	Undetected	Nothing strong	Not found	Yes, hygiene behaviors overreported	Low
Handwashing before eating (*A. lumbricoides*)	Moderate, observational studies but all use adjusted estimates	*I* ^2^ = 0%, 95% CI (0%–90%)	Nothing serious	Nothing serious	Undetected	Nothing strong	Not found	Yes, hygiene behaviors overreported	Low
Handwashing after defecation (*A. lumbricoides*)	Moderate, observational studies but all use adjusted estimates	*I* ^2^ = 0%, 95% CI (0%–90%)	Nothing serious	Nothing serious	Undetected	Nothing strong	Not found	Yes, hygiene behaviors overreported	Low
Handwashing after defecation (any STH)	Moderate, observational studies but all use adjusted estimates	Yes - *I* ^2^ = 88%	Nothing serious	Yes, 95% CI includes null	Undetected	Nothing strong	Not found	Yes, hygiene behaviors overreported	Very low, due to high heterogeneity
Sanitation access (any STH)	Moderate, observational studies but all use adjusted estimates	*I* ^2^ = 0%, 95% CI (0%–68%)	Nothing serious	Nothing serious	Undetected	Nothing strong	N/A	Nothing strong	Low
Sanitation access (*T. trichiura*)	Moderate, observational studies but all use adjusted estimates	*I* ^2^ = 19%, 95% CI (0%–62%)	Nothing serious	Nothing serious	Undetected	Nothing strong	N/A	Nothing strong	Low
Sanitation access (*A. lumbricoides*)	Moderate, observational studies but all use adjusted estimates	*I* ^2^ = 28%, 95% CI (0%–70%)	Nothing serious	Nothing serious	Undetected	Nothing strong	N/A	Nothing strong	Low
Sanitation access (hookworm)	Moderately Low, used help of observations to assess exposure and used adjusted estimates	*I* ^2^ = 11%, 95% CI (0%–77%)	Nothing serious	Yes, 95% CI includes null	Likely, but direction suggests slightly more protective effect	Nothing strong	N/A	Nothing strong	Very low, due to confidence interval including null

### Water

**Figure 3:**
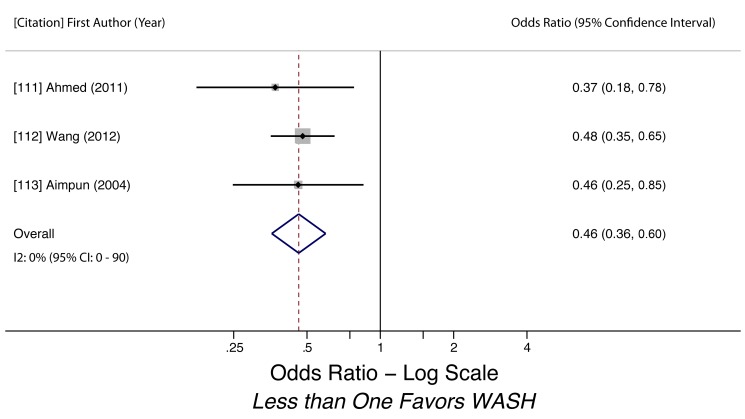
Meta-analysis of the association between use of treated water and infection with any STH [111]–[113].

**Figure 4:**
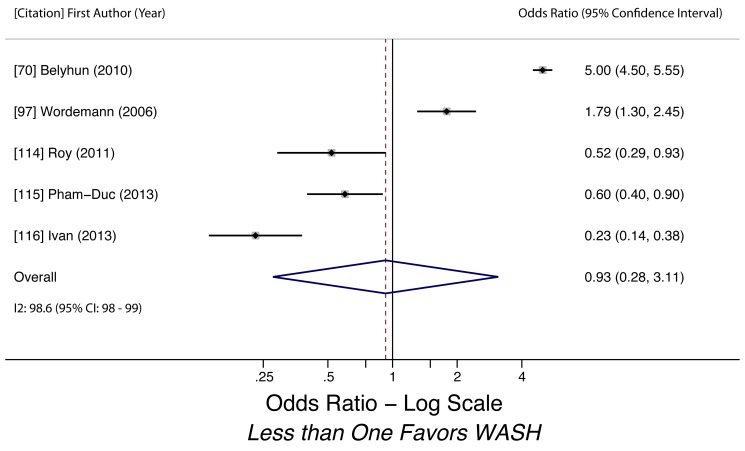
Meta-analysis of the association between use of piped water use and any STH infection [70],[97],[114]–[116].

**Figure 5:**
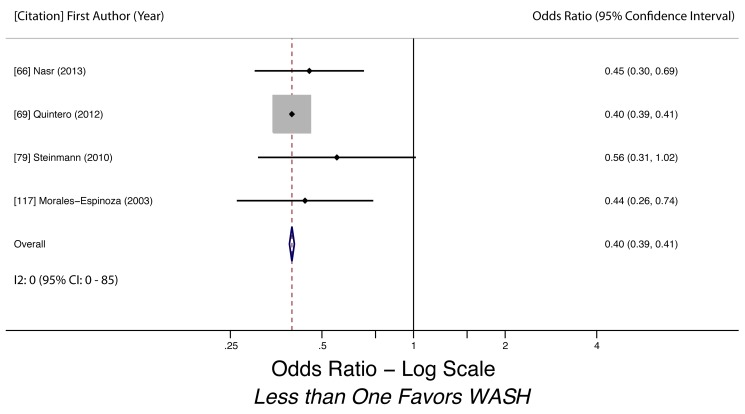
Meta-analysis of the association between use of piped water and *A. lumbricoides* infection [66],[69],[79],[117].

**Figure 6:**
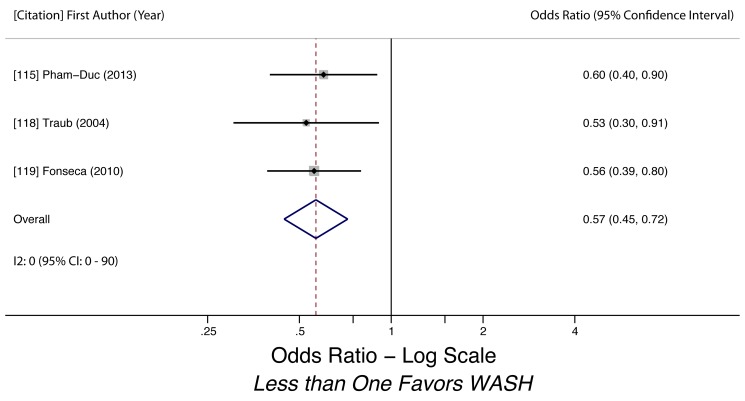
Meta-analysis of the association between use of piped water and *T. trichiura* infection [115],[118],[119].

Water-related access and practices were generally associated with lower odds of STH infection. We conducted meta-analyses to examine the association of piped water access and use of treated water on STH infection. Using treated water (filtered or boiled) was associated with lower likelihood of having any STH infection (k = 3, OR 0.46, 95% CI 0.36–0.60). The quality of evidence for the analysis was low, as all three studies were observational (Figure 3). Use of piped water was not associated with STH infection in general (k = 5, OR 0.93, 95% CI 0.28–3.11). The quality of evidence for the pooled estimate was very low due to high heterogeneity (*I^2^* = 98.6%, 95% CI 98%–99%, *Q p*-value<0.01) among the studies (Figure 4). The heterogeneity could have stemmed from multiple factors, as the five studies shared few methodological characteristics. Use of piped water was associated with reduced likelihood of *A. lumbricoides* infection (k = 4, OR 0.40, 95% CI 0.39–0.41) and *T. trichiura* infection (k = 3, OR 0.57, 95% CI 0.45–0.72). Evidence quality for these two meta-analyses was low, based on four studies and three studies respectively (Figures 5 and 6). We did not find a sufficient number of studies to conduct a similar meta-analysis for hookworm infection, although Nasr and colleagues found a significantly lower adjusted odds of infection (OR 0.59, 95% CI 0.34–0.91) for Malaysian children with access to piped water [66]. Other researchers found no statistically significant associations between piped water access and hookworm infection [67],[68].

Other water-related exposures for STH infection were reported in the literature, but not with sufficient frequency for meta-analyses. In one study examining storage of water, Quintero and colleagues found a significantly higher adjusted odds of *T. trichiura* infection for Venezuelan children and adults collecting water in “inappropriate” receptacles (OR 1.12, 95% CI 1.09–1.15) [69]. Limited evidence also was retrieved on the influence of water source location; Belyhun and colleagues [70] found a beneficial association of using an outside water pipe compared to an indoor tap for infection with any STH among Ethiopian infants (OR 0.21, 95% CI 0.09–0.51). Matthys and colleagues [71] found that having a private well significantly increased the odds of hookworm infection for farming households in western Côte d'Ivoire (OR 2.32, 95% CI 1.24–4.05). No evidence was found of an association between public or private water source and *S. stercoralis* infection [72]. Having “inadequate water supply” in schools was strongly associated with increased infection with any STH among school children living on Pacific islands (OR 4.93, 95% CI 2.24–10.88) [73].

### Sanitation

**Figure 7:**
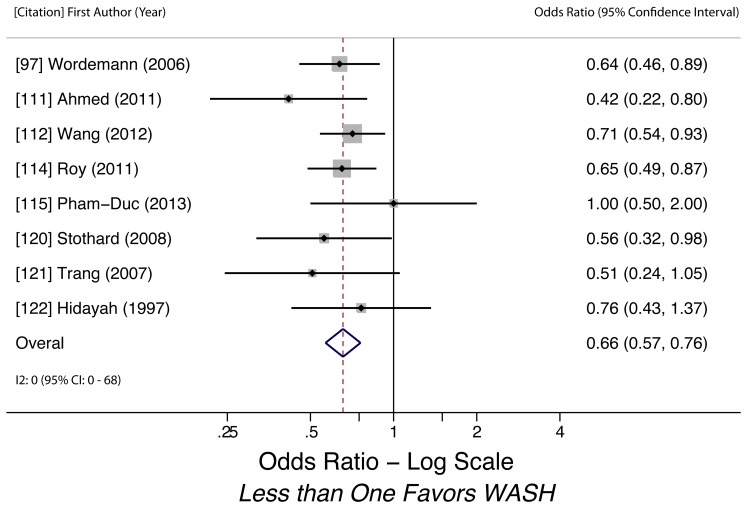
Meta-analysis of the association between sanitation access and infection with any STH [97],[111],[112],[114],[115],[120]–[122].

**Figure 8:**
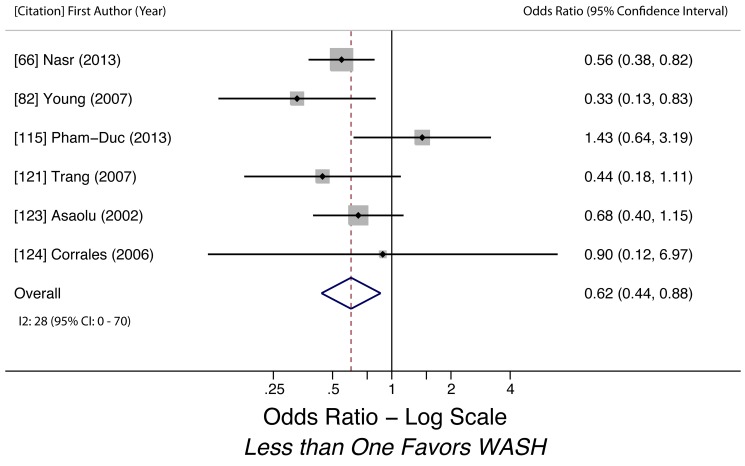
Meta-analysis of the association between sanitation access and *A. lumbricoides* infection [66],[82],[115],[121],[123],[124].

**Figure 9:**
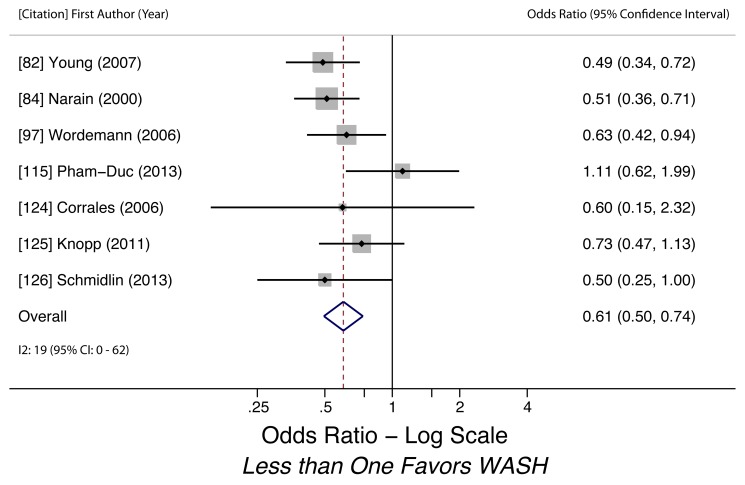
Meta-analysis of the association between sanitation access and *T. trichiura* infection [82],[84],[97],[115],[124]–[126].

**Figure 10:**
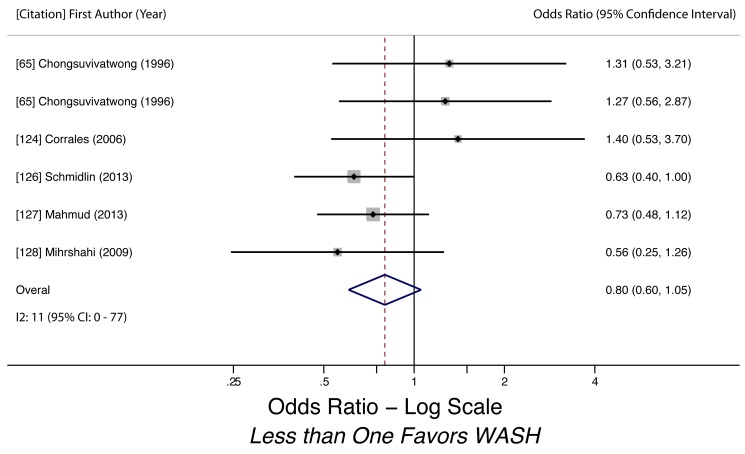
Meta-analysis of the association between sanitation access and hookworm infection [65],[124],[126]–[128]. Note: Chongsuvivatwong et al [65]. reported on two separate studies in their 1996 article.

Sanitation access (availability or use of latrines) was associated with lower likelihood of infection with any STH (k = 8, OR 0.66, 95% CI 0.57–0.76), *T. trichiura* (k = 7, OR 0.61, 95% CI 0.50–0.74), and *A. lumbricoides* (k = 6, OR 0.62, 95% CI 0.44–0.88) (Figures 7–9). The quality of evidence for these meta-analyses was low due to the observational nature of included studies. We did not find evidence that sanitation access was associated with hookworm infection (k = 6, OR 0.80, 95% CI 0.61–1.06), which had very low evidence quality due to imprecision (Figure 10).

We found limited evidence that use of shared or private sanitation facilities influenced odds of STH infection. Worrell and colleagues [74] found in Kenya that participants using toilets located outside of their household premises had significantly increased odds of infection with any STH. In contrast, another study found that sharing latrines with neighboring households, compared with private latrine use, was associated with significantly lower odds of hookworm infection [71]. Few details were provided to contextualize this finding.

### Hygiene

Three randomized controlled trials, two carried out in China and one in the Peruvian Amazon, found strong benefits for interventions that focused on promoting hygiene in schools [75]–[77]. Xu and colleagues [75] assessed a randomized intervention that promoted handwashing with soap, both before eating and after defecation among 657 school children in three schools. All infected children were treated at baseline. At the 1-year follow-up, *A. lumbricoides* prevalence for children in the experimental group had declined by 35.7% (pre-intervention prevalence, 68.3%; post-intervention cumulative infection rate, 43.9%) compared with an increase in the control group of 78% (pre-intervention, 41.4%; post-intervention, 73.7%); this was a statistically significant difference (*p*<0.01). The study's primary limitation was that schools were the unit of randomization, with two primary schools becoming controls and the third receiving the intervention. With so few clusters, it is highly possible that confounding factors were not comparable between the control and experimental groups.

More recently, Bieri and colleagues [76] reported on a single-blind, unmatched, cluster-randomized intervention trial involving 1,718 children (aged 9–10) in 38 schools over the course of one school year. Schools were randomly assigned to a health-education package, which included an entertainment-education cartoon video, or to a control package, which only displayed a health-education poster. All participants were treated with albendazole at baseline. At follow-up at the end of the school year, knowledge about STH was significantly higher in the intervention group, and almost twice as many intervention children (63.3% *versus* 33.4%, *p*<0.01) reported washing their hands after defecating. The incidence of STH infection (predominantly *T. trichiura* and *A. lumbricoides*) was also significantly improved in the experimental schools: 50% lower in the intervention group than in the control group (4.1% *versus* 8.4%, *p*<0.01).

Gyorkos and colleagues [77] conducted an open-label, cluster-randomized controlled trial using a hygiene education intervention in Peruvian primary schools. Within paired groups, 18 schools (1,089 fifth grade student participants) were randomly allocated to receive albendazole and the hygiene intervention or albendazole alone. The health intervention included a helminth-oriented class for students, a health curriculum workshop for teachers, and educational print materials. Four months after the intervention, the experimental group showed a significant reduction in *A. lumbricoides* intensity compared to deworming alone (adjusted incidence rate ratio [IRR] 0.42, 95% CI: 0.21–0.85). *T. trichiura* and hookworm intensity did not show statistically significant improvements in the experimental group, nor did prevalence of any single STH species. Children in the intervention group showed significant improvements in STH knowledge and water treatment behaviors compared to the control, but not in most other hygiene practices (e.g., handwashing). The authors also noted that the prevalence of hookworm was low (about 5% compared to 30% for *A. lumbricoides* and 50% for *T. trichiura*) and that albendazole was less efficacious against *T. trichiura* than it was against *A. lumbricoides*.

**Figure 11:**
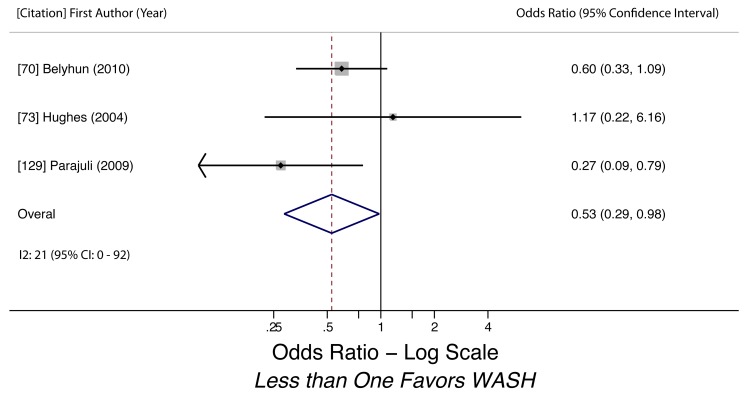
Meta-analysis of the association between soap use and infection with any STH [70],[73],[129].

**Figure 12:**
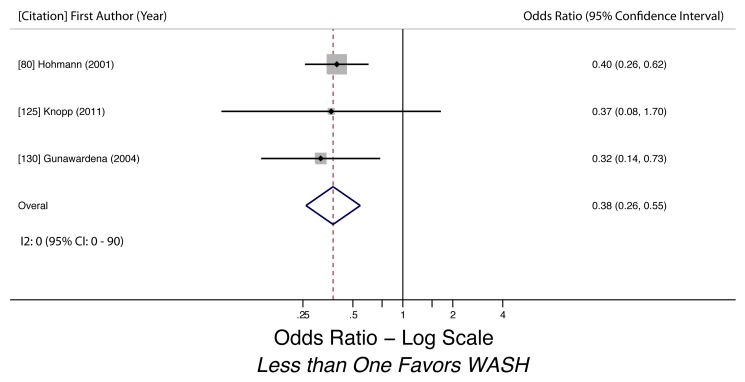
Meta-analysis of the association between handwashing before eating and infection with *A. lumbricoides* [80],[125],[130].

**Figure 13:**
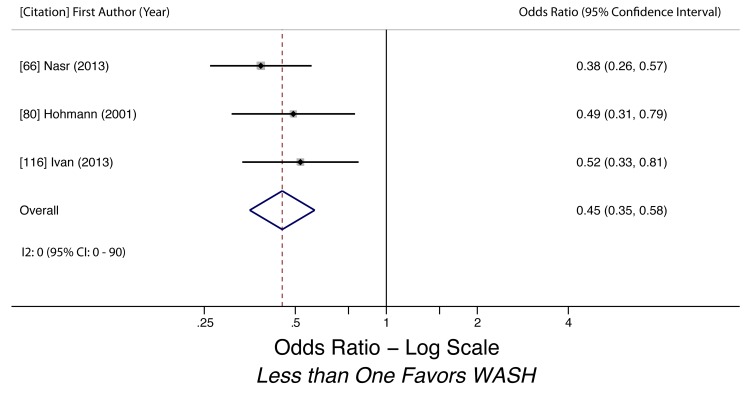
Meta-analysis of the association between handwashing after defecation and infection with *A. lumbricoides* [66],[80],[116].

**Figure 14:**
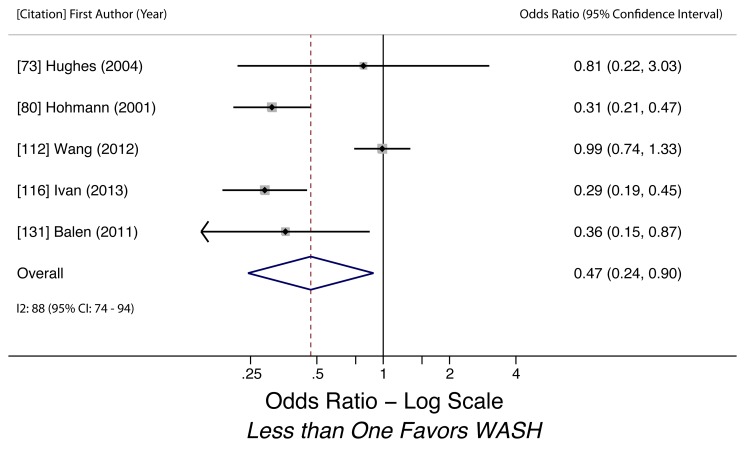
Meta-analysis of the association between handwashing after defecation and infection with any STH [73],[80],[112],[116],[131].

Our meta-analyses of hygiene-related observational evidence provided estimates that are consistent with findings from these randomized controlled trials. Soap use or availability was significantly associated with lower odds of STH infection at the 5% level (k = 3, OR 0.53, 95% CI 0.29–0.98). The quality of the evidence was low, though the possibility of respondents' over-reporting hygiene behaviors could have underestimated the strength of the association (Figure 11). Handwashing, both before eating (k = 3, OR 0.38, 95% CI 0.26–0.55) and after defecating (k = 3, OR 0.45, 95% CI 0.35–0.58), was associated with lower odds of *A. lumbricoides* infection (Figures 12 and 13). Both analyses were of low quality due to the observational evidence available. Handwashing after defecation also was associated with reduced odds of any STH infection (k = 5, OR 0.47, 95% CI 0.24–0.90). This meta-analysis had very low evidence quality due to high heterogeneity among estimates from the five pooled studies (*I* ^2^ = 88%, 95% CI 74%–94%, *Q p*-value<0.01, Figure 14). All studies used Kato-Katz for diagnosis, but varied considerably in most other study characteristics, including population age, baseline prevalence, and geographic setting. Balen and colleagues reported limited evidence of a dose-response effect for handwashing; respondents who more frequently washed their hands with soap after defecation had lower odds of infection with any STH, but confidence intervals of the handwashing groups overlapped [78].

Washing vegetables was found to be associated with lower odds of STH infection in two studies. Steinmann and colleagues [79] found washing vegetables to be negatively associated with *A. lumbricoides* infection in school children (OR 0.69, 95% CI 0.50–0.95), while Hohmann and colleagues [80] found washing was associated with lower odds of *T. trichiura* (OR 0.50, 95% CI 0.31–0.79) and any STH infection (OR 0.71, 95% CI 0.51–0.99).

**Figure 15:**
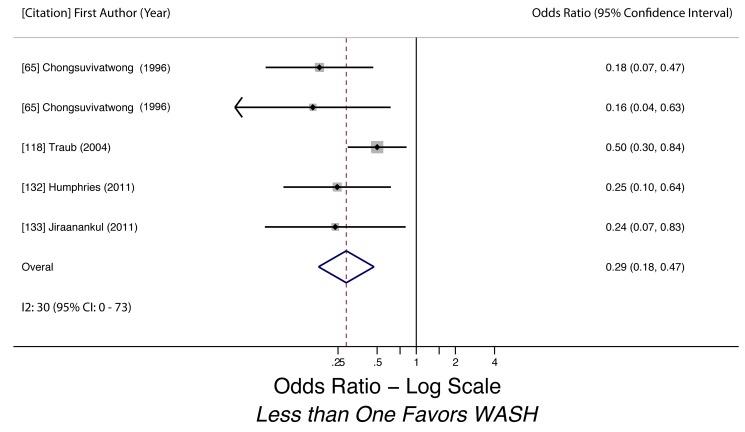
Meta-analysis of the association between wearing shoes and hookworm infection [65],[118],[132],[133]. Note: Chongsuvivatwong et al. [65] reported on two separate studies in their 1996 article.

**Figure 16:**
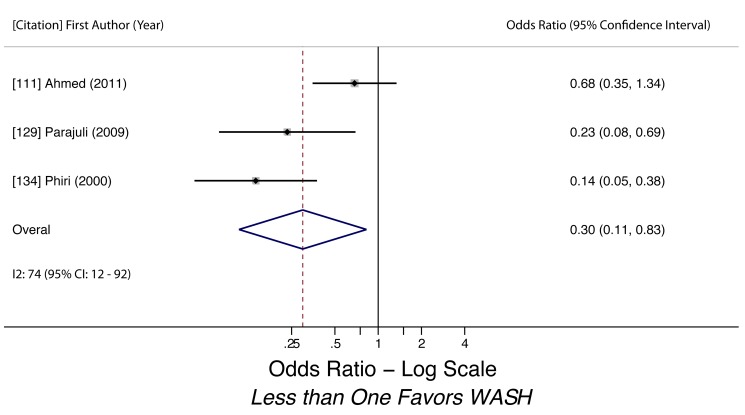
Meta-analysis of the association between wearing shoes and infection with any STH [111],[129],[134].

Our meta-analysis found evidence of a strong association between wearing shoes and lower odds of hookworm infection (k = 5, OR 0.29, 95% CI 0.18–0.47). The quality of the evidence was moderate, upgraded due to the magnitude of effect (Figure 15). Wearing shoes was also associated with lower odds of infection with any STH (k = 3, OR 0.30, 95% CI 0.11–0.83). The evidence quality for that analysis was low, downgraded by heterogeneity (*I^2^* = 74%, 95% CI 12–92%, *Q p*-value = 0.02) (Figure 16) but upgraded by a strong effect magnitude. Heterogeneity could have been introduced by many different factors, as the studies shared few characteristics. Three studies found mostly non-significant associations between geophagy (i.e., consumption of soil) and STH infection [81]–[83]. In adjusted models, households with dirt floors in India and Venezuela were found to have higher odds of *T. trichiura* and *A. lumbricoides* infection than were houses with other more elaborate flooring material [69],[84]. Young children living with dirt floors in Colombia also showed higher odds of infection with any STH compared to those with tile or cement floors [85].

### Integrated Interventions

In a cluster-randomized controlled trial, Freeman and colleagues examined a comprehensive WASH intervention in Kenyan schools that included hygiene promotion, water treatment and storage, and installation of sanitation infrastructure [27]. The intervention reduced reinfection prevalence (OR 0.56, 95% CI 0.31–1.00) and egg count (IRR 0.34, 95% CI 0.15–0.75) of *A. lumbricoides*, but not of *T. trichiura* or hookworm. Effects of the intervention differed by sex, with girls in the intervention group showing a significantly reduced *A. lumbricoides* infection intensity compared to the control group; boys in the intervention group did not show any significant difference from controls. Shoe-wearing and geophagy also emerged as effect modifiers for hookworm and *T. trichiura* infection intensity, respectively.

Dumba and colleagues found no statistically significant benefit of a participatory hygiene and sanitation transformation (PHAST) intervention when compared with a control group that only received deworming [86]. PHAST uses training sessions to encourage communities to identify problems in their own environment, decide what aspects need to be improved, and then implement changes. Parents or guardians of participating children in 19 villages received three PHAST education sessions. Participants in both control and experimental villages received albendazole and showed significant reductions in helminth prevalence compared with baseline, but the prevalence in the experimental group did not decline more than that among the control children. This study grouped *Hymenolepis nana* and *Enterobius vermicularis* with STH in analysis, but only a handful of participants were infected by *H. nana* or *E. vermicularis*, whereas STH prevalence was very high (>80%).

### *Strongyloides stercoralis*

We found 12 studies that investigated the relationship between WASH and *S. stercoralis* infection, but only located relevant effect estimates in five. Among school children in Cambodia, Khieu and colleagues found crude associations between infection and handwashing, shoe-wearing, and sanitation access [87]. Hall and colleagues found mixed results for a range of sanitation-related exposures, with some evidence that open defecation and use of community latrines were associated with higher odds of *S. stercoralis* infection in children [72]. In a multivariable model using data from a rural Peruvian community, Yori and colleagues found that wearing shoes never or occasionally (*versus* more frequently) was associated with higher odds of infection (OR 1.89, 95% CI 1.10–3.27) [88]. Knopp and colleagues did not find a significant association between *S. stercoralis* infection and home latrine ownership or handwashing after defecation [89].

## Discussion

We conducted a systematic review and meta-analysis of the relationship between WASH access and practices and STH infection. Our analysis revealed that WASH access and practices are generally, but not universally, associated with lower odds of STH infection. Particularly strong associations emerged between wearing shoes and hookworm infection (OR 0.29, 95% CI 0.18–0.47), piped water use and *A. lumbricoides* infection (OR 0.40, 95% CI 0.39–0.41), and treated water use and infection by any STH (OR 0.46, 95% CI 0.36–0.60). Pooled estimates for all meta-analyses, except for two (i.e., piped water use for any STH and sanitation access for hookworm), indicated at least a 33% lower odds of STH infection associated with specific WASH behaviors or access (Table 8). All but two of the meta-analyses were statistically significant at the 5% level.

On the basis of the evidence available, this review primarily draws upon observational studies. Observational research typically has greater risks to internal validity than randomized controlled trials, but such research is also key to providing a broad evidence base. When conducted well, randomized controlled trials provide the strongest evidence of a causal relationship between an exposure (e.g., an intervention) and an outcome. In the WASH context, however, conducting RCTs can be ethically and financially challenging. Traditional randomized designs can be costly and require that a subset of the target population be allocated to the control group, receiving only a limited intervention. Observational studies can be conducted more quickly and affordably in a wide array of contexts, allowing for WASH access and practices to be investigated in different social-ecological systems. This diversity is critical, since the effectiveness of specific WASH interventions can vary widely across settings, and interventions will most likely provide the greatest impact after being tailored to local conditions. Looking forward, a stepped wedge design represents a powerful compromise between ethics, operational feasibility, and internal validity. With a stepped wedge approach, the rollout of an intervention is randomized so that all participants eventually receive the study benefits, but at different times. Because many WASH interventions require staggered implementation owing to limited financial and human resources, randomizing the order in which communities are visited is often feasible. Combined with longitudinal data analysis, this design allows for robust assessments that can integrate with many interventions without radically altering implementing organizations' plans.

This review highlights important gaps in the WASH and STH body of literature. For example, only a few of the studies that met our inclusion criteria investigated the impact of sharing latrines (*n* = 6) or latrine maintenance (*n* = 3) on STH infection. The effect of treating water (*n* = 7) and geophagy (*n* = 10) were also infrequently explored. *S. stercoralis* was by far the least commonly investigated STH infection, reflecting another important knowledge gap.

A total of 35 studies contributed data to the 14 meta-analyses. A lack of standardized WASH definitions across studies limited our ability to pool results via additional meta-analyses. More consistent use of the Joint Monitoring Program's water and sanitation ladder definitions would aid future review efforts. Additional meta-analyses could have been conducted if all reviewed studies had provided relevant adjusted estimates of association. For example, many studies investigated the relationship between “toilet sharing” on any STH infection and “water access” on hookworm infection, but a dearth of reported adjusted estimates stymied meta-analyses of these relationships (Table 7).

Few studies analyzed the relationship between fecal egg count, a proxy for intensity of infection, and WASH [27],[81],[90], even though intensity of infection represents a more relevant predictor for morbidity than prevalence alone [91]. A lack of measures on this relationship represents a considerable gap in the literature, though many studies did report broadly on intensity of infection. Zero-inflated modeling strategies have recently shown promise in analyzing fecal egg count datasets, which often contain excess zero counts due to some individuals not harboring infections [92]–[94]. Contemporary analysis of existing data represents a potentially cost-effective mechanism for yielding additional insights into this topic.

Our findings build upon past reviews by Asaolu and Ofoezie [32] and Ziegelbauer and colleagues [26], which both concluded that WASH represents a valuable strategy for STH control. Although Asaolu and Ofoezie did not conduct a meta-analysis, their comprehensive review found broad evidence of reductions in STH prevalence and intensity resulting from multiple types of WASH interventions. Asaolu and Ofoezie concluded that improvements in sanitation systems and hygiene practices were important tools to not only sustain preventive chemotherapy benefits, but also help protect the uninfected. Results from our meta-analyses support their conclusion using systematically aggregated quantitative data. Ziegelbauer and colleagues focused more specifically on latrine access and use, conducting a rigorous meta-analysis using primarily crude odds ratios. The results from our meta-analyses, which drew upon adjusted odds ratios, are consistent with their findings and lend additional support to the value of sanitation improvements for STH control. Our meta-analyses also broadened focus to include water and hygiene components, allowing for a quantitative summary of currently available evidence across the three core WASH domains.

Our analysis of the relationship between access to a piped water source and STH infection yielded significantly protective associations for *A. lumbricoides* and *T. trichiura*, but not for any STH infection generally. The meta-analysis of any STH yielded strong heterogeneity statistics, reflecting a spread in observed effects. While the inclusion of hookworm infections in the “any STH” analysis may seem like a possible source of the variability, we found no clear evidence to support this explanation. The only study that analyzed hookworm infection and piped water use with an adjusted model found a significantly protective association, so other sources of heterogeneity should be considered.

The presence of heterogeneity can be systematically investigated by statistics like Moran's *I* ^2^ and Cochran's *Q*, but these global tests do not themselves uncover specific causes of heterogeneity. Diversity among studies can originate from a plethora of sources: population, setting, diagnostic approach, study design, analytic method, definitions, and so on. Without additional subgroup analysis or meta-regression, which both require a large body of studies, it is difficult to investigate the myriad potential causes of heterogeneity. Without clarification, the presence of heterogeneity indicates that pooled results are averaging multiple related, but distinct effects. For example, access to piped water could have different levels of benefit depending on distance to the source [95],[96], water quality [70],[97], or other unknown factors—especially when studies use different diagnostic assays and are conducted in a variety of community settings.

Concerning sanitation, our meta-analyses of access to sanitation yielded considerably lower odds of infection with *A. lumbricoides*, *T. trichiura*, or any STH for those with latrine access. We did not find evidence of a statistically significant association between sanitation and hookworm, though the pooled estimate suggested reduced odds of infection. Our sanitation findings were comparable to those found by Ziegelbauer and colleagues, who asserted that improved sanitation access should be prioritized alongside preventive chemotherapy to achieve a sustainable reduction in helminthiasis burden. They found an overall pooled odds ratio of 0.51 (95% CI 0.44–0.61) for the effect of sanitation availability and use, while we found an odds ratio of 0.66 (95% CI 0.57–0.76). Species-specific results were similar as well, with the exception of hookworm. Differences in the magnitude of our findings may be attributed to the use of adjusted measures in our analysis, since Ziegelbauer and colleagues used unadjusted estimates. In addition, we did not include separate estimates for sanitation use and access. Taken together, these two reviews support the hypothesis that improved access to, and use of, sanitation prevents STH infection. Additional research could help explore the complementarity of sanitation promotion with MDA.

For hygiene, three randomized controlled trials provided strong evidence linking hygiene practices—especially handwashing with soap—to reductions in STH infection [75]–[77]. However, not all hygiene interventions may be effective in reducing STH infection [86]. Our meta-analyses of the effect of handwashing before eating and after defecation for *A. lumbricoides* infection, along with handwashing after defecation and soap use for any STH infection, also yielded significant results that suggest protective effects. Accurately assessing handwashing is challenging; self-reported and observed measures are often highly biased [33]. Many studies rely on self-report, but individuals have consistently been shown to over-report handwashing behaviors [98]. Heterogeneity was exhibited in the analysis of handwashing after defecation, suggesting that the benefits of handwashing may vary considerably depending on circumstances and definitions. Beyond handwashing, our analysis also showed that wearing shoes was associated with significantly lower odds of infection with hookworm and any STH.

These results may be of interest to several audiences. Researchers can take note of the gaps in the literature identified by this review and focus investigation on key outstanding questions (e.g., the impact of WASH on *S. stercoralis* infections). Policymakers should understand that, despite gaps in data, these findings provide a broad evidence base in support of WASH for STH control—especially from randomized trials for hygiene interventions. WASH practitioners will recognize that these findings provide further support for their efforts and, we hope, will consider partnering with STH researchers to evaluate future interventions.

### Strengths and Limitations

Our review included only adjusted effect estimates in meta-analyses, which lends greater strength to our pooled results [37]. Many different variables were controlled across studies, which may contribute to heterogeneity. However, this variation in adjusted models may also serve as a small buffer against the inherent heterogeneity across observational studies. Different covariates will vary in importance for different populations and circumstances, so a broad review like ours may benefit from pooling estimates from models that were adapted by researchers to best fit their data and contexts. There are many factors that could confound the relationship between WASH access or practices and STH prevalence, including socioeconomic status, age, and gender. Consideration of only crude associations would likely overstate the magnitude of effect for WASH exposures or even misinterpret the true direction of effect [99]. Limiting our focus to adjusted measures of effect reduces the number of eligible studies, which may impact the generalizability of our results. This strategy also amplifies the impact of selective reporting, since many authors reported only statistically significant adjusted estimates.

Evidence quality was typically “low”—the default GRADE for observational research—meaning that our confidence in pooled effect estimates is limited, and that the true effect may be markedly different from the results reported here [40]. A much stronger case can be made for the benefit of hygiene because of the evidence provided by recent randomized controlled trials, but results from our meta-analyses suggest that the protective effect of hygiene practices on STH infection may be variable depending on context.

Publication bias also represents a concern. Five meta-analyses (piped water for any STH and *A. lumbricoides*, wearing shoes for hookworm and any STH, sanitation access for hookworm) showed evidence of publication bias in funnel plot assessments. However, two of those plots (piped water for *A. lumbricoides* and sanitation access for hookworm) showed that larger studies yielded more protective associations, suggesting that the results from those analyses may be underestimating the true relationship strength. This was unexpected—and possibly caused by the natural heterogeneity across observational studies—since larger studies are traditionally expected to show smaller magnitudes of effect. Heterogeneity creates great difficulty in assessing publication bias accurately with statistical tests, so it is impossible to know how pronounced publication bias may be throughout our meta-analyses [100].

### Conclusion

A vibrant discussion continues in the literature about the role of MDA in measurably mitigating morbidity from STH infection at the population level [101]–[106]. MDA alone is unlikely to permanently interrupt STH transmission. Our review provides evidence that WASH is a valuable component for STH control strategies, but guidelines and targets for the integration of these approaches are needed. Increased attention towards WASH for STH also has great potential to catalyze synergies with integrated NTD control programs, while jointly elevating awareness of WASH and NTDs [5],[28],[107]. Additional high-quality research into the potential of integrated WASH interventions is merited, specifically on the complementarity of WASH and MDA. Recent and ongoing research continues to build an evidence-base that can guide policymaking and programmatic decisions [27],[28],[108]. Increased collaboration between the health and WASH sectors represents a key enterprise for the future of NTD control and elimination [109],[110].

## Supporting Information

Figure S1 **Funnel plot for treated water use and any STH infection.**
(EPS)

Figure S2 **Funnel plot for piped water use and any STH infection.**
(EPS)

Figure S3 **Funnel plot for piped water use and** ***A. lumbricoies*** **infection.**
(EPS)

Figure S4 **Funnel plot for piped water use and** ***T. trichiura*** **infection.**
(EPS)

Figure S5 **Funnel plot for sanitation access and any STH infection.**
(EPS)

Figure S6 **Funnel plot for sanitation access and** ***A. lumbricoides*** **infection.**
(EPS)

Figure S7 **Funnel plot for sanitation access and** ***T. trichiura*** **infection.**
(EPS)

Figure S8 **Funnel plot for sanitation access and hookworm infection.**
(EPS)

Figure S9 **Funnel plot for soap use and any STH infection.**
(EPS)

Figure S10 **Funnel plot for handwashing before eating and** ***A. lumbricoides*** **infection.**
(EPS)

Figure S11 **Funnel plot for handwashing after defecating and** ***A. lumbricoides*** **infection.**
(EPS)

Figure S12 **Funnel plot for handwashing after defecating and any STH infection.**
(EPS)

Figure S13 **Funnel plot for wearing shoes and hookworm infection.**
(EPS)

Figure S14 **Funnel plot for wearing shoes and any STH infection.**
(EPS)

Table S1 **Excluded studies.**
(DOC)

Table S2 **Study bias assesment.**
(DOC)

Text S1 **PRISMA checklist.**
(DOC)

Text S2 **MOOSE checklist.**
(DOC)

Text S3 **Original methods protocol.**
(DOC)

## Abbreviations

GRADE: Grades of Recommendation, Assessment, Development and Evaluation
MDA: mass drug administration
NTD: neglected tropical disease
OR: odds ratio
PHAST: participatory hygiene and sanitation transformation
RR: risk ratio
STH: soil-transmitted helminth
WASH: water, sanitation, and hygiene
WHO: World Health Organization
