# Supplementary material for: Water, Sanitation, Hygiene, and Soil-Transmitted Helminth Infection: A Systematic Review and Meta-Analysis
Source: PLoS Med. 2014 Mar 25;11(3):e1001620. doi: 10.1371/journal.pmed.1001620 (PMC3965411; doi:10.1371/journal.pmed.1001620)
Supplement: Table S1 — Excluded studies. (DOC) [file pmed.1001620.s015.doc]

## Table S1. Excluded studies with rationale

| **Citation** | **Author, Year** | **Reason for Exclusion** |
| --- | --- | --- |
| [1] | Aagard-Hansen et al., 2010 | No relevant effect measure |
| [2] | Abanyie et al., 2013 | No relevant effect measure |
| [3] | Abera et al., 2013 | Grouped outcome data |
| [4] | Abou-Zeid et al., 2012 | No relevant effect measure |
| [5] | Abu Mourad et al., 2004 | No relevant effect measure |
| [6] | Acka et al., 2010 | No relevant effect measure |
| [7] | Adams et al., 2005 | No relevant effect measure |
| [8] | Adeoye et al., 2007 | No relevant effect measure |
| [9] | Agbaya et al., 2004 | No relevant effect measure |
| [10] | Agi, 1995 | No relevant effect measure |
| [11] | Agi, 1997 | No relevant effect measure |
| [12] | Ahmed et al., 2004 | No relevant effect measure |
| [13] | Akinbo et al., 2010 | No relevant effect measure |
| [14] | Akinbo et al., 2013 | No relevant effect measure |
| [15] | Alaofe et al., 2008 | Grouped outcome data |
| [16] | AlBallaa et al., 1993 | No relevant effect measure |
| [17] | Al-Ballaa et al., 1993 | No relevant effect measure |
| [18] | Albright et al., 2005 | No relevant effect measure |
| [19] | Alemu et al., 2012 | Grouped outcome data |
| [20] | Ali-Shtayeh et al., 1989 | No relevant effect measure |
| [21] | Al-Khatib et al., 2003 | No relevant effect measure |
| [22] | Al-Madani et al., 1989 | No relevant effect measure |
| [23] | Almeida et al., 2003 | No relevant effect measure |
| [24] | Al-Mohammed et al., 2010 | Grouped outcome data |
| [25-28] | Alyousefi et al., 2011 | Grouped outcome data |
| [29] | Amuta et al., 2010 | No relevant effect measure |
| [30] | Anantaphruti et al., 2000 | No relevant effect measure |
| [31] | Anantaphruti et al., 2004 | No relevant effect measure |
| [32] | Aramayo et al., 2009 | No relevant effect measure |
| [33] | Arfaa et al., 1976 | No relevant effect measure |
| [34] | Arfaa et al., 1977 | No relevant effect measure |
| [35] | Asaolu and Ofoezie, 2003 | Review or opinion article |
| [36] | Atukorala et al., 1999 | No relevant effect measure |
| [37] | Ayanwale et al., 1982 | No relevant effect measure |
| [38] | Bagchi et al., 1964 | No relevant effect measure |
| [39] | Bailey et al., 2013 | No relevant effect measure |
| [40] | Banbula et al., 1970 | No relevant effect measure |
| [41] | Banbula et al., 1971 | No relevant effect measure |
| [42] | Barrett et al., 2008 | No relevant effect measure |
| [43] | Basualdo et al., 2007 | No relevant effect measure |
| [44] | Baxter et al., 1994 | No relevant effect measure |
| [45] | Behnke et al., 2000 | No relevant effect measure |
| [46] | Belo et al., 2012 | No relevant effect measure |
| [47] | Bethony et al., 2002 | No relevant effect measure |
| [48] | Bhandari et al., 2011 | No relevant effect measure |
| [49] | Bidinger et al., 1981 | No relevant effect measure |
| [50] | Bisht et al., 2011 | No relevant effect measure |
| [51] | Bisi-Johnson et al., 2010 | No relevant effect measure |
| [52] | Blumenthal et al., 1996 | No relevant effect measure |
| [53] | Blumenthal et al., 2001 | No relevant effect measure |
| [54] | Boccaccio, 1972 | No relevant effect measure |
| [55] | Boia et al., 1999 | No relevant effect measure |
| [56] | Borda et al., 1996 | No relevant effect measure |
| [57] | Bosman et al., 1991 | No relevant effect measure |
| [58] | Bouchet et al., 1986 | No relevant effect measure |
| [59] | Bouhoum et al., 2000 | No relevant effect measure |
| [60] | Bouree et al., 1984 | No relevant effect measure |
| [61] | Bradley et al., 1993 | No relevant effect measure |
| [62] | Branco et al., 2012 | No relevant effect measure |
| [63] | Breitling et al., 2008 | No relevant effect measure |
| [64] | Brocklehurst et al., 2010 | No relevant effect measure |
| [65] | Brown et al., 2013 | No relevant effect measure |
| [66] | Buck et al., 1978 | No relevant effect measure |
| [67] | Bunnag et al., 1980 | No relevant effect measure |
| [68] | Cai et al., 2002 | No relevant effect measure |
| [69] | Cancrini et al., 1988 | No relevant effect measure |
| [70] | Cancrini et al., 1989 | No relevant effect measure |
| [71] | Cañete et al., 2012 | No relevant effect measure |
| [72] | Carlton et al., 2012 | No relevant effect measure |
| [73] | Carvalho and Teodosio, 2011 | No relevant effect measure |
| [74] | Chandiwana et al., 1989 | No relevant effect measure |
| [75] | Chandler et al., 1954 | No relevant effect measure |
| [76] | Chaskar et al., 1996 | No relevant effect measure |
| [77] | Chirdan et al., 2010 | Grouped outcome data |
| [78] | Chunge et al., 1992 | No relevant effect measure |
| [79] | Cifuentes et al., 1994 | No relevant effect measure |
| [80] | Cifuentes, 1998 | No relevant effect measure |
| [81] | Clark et al., 2006 | No relevant effect measure |
| [82] | Clasen et al., 2012 | No relevant effect measure |
| [83] | Clennon et al., 2010 | No relevant effect measure |
| [84] | Cremades Romero et al., 1997 | No relevant effect measure |
| [85] | Cruz et al., 2002 | No relevant effect measure |
| [86] | Curtale et al., 1998 | Grouped outcome data |
| [87] | Curtale et al., 1999 | No relevant effect measure |
| [88] | Dancesco et al., 2005 | No relevant effect measure |
| [89] | Daryani et al., 2012 | No relevant effect measure |
| [90] | de Silva et al., 1996 | No relevant effect measure |
| [91] | Dumba et al., 2008 | No relevant effect measure |
| [92] | Dunn, 1972 | No relevant effect measure |
| [93] | Ekpo et al., 2008 | No relevant effect measure |
| [94] | El Kettani et al., 2008 | No relevant effect measure |
| [95] | Elmadhoun et al., 2013 | No relevant effect measure |
| [96] | Embil et al., 1984 | No relevant effect measure |
| [97] | Ensink et al, 2005 | No relevant effect measure |
| [98] | Ensink et al., 2008 | No relevant effect measure |
| [99] | Erko and Tedla, 1993 | No relevant effect measure |
| [100] | Escobedo et al., 2008 | Grouped outcome data |
| [101] | Espinoza et al., 2012 | No relevant effect measure |
| [102] | Esrey et al., 1991 | Review or opinion article |
| [103] | Ezeagwuna et al., 2010 | No relevant effect measure |
| [104] | Fashuyi et al., 1988 | No relevant effect measure |
| [105] | Fatiregun et al., 2008 | No relevant effect measure |
| [106] | Fattal et al., 1988 | No relevant effect measure |
| [107] | Ferrell, 1920 | No relevant effect measure |
| [108] | Flohr et al., 2006 | No relevant effect measure |
| [109] | Fort, 1915 | No relevant effect measure |
| [110] | Fouamno et al., 2011 | No relevant effect measure |
| [111] | Fuentes et al., 2011 | No relevant effect measure |
| [112] | Fung and Cairncross, 2009 | Review or opinion article |
| [113] | Gamboa et al., 1998 | No relevant effect measure |
| [114] | Gamboa et al., 2003 | No relevant effect measure |
| [115] | Gamboa et al., 2009 | No relevant effect measure |
| [116] | Gamboa et al., 2011 | No relevant effect measure |
| [117] | Gamboa et al., 2012 | No relevant effect measure |
| [118] | Garg et al., 1984 | No relevant effect measure |
| [119] | Gelaw et al., 2013 | Grouped outcome data |
| [120] | Getachew et al., 2013 | No relevant effect measure |
| [121] | Gross et al., 1989 | No relevant effect measure |
| [122] | Gryseels et al., 1985 | No relevant effect measure |
| [123] | Gumbo et al., 2010 | No relevant effect measure |
| [124] | Gungoren et al., 2007 | Grouped outcome data |
| [125] | Gyawali et al., 2009 | No relevant effect measure |
| [126] | Gyorkos et al., 1996 | No relevant effect measure |
| [127] | Gyoten et al., 2010 | No relevant effect measure |
| [128] | Habbari et al., 1999 | No relevant effect measure |
| [129] | Habbari et al., 2000 | No relevant effect measure |
| [130] | Hagel et al., 1993 | No relevant effect measure |
| [131] | Handzel et al., 2003 | No relevant effect measure |
| [132] | Herrera et al., 2006 | No relevant effect measure |
| [133] | Heukelbach et al., 2008 | No relevant effect measure |
| [134] | Holland et al., 1988 | No relevant effect measure |
| [135] | Hosain et al., 2003 | No relevant effect measure |
| [136] | Humphries et al., 1997 | No relevant effect measure |
| [137] | Hung et al., 2005 | Grouped outcome data |
| [138] | Hunt et al., 2001 | Review or opinion article |
| [139] | Huruy et al., 2011 | No relevant effect measure |
| [140] | Hussein, 2011 | Grouped outcome data |
| [141] | Huttly et al., 1990 | Review or opinion article |
| [142] | Idowu et al., 2006 | No relevant effect measure |
| [143] | Idowu et al., 2011 | No relevant effect measure |
| [144] | Ikeh et al., 2011 | No relevant effect measure |
| [145] | Ilechukwu et al., 2011 | Grouped outcome data |
| [146] | Ishiyama et al., 2006 | No relevant effect measure |
| [147] | Jacobsen et al., 2007 | No relevant effect measure |
| [148] | Jombo et al., 2007 | Grouped outcome data |
| [149] | Jombo et al., 2010 | No relevant effect measure |
| [150] | Kaminsky, 1991 | No relevant effect measure |
| [151] | Karaman et al., 2006 | No relevant effect measure |
| [152] | Karan et al., 2012 | No relevant effect measure |
| [153] | Kawi et al., 2009 | No relevant effect measure |
| [154] | Kettani and Azzouzi, 2006 | No relevant effect measure |
| [155] | Kightlinger et al., 1998 | No relevant effect measure |
| [156] | King et al., 2013 | No relevant effect measure |
| [157] | Kipyegen et al., 2012 | Grouped outcome data |
| [158] | Kitvatanachai et al., 2008 | Grouped outcome data |
| [159] | Kloos et al., 1981 | No relevant effect measure |
| [160] | Koga-Kita et al., 2004 | No relevant effect measure |
| [161] | Komiya and Kunii, 1998 | No relevant effect measure |
| [162] | Kosoff et al., 1989 | No relevant effect measure |
| [163] | Krause et al., 2012 | No relevant effect measure |
| [164] | Kroeger et al., 1992 | No relevant effect measure |
| [165] | Kurniawan, 2012 | No relevant effect measure |
| [166] | Lansdown et al., 2002 | No relevant effect measure |
| [167] | Lewin et al., 2007 | Grouped outcome data |
| [27] | Li et al., 2011 | No relevant effect measure |
| [168] | Lin et al., 2008 | No relevant effect measure |
| [169] | llechukwu et al., 2010 | No relevant effect measure |
| [170] | Lura et al., 2002 | No relevant effect measure |
| [171] | Machado et al., 1999 | No relevant effect measure |
| [172] | Magalhães et al., 2011 | Review or opinion article |
| [173] | Magnussen et al., 1998 | No relevant effect measure |
| [174] | Mahfouz et al., 1997 | Grouped outcome data |
| [175] | Maia et al., 2009 | Grouped outcome data |
| [176] | Mamunur et al., 2011 | No relevant effect measure |
| [177] | Mangali et al., 1993 | No relevant effect measure |
| [178] | Mangali et al., 1994 | No relevant effect measure |
| [179] | Mara and Sleigh, 2010 | No relevant effect measure |
| [180] | Mascarini-Serra et al., 2012 | Review or opinion article |
| [181] | Matthys et al., 2011 | No relevant effect measure |
| [182] | Mboera et al., 2011 | No relevant effect measure |
| [183] | McMullen et al., 2012 | Review or opinion article |
| [184] | Menan et al., 1997 | No relevant effect measure |
| [185] | Midzi et al., 2011 | No relevant effect measure |
| [186] | Mil et al., 2011 | No relevant effect measure |
| [187] | Minamoto et al., 2012 | No relevant effect measure |
| [188] | Missaye et al., 2013 | Grouped outcome data |
| [188] | Missaye et al., 2013 | Grouped outcome data |
| [189] | Monse et al., 2013 | No relevant effect measure |
| [190] | Moore et al., 1965 | No relevant effect measure |
| [191] | Mora et al., 2009 | No relevant effect measure |
| [192] | Mordi et al., 2009 | No relevant effect measure |
| [193] | Moubarrad et al., 2005 | No relevant effect measure |
| [194] | Moulinier et al., 1990 | No relevant effect measure |
| [195] | Muller et al., 1989 | No relevant effect measure |
| [196] | Nasr et al., 2013 | No relevant effect measure |
| [197] | Navarrete et al., 1994 | No relevant effect measure |
| [25] | Nematian et al., 2004 | Grouped outcome data |
| [198] | Ngui et al., 2011 | Grouped outcome data |
| [199] | Nitulescu et al., 1954 | No relevant effect measure |
| [200] | Nkenfou et al., 2013 | Grouped outcome data |
| [201] | Oberg et al., 1993 | No relevant effect measure |
| [202] | Obiamiwe et al., 1977 | No relevant effect measure |
| [203] | Okyay et al., 2004 | No relevant effect measure |
| [204] | Opara et al., 2005 | No relevant effect measure |
| [205] | Ozturk et al., 2004 | No relevant effect measure |
| [206] | Ozumba et al., 2005 | No relevant effect measure |
| [207] | Pegelow et al., 1997 | No relevant effect measure |
| [208] | Pengpidand Peltzer, 2012 | No relevant effect measure |
| [209] | Perera et al., 2012 | No relevant effect measure |
| [210] | Pezzani et al., 1996 | No relevant effect measure |
| [211] | Pezzani et al., 2009 | No relevant effect measure |
| [212] | Pinheiro et al., 2011 | Grouped outcome data |
| [213] | Pruss et al., 2002 | Grouped outcome data |
| [214] | Pullan et al., 2008 | No relevant effect measure |
| [215] | Pullan et al., 2011 | No relevant effect measure |
| [216] | Quihui et al., 2006 | Grouped outcome data |
| [217] | Rai et al., 1997 | No relevant effect measure |
| [218] | Rai et al., 2004 | No relevant effect measure |
| [219] | Rai et al., 2008 | No relevant effect measure |
| [220] | Raja et al., 2001 | No relevant effect measure |
| [221] | Raja'a et al., 2001 | No relevant effect measure |
| [222] | Rajeswari et al., 1994 | No relevant effect measure |
| [223] | Ramesh et al., 1991 | No relevant effect measure |
| [224] | Ratard et al., 1991 | No relevant effect measure |
| [225] | Rebollo et al., 2011 | No relevant effect measure |
| [226] | Reinhard et al., 1994 | No relevant effect measure |
| [227] | Righetti et al., 2012 | No relevant effect measure |
| [228] | Rinne et al., 2005 | No relevant effect measure |
| [229] | Roka et al., 2012 | No relevant effect measure |
| [230] | Rollemberg et al., 2010 | No relevant effect measure |
| [231] | Rostami et al., 2011 | No relevant effect measure |
| [232] | Ruiz Lopes et al., 2006 | Grouped outcome data |
| [233] | Ryngajllo et al., 2008 | No relevant effect measure |
| [234] | Saathoff et al., 2005 | No relevant effect measure |
| [235] | Sadaga and Kassem, 2007 | No relevant effect measure |
| [236] | Sadek et al., 1997 | No relevant effect measure |
| [237] | Sadun et al., 1954 | No relevant effect measure |
| [238] | Salazar-Labori et al., 2011 | No relevant effect measure |
| [239] | Sanches and Wagner, 1954 | No relevant effect measure |
| [240] | Sanchez et al., 2001 | No relevant effect measure |
| [241] | Schlosser et al., 1999 | Grouped outcome data |
| [242] | Scholte et al., 2012 | No relevant effect measure |
| [243] | Schonning et al., 2006 | No relevant effect measure |
| [244] | Seidu et al., 2008 | No relevant effect measure |
| [245] | Shakya et al., 2012 | No relevant effect measure |
| [246] | Shang et al., 2010 | No relevant effect measure |
| [247] | Sharma et al., 2004 | No relevant effect measure |
| [248] | Shobha et al., 2013 | No relevant effect measure |
| [249] | Shrestha et al., 2012 | No relevant effect measure |
| [250] | Siharath et al., 2001 | No relevant effect measure |
| [251] | Silva et al., 2007 | No relevant effect measure |
| [252] | Silva et al., 2011 | No relevant effect measure |
| [253] | Singh et al., 2011 | No relevant effect measure |
| [254] | Smith et al., 2001 | No relevant effect measure |
| [255] | Sobrinho et al., 1995 | No relevant effect measure |
| [256] | Sorensen et al., 1994 | No relevant effect measure |
| [257] | Soroczan et al., 1976 | No relevant effect measure |
| [258] | Sultana et al., 2012 | No relevant effect measure |
| [259] | Suputtamongkol et al., 2012 | No relevant effect measure |
| [260] | Tagajdid et al., 2011 | No relevant effect measure |
| [261] | Tamer et al., 2008 | No relevant effect measure |
| [262] | Tang et al., 1993 | No relevant effect measure |
| [263] | Tanner et al., 2011 | No relevant effect measure |
| [264] | Taranto et al., 2003 | No relevant effect measure |
| [265] | Tashima et al., 2004 | No relevant effect measure |
| [266] | Tatala et al., 2009 | No relevant effect measure |
| [267] | Tavares-Dias et al., 1999 | No relevant effect measure |
| [268] | Tellez et al., 1997 | No relevant effect measure |
| [269] | Temcharoen et al., 1987 | Grouped outcome data |
| [270] | Thanchomnang et al., 2011 | No relevant effect measure |
| [271] | Thofern et al., 1989 | Review or opinion article |
| [272] | Tikasingh et al., 2011 | No relevant effect measure |
| [273] | Toma et al., 1999 | No relevant effect measure |
| [274] | Tomono et al., 2003 | No relevant effect measure |
| [275] | Torres et al., 1997 | No relevant effect measure |
| [276] | Tronnberg et al., 2010 | No relevant effect measure |
| [277] | Tshikuka et al., 1995 | No relevant effect measure |
| [278] | Udonsi et al., 1992 | No relevant effect measure |
| [279] | Ulukanligil et al., 2003 | No relevant effect measure |
| [280] | Ulukanligil et al., 2008 | No relevant effect measure |
| [281] | van Eijk et al., 2009 | No relevant effect measure |
| [282] | van Niekerk et al., 1979 | No relevant effect measure |
| [283] | Vanek et al., 1967 | No relevant effect measure |
| [284] | Vasconcelos et al., 2011 | No relevant effect measure |
| [285] | Verle et al., 2003 | No relevant effect measure |
| [286] | Wagbatsoma et al., 2008 | No relevant effect measure |
| [287] | Wang et al., 2012 | No relevant effect measure |
| [288] | Wani and Ahmad, 2009 | No relevant effect measure |
| [289] | Wani et al., 2007 | No relevant effect measure |
| [290] | Wani et al., 2008 | No relevant effect measure |
| [291] | Wani et al., 2010 | No relevant effect measure |
| [292] | Warunee, et al. 2007 | No relevant effect measure |
| [293] | Wen-Juan et al., 2011 | No relevant effect measure |
| [294] | White et al., 1986 | No relevant effect measure |
| [295] | Worrell et al., 2012 | No relevant effect measure |
| [296] | Xu et al., 2000 | No relevant effect measure |
| [297] | Yajima et al., 2008 | No relevant effect measure |
| [298] | Yami et al., 2011 | No relevant effect measure |
| [299] | Youmbi et al., 2013 | No relevant effect measure |
| [300] | Zeynudin et al., 2013 | No relevant effect measure |
| [301] | Zheng et al., 2009 | Review or opinion article |
| [302] | Ziegelbauer et al., 2012 | Review or opinion article |
| [303] | Zulu et al., 2011 | No relevant effect measure |
| [304] | Zulu et al., 2012 | No relevant effect measure |
| [305] | Zulu et al., 2013 | No relevant effect measure |

**References**

1. Aagaard-Hansen J, Nombela N, Alvar J (2010) Population movement: A key factor in the epidemiology of neglected tropical diseases. Trop Med Int Health 15: 1281-1288.

2. Abanyie FA, McCracken C, Kirwan P, Molloy SF, Asaolu SO, et al. (2013) *Ascaris* co-infection does not alter malaria-induced anaemia in a cohort of Nigerian preschool children. Malar J 12: 1.

3. Abera B, Alem G, Yimer M, Herrador Z (2013) Epidemiology of soil-transmitted helminths, *Schistosoma mansoni*, and haematocrit values among schoolchildren in Ethiopia. J Infect Dev Ctries 7: 253-260.

4. Abou-Zeid AHA, Abkar TA, Mohamed RO (2012) Schistosomiasis and soil-transmitted helminths among an adult population in a war affected area, Southern Kordofan state, Sudan. Parasit Vectors 5.

5. Abu Mourad TA (2004) Palestinian refugee conditions associated with intestinal parasites and diarrhoea: Nuseirat refugee camp as a case study. Public Health 118: 131-142.

6. Acka CA, Raso G, N'Goran EK, Tschannen AB, Bogoch II, et al. (2010) Parasitic worms: Knowledge, attitudes, and practices in western cote d'ivoire with implications for integrated control. PLoS Negl Trop Dis 4: 1-14.

7. Adams VJ, Markus MB, Adams JF, Jordaan E, Curtis B, et al. (2005) Paradoxical helminthiasis and giardiasis in Cape Town, South Africa: epidemiology and control. Afr Health Sci 5: 276-280.

8. Adeoye GO, Osayemi CO, Oteniya O, Onyemekeihia SO (2007) Epidemiological studies of intestinal helminthes and malaria among children in Lagos, Nigeria. Reprod Fertil Dev 19: 617-625.

9. Agbaya SS, Yavo W, Menan EI, Attey MA, Kouadio LP, et al. (2004) [Intestinal helminthiasis among school children: preliminary results of a prospective study in Agboville in Southern Cote d'Ivoire]. Sante 14: 143-147.

10. Agi PI (1995) Pattern of infection of intestinal parasites in Sagbama community of the Niger Delta, Nigeria. West Afr J Med 14: 39-42.

11. Agi PI (1997) Comparative helminth infections of man in two rural communities of the Niger Delta, Nigeria. West Afr J Med 16: 232-236.

12. Ahmed A, Shah R, Ahmed A (2004) De-worming campaign among Bangladeshi school children demands concomitant improvement of community sanitation status; MasComa S, editor. 357-362 p.

13. Akinbo FO, Okaka CE, Omoregie R (2010) Prevalence of intestinal parasitic infections among HIV patients in Benin City, Nigeria. Commun Agric Appl Biol Sci 75: 265-271.

14. Akinbo FO, Olujobi SO, Omoregie R, Egbe C (2013) Intestinal parasitic infections among diabetes mellitus patients. Biomarkers and Genomic Medicine 5: 44-47.

15. Alaofe H, Zee J, Dossa R, O'Brien HT (2008) Intestinal parasitic infections in adolescent girls from two boarding schools in southern Benin. Trans R Soc Trop Med Hyg 102: 653-661.

16. Alballaa SR, Alsekeit M, Alballa SR, Alrashed RS, Alhedaithy MA, et al. (1993) Prevalence of pathogenic intestinal parasites among preschool-children in Al-Medina district, Saudi-Arabia. Annals of Saudi Medicine 13: 259-263.

17. Al-Ballaa SR, Al-Sekeit M, Al-Balla SR, Al-Rashed RS, Al-Hedaithy MA, et al. (1993) Prevalence of pathogenic intestinal parasites among preschool children in Al-Medina district, Saudi Arabia. Vet Rec 132: 473-475.

18. Albright JW, Hidayati NR, Basaric-Keys J (2005) Behavioral and hygienic characteristics of primary schoolchildren which can be modified to reduce the prevalence of geohelminth infections: a study in central Java, Indonesia. Southeast Asian J Trop Med Public Health 36: 629-640.

19. Alemu A, Atnafu A, Addis Z, Shiferaw Y, Teklu T, et al. (2012) Soil transmitted helminths and schistosoma mansoni infections among school children in Zarima town, northwest Ethiopia. Parasitol Int 61: 101-106. doi: 110.1016/j.parint.2011.1006.1018. Epub 2011 Jun 1029.

20. Ali-Shtayeh MS, Hamdan AHY, Shaheen SF, Abu-Zeid I, Faidy YR (1989) Prevalence and seasonal fluctuations of intestinal parasitic infections in the Nablus area, West Bank of Jordan. Ann Trop Med Parasitol 83: 67-72.

21. Al-Khatib IA, Kamal S, Taha B, Al Hamad J, Jaber H (2003) Water - health relationships in developing countries: A case study in Tulkarem district in Palestine. International Journal of Environmental Health Research 13: 199-206.

22. Al-Madani AA, Omar MS, Abu-Zeid HA, Abdulla SA (1989) Intestinal parasites in urban and rural communities of Abha, Saudi Arabia. Annals of Saudi Medicine 9: 182-185.

23. Almeida R, Garrido C, Guedes M, Bravo L, Novoa C (2003) Intestinal helminthic infections. Nascer e Crescer 12: 15-17.

24. Al-Mohammed HI, Amin TT, Aboulmagd E, Hablus HR, Zaza BO (2010) Prevalence of intestinal parasitic infections and its relationship with socio-demographics and hygienic habits among male primary schoolchildren in Al-Ahsa, Saudi Arabia. Asian Pac J Trop Med 3: 906-912.

25. Nematian J, Nematian E, Gholamrezanezhad A, Asgari AA (2004) Prevalence of intestinal parasitic infections and their relation with socio-economic factors and hygienic habits in Tehran primary school students. Acta Trop 92: 179-186.

26. Carneiro FF, Cifuentes E, Tellez-Rojo MM, Romieu I (2002) The risk of Ascaris lumbricoides infection in children as an environmental health indicator to guide preventive activities in Caparao and Alto Caparao, Brazil. Rev Saude Publica 36: 69-74.

27. Alyousefi NA, Mahdy MA, Mahmud R, Lim YA (2011) Factors associated with high prevalence of intestinal protozoan infections among patients in Sana'a City, Yemen. PLoS ONE 6: e22044.

28. Alyousefi NA, Mahdy MAK, Mahmud R, Lim YAL (2011) Factors associated with high prevalence of intestinal protozoan infections among patients in Sana'a city, Yemen. PLoS ONE 6.

29. Amuta EU, Houmsou RS, Mker SD (2010) Knowledge and risk factors of intestinal parasitic infections among women in Makurdi, Benue State. Asian Pac J Trop Med 3: 993-996.

30. Anantaphruti MT, Nuamtanong S, Muennoo C, Sanguankiat S, Pubampen S (2000) Strongyloides stercoralis infection and chronological changes of other soil-transmitted helminthiases in an endemic area of southern Thailand. Southeast Asian J Trop Med Public Health 31: 383-387.

31. Anantaphruti MT, Waikagul J, Maipanich W, Nuamtanong S, Pubampen S (2004) Soil-transmitted helminthiases and health behaviors among schoolchildren and community members in a west-central border area of Thailand. Southeast Asian J Trop Med Public Health 35: 260-266.

32. Aramayo CF, Gil JF, Cruz MC, Poma HR, Last MS, et al. (2009) Diarrhea and parasitosis in Salta, Argentina. Journal of Infection in Developing Countries 3: 105-111.

33. Arfaa F, Sahba GH, Farahmandian I, Jalali H (1977) Evaluation of the effect of different methods of control of soil-transmitted helminths in Khuzestan, southwest Iran. Am J Trop Med Hyg 26: 230-233.

34. Arfaa F, Sahba GH, Farahmandian I, Jalali H (1977) Evaluation of the effect of different methods of control of soil-transmitted helminths in Khuzestan, southwest Iran. Am J Trop Med Hyg 26: 234-237.

35. Asaolu SO, Ofoezie IE (2003) The role of health education and sanitation in the control of helminth infections. Acta Trop 86: 283-294.

36. Atukorala TM, Lanerolle P (1999) Soil-transmitted helminthic infection and its effect on nutritional status of adolescent schoolgirls of low socioeconomic status in Sri Lanka. J Trop Pediatr 45: 18-22.

37. Ayanwale FO, Esuruoso GO (1982) The epidemiology of human intestinal helminthiasis in Ibadan, South Western Nigeria. Int J Zoonoses 9: 69-72.

38. Bagchi SC, Prasad BG, Mathur GB (1964) Study of some socio-environmental factors of hookworm infestation in area of rural health training centre, Sarojini Nagar, Lucknow. Indian J Med Res 52: 418-&.

39. Bailey C, Lopez S, Camero A, Taiquiri C, Arhuay Y, et al. (2013) Factors associated with parasitic infection amongst street children in orphanages across Lima, Peru. Pathogens and Global Health 107: 52-57.

40. Banbula E (1970) [Intestinal helminths in small children at Tarnow]. Bull Soc Pathol Exot Filiales 63: 79-89.

41. Banbula E (1971) [Infection of 3 to 7-year-old children with intestinal worms in 3 towns of the Dabrowa Tarnowska District (Cracow Province)]. Rocz Panstw Zakl Hig 22: 179-187.

42. Barrett DM, Steel-Duncan J, Christie CDC, Eldemire-Shearer D, Lindo JF (2008) Absence of opportunistic parasitic infestations in children living with HIV/AIDS in children's homes in Jamaica: Pilot investigations. West Indian Med J 57: 253-256.

43. Basualdo JA, Cordoba MA, de Luca MM, Ciarmela ML, Pezzani BC, et al. (2007) Intestinal parasitoses and environmental factors in a rural population of Argentina, 2002-2003. Rev Inst Med Trop Sao Paulo 49: 251-255.

44. Baxter E, Rose PD, Kirby R (1994) Age and population group related distribution of enteropathogens in the Eastern Cape, South Africa. Lett Appl Microbiol 19: 442-445.

45. Behnke JM, De Clercq D, Sacko M, Gilbert FS, Ouattara DB, et al. (2000) The epidemiology of human hookworm infections in the southern region of Mali. Trop Med Int Health 5: 343-354.

46. Belo VS, de Oliveira RB, Fernandes PC, Nascimento BWL, Fernandes FV, et al. (2012) Factors associated with intestinal parasitosis in a population of children and adolescents. Revista Paulista de Pediatria 30: 195-201.

47. Bethony J, Chen J, Lin S, Xiao S, Zhan B, et al. (2002) Emerging patterns of hookworm infection: influence of aging on the intensity of Necator infection in Hainan Province, People's Republic of China. Trends Cell Biol 12: 479-484.

48. Bhandari N, Kausaph V, Neupane GP (2011) Intestinal parasitic infection among school age children. Environ Microbiol 13: 1590-1600. doi: 1510.1111/j.1462-2920.2011.02470.x. Epub 02011 Mar 02430.

49. Bidinger PD, Crompton DW, Arnold S (1981) Aspects of intestinal parasitism in villagers from rural peninsular India. Parasitology 83: 401-409.

50. Bisht D, Verma AK, Bharadwaj HH (2011) Intestinal parasitic infestation among children in a semi-urban Indian population. Trans R Soc Trop Med Hyg 105: 500-506. doi: 510.1016/j.trstmh.2011.1005.1007. Epub 2011 Jun 1028.

51. Bisi-Johnson MA, Obi CL, Ekosse GE (2010) Microbiological and health related perspectives of geophagia: An overview. African Journal of Biotechnology 9: 5784-5791.

52. Blumenthal UJ, Mara DD, Ayres RM, Cifuentes E, Peasey A, et al. (1996) Evaluation of the who nematode egg guidelines for restricted and unrestricted irrigation. Water Sci Technol 33: 277-283.

53. Blumenthal UJ, Cifuentes E, Bennett S, Quigley M, Ruiz-Palacios G (2001) The risk of enteric infections associated with wastewater reuse: the effect of season and degree of storage of wastewater. Trans R Soc Trop Med Hyg 95: 131-137.

54. Boccaccio M (1972) Ground itch and dew poison; the Rockefeller Sanitary Commission 1909-14. J Hist Med Allied Sci 27: 30-53.

55. Boia MN, da Motta LP, Salazar MD, Mutis MP, Coutinho RB, et al. (1999) [Cross-sectional study of intestinal parasites and Chagas' disease in the Municipality of Novo Airao, State of Amazonas, Brazil]. Exp Parasitol 93: 66-72.

56. Borda CE, Felisa Rea MJ, Rosa JR, Maidana C (1996) Intestinal parasitism in San Cayetano, Corientes, Argentina. Bol Oficina Sanit Panam 120: 110-116.

57. Bosman A, De Giorgi F, Kandia Diallo I, Pizzi L, Bartoloni P, et al. (1991) Prevalence and intensity of infection with intestinal parasites in areas of the Futa Djalon, Republic of Guinea. Parassitologia 33: 203-208.

58. Bouchet F, Leger N (1986) Parasitic pollution of sewage sludges in Champagne area. Bulletin de la Societe Francaise de Parasitologie 4: 13-18.

59. Bouhoum K, Amahmid O (2000) Health effect of wastewater reuse in agriculture. Schriftenr Ver Wasser Boden Lufthyg 105: 249-256.

60. Bouree P, David P, Basset D, Coco O, Beauvais B, et al. (1984) [Epidemiologic survey of intestinal parasitoses in Peruvian Amazonia]. Ann Rech Vet 15: 519-528.

61. Bradley M, Chandiwana SK, Bundy DA (1993) The epidemiology and control of hookworm infection in the Burma Valley area of Zimbabwe. Trans R Soc Trop Med Hyg 87: 148-152.

62. Branco N, Leal DAG, Franco RMB (2012) A parasitological survey of natural water springs and inhabitants of a tourist city in Southeastern Brazil. Vector Borne Zoonotic Dis 12: 410-417.

63. Breitling LPH, Wilson AJ, Raiko A, Lagog M, Siba P, et al. (2008) Heritability of human hookworm infection in Papua New Guinea. Parasitology 135: 1407-1415.

64. Brocklehurst C, Bartram J (2010) Swimming upstream: Why sanitation, hygiene and water are so important to mothers and their daughters. Bull World Health Organ 88: 482.

65. Brown J, Cairncross S, Ensink JHJ (2013) Water, sanitation, hygiene and enteric infections in children. Arch Dis Child 98: 629-634.

66. Buck AA, Anderson RI, MacRae AA, Fain A (1978) Epidemiology of poly-parasitism. I. Occurrence, frequency and distribution of multiple infections in rural communities in Chad, Peru, Afghanistan, and Zaire. Tropenmed Parasitol 29: 61-70.

67. Bunnag T, Sornmani S, Impand P, Harinasuta C (1980) Potential health hazards of the water resources development: a health survey in the Phitsanulok Irrigation Project, Nan River Basin, Northern Thailand. Indian J Med Sci 34: 308-309.

68. Cai L, Huang DS, Ma XB, Zhang BX, Fu YH, et al. (2002) [Prevention and control of intestinal nematode infection in Shanghai]. Zhongguo Ji Sheng Chong Xue Yu Ji Sheng Chong Bing Za Zhi 20: 82.

69. Cancrini G, Bartoloni A, Nunez L, Paradisi F (1988) Intestinal parasites in the Camiri, Gutierrez and Boyuibe areas, Santa Cruz Department, Bolivia. Am J Trop Med Hyg 38: 568-573.

70. Cancrini G, Bartoloni A, Paradisi F, Nunez LE (1989) Parasitological observations on three Bolivian localities including rural communities, cities and institutions. Ann Trop Med Parasitol 83: 591-594.

71. Canete R, Diaz MM, Avalos Garcia R, Laud Martinez PM, Manuel Ponce F (2012) Intestinal Parasites in Children from a Day Care Centre in Matanzas City, Cuba. PLoS ONE 7.

72. Carlton EJ, Liang S, McDowell JZ, Li H, Luo W, et al. (2012) Regional disparities in the burden of disease attributable to unsafe water and poor sanitation in China. Bull World Health Organ 90: 578-587.

73. Carvalho P, Teodosio R (2011) Intestinal parasitic infection among schoolchildren in Fogo Island, Cape Verde. Trop Med Int Health 16: 195.

74. Chandiwana SK, Bradley M, Chombo F (1989) Hookworm and roundworm infections in farm-worker communities in the large-scale agricultural sector in Zimbabwe. Vet Parasitol 33: 231-239.

75. Chandler AC (1954) A comparison of helminthic and protozoan infections in two Egyptian villages two years after the installation of sanitary improvements in one of them. Bull World Health Organ 10: 229-249.

76. Chaskar D, Kaskhedikar P, Chaskar R, Kaskhedikar TA (1996) Epidemiological survey of gastrointestinal nematode infection in certain endemic regions of Indore city. Journal of Environmental Biology 17: 345-347.

77. Chirdan OO, Akosu JT, Adah SO (2010) Intestinal parasites in children attending day care centers in Jos, Central Nigeria. Niger J Med 19: 219-222.

78. Chunge RN, Simwa JM, Karumba PN, Kenya PR, Kinoti SN, et al. (1992) Comparative aetiology of childhood diarrhoea in Kakamega and Kiambu Districts, Kenya. Int J Parasitol 22: 563-572.

79. Cifuentes E, Blumenthal U, Ruizpalacios G, Bennett S, Peasey A (1994) Epidemiologic panorama for the agricultural use of waste-water - the Mezquital Valley, Mexico. Salud Publica Mex 36: 3-9.

80. Cifuentes E (1998) The epidemiology of enteric infections in agricultural communities exposed to wastewater irrigation: perspectives for risk control. International Journal of Environmental Health Research 8: 203-213.

81. Clark C (2006) Parasitic worm infestations. Pharmaceutical Journal 277: 343-346.

82. Clasen T, Boisson S, Routray P, Cumming O, Jenkins M, et al. (2012) The effect of improved rural sanitation on diarrhoea and helminth infection: Design of a cluster-randomized trial in Orissa, India. Emerging Themes in Epidemiology: 7.

83. Clennon JA, Huttinger AD, Smith ER, Moe CL (2010) Spatial patterning of health disparities and environmental factors associated with Ascaris lumbricoides prevalence in Bolivia. Am J Trop Med Hyg 83: 354-354.

84. Cremades Romero MJ, Igual Adell R, Ricart Olmos C, Estelles Piera F, Pastor-Guzman A, et al. (1997) [Infection by Strongyloides stercoralis in the county of Safor, Spain]. Vet Rec 141: 56.

85. Cruz A, Cabral M, Sousa MI, Azeredo Z (2002) Intestinal parasitoses. Arquivos de Medicina 16: 211-218.

86. Curtale F, Pezzotti P, Sharbini AL, Al Maadat H, Ingrosso P, et al. (1998) Knowledge, perceptions and behaviour of mothers toward intestinal helminths in Upper Egypt: Implications for control. Health Policy Plan 13: 423-432.

87. Curtale F, Pezzotti P, Saad YS, Aloi A (1999) An analysis of individual, household, and environmental risk factors for intestinal helminth infection among children in Qena Governorate, Upper Egypt. J Trop Pediatr 45: 18-22.

88. Dancesco P, Abeu J, Akakpo C, Iamandi I, Kacou E, et al. (2005) [Intestinal parasitoses in a village of Cote d'Ivoire. I: Control and prevention plan]. Sante 15: 5-10.

89. Daryani A, Sharif M, Nasrolahei M, Khalilian A, Mohammadi A, et al. (2012) Epidemiological survey of the prevalence of intestinal parasites among schoolchildren in Sari, northern Iran. Trans R Soc Trop Med Hyg 106: 455-459.

90. de Silva NR, Jayapani VP, de Silva HJ (1996) Socioeconomic and behavioral factors affecting the prevalence of geohelminths in preschool children. Southeast Asian J Trop Med Public Health 27: 43-46.

91. Dumba R, Kaddu JB, Mangen FW (2008) Intestinal helminths in Luweero district, Uganda. Afr Health Sci 8: 90-96.

92. Dunn FL (1972) Intestinal parasitism in Malayan aborigines (Orang Asli). Bull World Health Organ 46: 99-113.

93. Ekpo UF, Odoemene SN, Mafiana CF, Sam-Wobo SO (2008) Helminthiasis and hygiene conditions of schools in Ikenne, Ogun State, Nigeria. Vet Res Commun 32: 383-391. doi: 310.1007/s11259-11008-19046-x. Epub 12008 Mar 11221.

94. El Kettani S, Azzouzi E, Boukachabine K, El Yamani M, Maata A, et al. (2008) Intestinal parasitosis and use of untreated wastewater for agriculture in Settat, Morocco. East Mediterr Health J 14: 1435-1444.

95. Elmadhoun WMY, Msmar AHH, Elnobyc OAE, Noor SKM, Suliman AA, et al. (2013) Situation analysis of schistosomiasis and soil-transmitted helminthes in River Nile State, Sudan. Trans R Soc Trop Med Hyg 107: 195-199.

96. Embil JA, Pereira LH, White FM, Garner JB, Manuel FR (1984) Prevalence of *Ascaris lumbricoides* infection in a small Nova Scotian community. Am J Trop Med Hyg 33: 595-598.

97. Ensink JH, van der Hoek W, Mukhtar M, Tahir Z, Amerasinghe FP (2005) High risk of hookworm infection among wastewater farmers in Pakistan. Trans R Soc Trop Med Hyg 99: 809-818.

98. Ensink JHJ, Blumenthal UJ, Brooker S (2008) Wastewater quality and the risk of intestinal nematode infection in sewage farming families in Hyderabad, India. Am J Trop Med Hyg 79: 561-567.

99. Erko B, Tedla S (1993) A preliminary survey for intestinal parasites in the Tis Abay town, northwest Ethiopia, with special references to Schistosoma mansoni. East Afr Med J 70: 34-36.

100. Escobedo AA, Canete R, Nunez FA (2008) Prevalence, risk factors and clinical features associated with intestinal parasitic infections in children from San Juan y Martinez, Pinar del Rio, Cuba. West Indian Med J 57: 377-382.

101. Espinoza F, Tanowitz H, Andrews P, Gendlina I, Ruddock JS, et al. (2012) Screening for strongyloides infection in an immigrant population in bronx, New york. Am J Trop Med Hyg 87: 146.

102. Esrey SA, Potash JB, Roberts L, Shiff C (1991) Effects of improved water supply and sanitation on ascariasis, diarrhoea, dracunculiasis, hookworm infection, schistosomiasis, and trachoma. Bull World Health Organ 69: 609-621.

103. Ezeagwuna DA, Okwelogu IS, Ekejindu IM, Ogbuagu CN (2010) The prevalence and socio-economic factors of intestinal helminth infections among primary school pupils in Ozubulu, Anambra State, Nigeria. Internet Journal of Epidemiology 9.

104. Fashuyi SA (1988) An observation of the dynamics of intestinal helminth infections in two isolated communities in south-western Nigeria. Med Parazitol (Mosk): 64-67.

105. Fatiregun AA, Oluwatoba OA (2008) Pattern of intestinal helminth infections among school children in an urban community in Ibadan, Nigeria. Asian Pac J Trop Med 1: 42-47.

106. Fattal B, Guttman-Bass N, Agursky T, Shuval HI (1988) Evaluation of health risk associated with drinking water quality in agricultural communities. Water Sci Technol 20: 409-415.

107. Ferrell JA (1920) Role of the latrine in control of hookworm disease. Am J Public Health 10: 138-140.

108. Flohr C, Tuyen LN, Lewis S, Quinnell R, Minh TT, et al. (2006) Poor sanitation and helminth infection protect against skin sensitization in Vietnamese children: A cross-sectional study. J Allergy Clin Immunol 118: 1305-1311.

109. Fort AG (1915) Rural sanitation and hookworm disease. Am J Public Health (N Y) 5: 1038-1043.

110. Fouamno Kamga HL, Shey Nsagha D, Suh Atanga MB, Longdoh Njunda A, Nguedia Assob JC, et al. (2011) The impact of health education on the prevalence of faecal-orally transmitted parasitic infections among school children in a rural community in Cameroon. Pan Afr Med J 8: 38.

111. Fuentes M, Galindez L, Garcia D, Gonzalez N, Goyanes J, et al. (2011) Frequency of Intestinal Parasitism and Epidemiological Characteristics of the 1 to 12 Year-Old Child Population Treated at the Cerro Gordo Type II Urban Outpatient Clinic. Barquisimeto, State of Lara. January-June 2007. Kasmera 39: 31-42.

112. Fung IC, Cairncross S (2009) Ascariasis and handwashing. Trans R Soc Trop Med Hyg 103: 215-222.

113. Gamboa MI, Basualdo JA, Kozubsky L, Costas E, Cueto Rua E, et al. (1998) Prevalence of intestinal parasitosis within three population groups in La Plata, Argentina. Eur J Epidemiol 14: 55-61.

114. Gamboa MI, Basualdo JA, Cordoba MA, Pezzani BC, Minvielle MC, et al. (2003) Distribution of intestinal parasitoses in relation to environmental and sociocultural parameters in La Plata, Argentina. J Helminthol 77: 33-38.

115. Gamboa MI, Kozubsky LE, Costas ME, Garraza M, Cardozo MI, et al. (2009) [Associations between geohelminths and socioenvironmental conditions among different human populations in Argentina]. Rev Panam Salud Publica 26: 1-8.

116. Gamboa MI, Navone GT, Orden AB, Torres MF, Castro LE, et al. (2011) Socio-environmental conditions, intestinal parasitic infections and nutritional status in children from a suburban neighborhood of La Plata, Argentina. Acta Trop 118: 184-189.

117. Gamboa MI, Navone GT, Zonta ML (2012) The prevalence of geohelminthiasis is related to socio-environmental conditions. Salud(i)Ciencia 19: 16-21.

118. Garg BS, Singh JV, Gupta SC (1984) Impact of physical environment on the prevalene of intestinal helminthic infestation in under five children. Indian J Med Sci 38: 88-90.

119. Gelaw A, Anagaw B, Nigussie B, Silesh B, Yirga A, et al. (2013) Prevalence of intestinal parasitic infections and risk factors among schoolchildren at the University of Gondar Community School, Northwest Ethiopia: a cross-sectional study. BMC Public Health 13.

120. Getachew M, Tafess K, Zeynudin A, Yewhalaw D (2013) Prevalence soil transmitted helminthiasis and malaria co-infection among pregnant women and risk factors in Gilgel Gibe Dam area, southwest Ethiopia. BMC Res Notes 6:263.: 10.1186/1756-0500-1186-1263.

121. Gross R, Schell B, Molina MC, Leao MA, Strack U (1989) The impact of improvement of water supply and sanitation facilities on diarrhea and intestinal parasites: a Brazilian experience with children in two low-income urban communities. J Parasitol 75: 461-463.

122. Gryseels B, Gigase PL (1985) The prevalence of intestinal parasites in two suburbs of Kinshasa (Zaire) and their relation to domestic water supplies. Ann Soc Belg Med Trop 65: 173-177.

123. Gumbo JR, Malaka EM, Odiyo JO, Nare L (2010) The health implications of wastewater reuse in vegetable irrigation: a case study from Malamulele, South Africa. Int J Environ Health Res 20: 201-211.

124. Gungoren B, Latipov R, Regallet G, Musabaev E (2007) Effect of hygiene promotion on the risk of reinfection rate of intestinal parasites in children in rural Uzbekistan. Trans R Soc Trop Med Hyg 101: 564-569.

125. Gyawali N, Amatya R, Nepal HP (2009) Intestinal parasitosis in school going children of Dharan municipality, Nepal. Trop Gastroenterol 30: 145-147.

126. Gyorkos TW, Camara B, Kokoskin E, Carabin H, Prouty R (1996) A parasite prevalence survey in school-aged children in Guinea (1995). Cahiers Sante 6: 377-381.

127. Gyoten J, Hoa NTV, Fujimaki Y, Tanaka K, Uga S, et al. (2010) The correlation between contamination of soil with Ascaris sp. eggs in school yards and ascariasis among primary school children in Mai trung commune, Northern Vietnam. Tropical Medicine and Health 38: 35-38.

128. Habbari K, Tifnouti A, Bitton G, Mandil A (1999) Helminthic infections associated with the use of raw wastewater for agricultural purposes in Beni Mellal, Morocco. Eastern Mediterranean health journal = La revue de santé de la Méditerranée orientale = al-Majallah al-ihhīyah li-sharq al-mutawassi 5: 912-921.

129. Habbari K, Tifnouti A, Bitton G, Mandil A (2000) Geohelminthic infections associated with raw wastewater reuse for agricultural purposes in Beni-Mellal, Morocco. Parasitol Int 48: 249-254.

130. Hagel I, Lynch NR, Perez M, Di Prisco MC, Lopez R, et al. (1993) Relationship between the degree of poverty and the IgE response to Ascaris infection in slum children. Trans R Soc Trop Med Hyg 87: 16-18.

131. Handzel T, Karanja DM, Addiss DG, Hightower AW, Rosen DH, et al. (2003) Geographic distribution of schistosomiasis and soil-transmitted helminths in Western Kenya: implications for anthelminthic mass treatment. Am J Trop Med Hyg 69: 318-323.

132. Herrera J, Marcos L, Terashima A, Alvarez H, Samalvides F, et al. (2006) [Factors associated with strongyloides stercoralis infection in an endemic area in Peru]. Rev Gastroenterol Peru 26: 357-362.

133. Heukelbach J, Jackson A, Ariza L, Feldmeier H (2008) Prevalence and risk factors of hookworm-related cutaneous larva migrans in a rural community in Brazil. Trans R Soc Trop Med Hyg 102: 297-298; author reply 298. doi: 210.1016/j.trstmh.2007.1011.1007. Epub 2008 Jan 1019.

134. Holland CV, Taren DL, Crompton DW, Nesheim MC, Sanjur D, et al. (1988) Intestinal helminthiases in relation to the socioeconomic environment of Panamanian children. Soc Sci Med 26: 209-213.

135. Hosain GM, Saha S, Begum A (2003) Impact of sanitation and health education on intestinal parasite infection among primary school aged children of Sherpur, Bangladesh. Trop Doct 33: 163-164.

136. Humphries DL, Stephenson LS, Pearce EJ, The PH, Dan HT, et al. (1997) The use of human faeces for fertilizer is associated with increased intensity of hookworm infection in Vietnamese women. Trans R Soc Trop Med Hyg 91: 518-520.

137. Le Hung Q, de Vries PJ, Giao PT, Binh TQ, Nam NV, et al. (2005) Intestinal helminth infection in an ethnic minority commune in southern Vietnam. Southeast Asian J Trop Med Public Health 36: 629-640.

138. Hunt C (2001) A review of the health hazards associated with the occupation of waste picking for children. Int J Adolesc Med Health 13: 177-189.

139. Huruy K, Kassu A, Mulu A, Worku N, Fetene T, et al. (2011) Intestinal parasitosis and shigellosis among diarrheal patients in Gondar teaching hospital, northwest Ethiopia. BMC Res Notes 4: 472.

140. Hussein AS (2011) Prevalence of intestinal parasites among school children in northern districts of West Bank-Palestine. Trop Med Int Health 16: 240-244.

141. Huttly SRA (1990) The impact of inadequate sanitary conditions on health in developing countries. World Health Stat Q 43: 118-126.

142. Idowu OA, Rowland SA (2006) Oral fecal parasites and personal hygiene of food handlers in Abeokuta, Nigeria. Afr Health Sci 6: 160-164.

143. Idowu OA, Babatunde O, Soniran T, Adediran A (2011) Parasitic infections in finger-sucking school age children. Pediatr Infect Dis J 30: 791-792.

144. Ikeh EI, Obe E, Kidmas AT (2011) Screening for intestinal parasites in elective surgery patients in endemic areas: How relevant is it? Laboratory Medicine 42: 469-472.

145. Ilechukwu GC, Ilechukwu CG, Ozumba AN, Ojinnaka NC, Ibe BC, et al. (2011) Some behavioural risk factors for intestinal helminthiasis in nursery and primary school children in Enugu, south eastern Nigeria. Water Res 45: 665-673. doi: 610.1016/j.watres.2010.1008.1028. Epub 2010 Aug 1021.

146. Ishiyama S, Rai SK, Ono K, Uga S (2006) Small-scale study on intestinal parasitosis in a remote hilly village in Nepal. Parasitol Int 55: 163-167. Epub 2006 Mar 2006.

147. Jacobsen KH, Ribeiro PS, Quist BK, Rydbeck BV (2007) Prevalence of intestinal parasites in young Quichua children in the highlands of rural Ecuador. J Health Popul Nutr 25: 399-405.

148. Jombo GT, Egah DZ, Akosu JT (2007) Intestinal parasitism, potable water availability and methods of sewage disposal in three communities in Benue State, Nigeria: a survey. Ann Afr Med 6: 17-21.

149. Jombo GT, Damen JG, Safiyanu H, Odey F, Mbaawuaga EM (2010) Human intestinal parasitism, potable water availability and methods of sewage disposal among nomadic Fulanis in Kuraje rural settlement of Zamfara state. Asian Pac J Trop Med 3: 491-493.

150. Kaminsky RG (1991) Parasitism and diarrhoea in children from two rural communities and marginal barrio in Honduras. Trans R Soc Trop Med Hyg 85: 70-73.

151. Karaman U, Atambay M, Aycan O, Yologlu S, Daldal N (2006) [Incidence of intestinal parasites in municipal sanitary workers in Malatya]. Turkiye Parazitol Derg 30: 190-193.

152. Karan A, Chapman GB, Galvani A (2012) The influence of poverty and culture on the transmission of parasitic infections in rural Nicaraguan villages. Journal of Parasitology Research 2012.

153. Kawai K, Saathoff E, Antelman G, Msamanga G, Fawzi WW (2009) Geophagy (soil-eating) in relation to anemia and helminth infection among HIV-infected pregnant women in Tanzania. Am J Trop Med Hyg 80: 36-43.

154. El Kettani S, Azzouzi el M (2006) [Prevalence of helminths in a rural population using wastewater for agricultural purposes at Settat (Morocco)]. Sante 16: 245-251.

155. Kightlinger LK, Seed JR, Kightlinger MB (1998) *Ascaris lumbricoides* intensity in relation to environmental, socioeconomic, and behavioral determinants of exposure to infection in children from southeast Madagascar. J Parasitol 84: 480-484.

156. King JD, Endeshaw T, Escher E, Alemtaye G, Melaku S, et al. (2013) Intestinal parasite prevalence in an area of ethiopia after implementing the SAFE strategy, enhanced outreach services, and health extension program. PLoS Negl Trop Dis 7: e2223. doi: 2210.1371/journal.pntd.0002223. Print 0002013.

157. Kipyegen CK, Shivairo RS, Odhiambo RO (2012) Prevalence of intestinal parasites among HIV patients in Baringo, Kenya. Pan Afr Med J 13: 37. Epub 2012 Oct 2021.

158. Kitvatanachai S, Boonsilp S, Watanasatitarpa S (2008) Intestinal parasitic infections in Srimum suburban area of Nakhon Ratchasima Province, Thailand. Tropical Biomedicine 25: 237-242.

159. Kloos H, DeSole G, Lemma A (1981) Intestinal parasitism in seminomadic pastoralists and subsistence farmers in and around irrigation schemes in the Awash Valley, Ethiopia, with special emphasis on ecological and cultural associations. Social Science and Medicine - Part B Medical Anthropology 15: 457-469.

160. Koga-Kita K (2004) Intestinal parasitic infections and socioeconomic status in Prek Russey Commune, Cambodia. Parasite Immunol 26: 327-333.

161. Komiya Y, Kunii C (1964) The epidemiology of *ascaris* infection in relation to its control program in Japan. Jpn J Med Sci Biol 17: 23-31.

162. Kosoff P, Hernandez F, Pardo V, Visconti M, Zimmerman M (1989) Urban helminthiasis in two socioeconomically distinct Costa Rican communities. Science 246: 1015-1022.

163. Krause RJ, Koski KG, Scott ME (2012) Evidence that multisector food security intervention program in rural panama reduces hookworm infection in preschool children. Am J Trop Med Hyg 87: 341.

164. Kroeger A, Schulz S, Witte B, Skewes-Ramm R, Etzler A (1992) Helminthiasis and cultural change in the Peruvian rainforest. J Trop Med Hyg 95: 95-103.

165. Kurniawan A (2012) Intestinal parasitic infections among primary school children in north Jakarta: Dominance of soil transmitted helminths and blastocystis infections. Am J Trop Med Hyg 87: 37.

166. Lansdown R, Ledward A, Hall A, Issae W, Yona E, et al. (2002) Schistosomiasis, helminth infection and health education in Tanzania: achieving behaviour change in primary schools. Health Educ Res 17: 425-433.

167. Lewin S, Norman R, Nannan N, Bradshaw D, Thomas E (2007) Estimating the burden of disease attributable to unsafe water and lack of sanitation and hygiene in South Africa in 2000. S Afr Med J 97: 755-762.

168. Lin CJ, Katongole-Mbidde E, Byekwaso T, Orem J, Rabkin CS, et al. (2008) Intestinal parasites in Kaposi sarcoma patients in Uganda: indication of shared risk factors or etiologic association. Am J Trop Med Hyg 78: 409-412.

169. Ilechukwu GC, Ilechukwu CGA, Ozumba AN, Ojinnaka NC, Ibe BC, et al. (2010) Some behavioural risk factors for intestinal helminthiasis in nursery and primary school children in Enugu, south eastern Nigeria. Nigerian Journal of Clinical Practice 13: 288-293.

170. Lura MC, Beltramino DM, de Carrera EF (2002) [Prevalence of intestinal helminthiasis in primary school children in Santa Fe city]. Trop Anim Health Prod 34: 115-120.

171. Machado RC, Marcari EL, de Cristante S, Crisante V, Carareto CM (1999) Giardiasis and helminthiasis in children of both public and private day-care centers and junior and high schools in the city of Mirassol, Sao Paulo State, Brazil. Rev Soc Bras Med Trop 32: 697-704.

172. Soares Magalhaes RJ, Barnett AG, Clements AC (2011) Geographical analysis of the role of water supply and sanitation in the risk of helminth infections of children in West Africa. Proc Natl Acad Sci U S A 108: 20084-20089.

173. Olsen A, Magnussen P, Ouma JH, Andreassen J, Friis H (1998) The contribution of hookworm and other parasitic infections to haemoglobin and iron status among children and adults in western Kenya. Trans R Soc Trop Med Hyg 92: 643-649.

174. Mahfouz AAR, El-Morshedy H, Farghaly A, Khalil A (1997) Ecological determinants of intestinal parasitic infections among pre-school children in an urban squatter settlement of Egypt. J Trop Pediatr 43: 341-344.

175. Maia MM, Fausto MA, Vieira EL, Benetton ML, Carneiro M (2009) Intestinal parasitic infection and associated risk factors, among children presenting at outpatient clinics in Manaus, Amazonas state, Brazil. Ann Trop Med Parasitol 103: 583-591.

176. Mamunur Rashid AKM, Saifur Rashid AKM, Rahman A (2011) Prevalence of intestinal parasitoses in urban and rural children of a developing country. Asian Pacific Journal of Tropical Biomedicine 1: S268-S270.

177. Mangali A, Sasabone P, Syafruddin, Abadi K, Hasegawa H, et al. (1993) Intestinal parasitic infections in Campalagian district, south Sulawesi, Indonesia. Southeast Asian J Trop Med Public Health 24: 321-326.

178. Mangali A, Sasabone P, Syafruddin, Abadi K, Hasegawa H, et al. (1994) Prevalence of intestinal helminthic infections in Kao District, north Halmahera, Indonesia. Southeast Asian J Trop Med Public Health 25: 745-751.

179. Mara D, Sleigh A (2010) Estimation of Ascaris infection risks in children under 15 from the consumption of wastewater-irrigated carrots. J Water Health 8: 35-38.

180. Mascarini-Serra L (2012) Prevention of Soil-transmitted Helminth Infection. Parasitol Res 110: 449-458. doi: 410.1007/s00436-00011-02511-00434. Epub 02011 Jul 00436.

181. Matthys B, Bobieva M, Karimova G, Mengliboeva Z, Jean-Richard V, et al. (2011) Prevalence and risk factors of helminths and intestinal protozoa infections among children from primary schools in western Tajikistan. Parasit Vectors 4: 195.

182. Mboera LEG, Senkoro KP, Rumisha SF, Mayala BK, Shayo EH, et al. (2011) Plasmodium falciparum and helminth coinfections among schoolchildren in relation to agro-ecosystems in Mvomero District, Tanzania. Acta Trop 120: 95-102.

183. McMullen PD, Aprison EZ, Winter PB, Amaral LA, Morimoto RI, et al. (2012) Effect of sanitation on soil-transmitted helminth infection: systematic review and meta-analysis. PLoS Comput Biol 8: e1002338. doi: 1002310.1001371/journal.pcbi.1002338. Epub 1002012 Jan 1002326.

184. Menan EI, Nebavi NG, Adjetey TA, Assavo NN, Deddy BA, et al. (1997) Influence of socioeconomic conditions on the occurrence of intestinal helminthiases. Study of 1001 students in Abidjan (Ivory Coast). Santé (Montrouge, France) 7: 205-209.

185. Midzi N, Mtapuri-Zinyowera S, Mapingure MP, Paul NH, Sangweme D, et al. (2011) Knowledge attitudes and practices of grade three primary schoolchildren in relation to schistosomiasis, soil transmitted helminthiasis and malaria in Zimbabwe. BMC Infect Dis 11: 169.

186. Mil R, Fernandez C, Goncnullalves AQ, Abellana R, Perez T, et al. (2011) Influence of intestinal parasitism and environmental conditions on childhood nutritional status in a periurban and rural area in La Paz, Bolivia. Trop Med Int Health 16: 254.

187. Minamoto K, Mascie-Taylor CG, Karim E, Moji K, Rahman M (2012) Short- and long-term impact of health education in improving water supply, sanitation and knowledge about intestinal helminths in rural Bangladesh. Public Health 126: 437-440.

188. Missaye A, Dagnew M, Alemu A, Alemu A (2013) Prevalence of intestinal parasites and associated risk factors among HIV/AIDS patients with pre-ART and on-ART attending dessie hospital ART clinic, Northeast Ethiopia. AIDS Res Ther 10.

189. Monse B, Benzian H, Naliponguit E, Belizario V, Schratz A, et al. (2013) The Fit for School health outcome study - a longitudinal survey to assess health impacts of an integrated school health programme in the Philippines. BMC Public Health 13.

190. Moore HA, De la Cruz E, Vargas-Mendez O (1965) Diarrheal disease studies in Costa Rica. IV. The influence of sanitation upon the prevalence of intestinal infection and diarrheal disease. Clin Pediatr (Phila) 4: 515-522.

191. Mora L, Segura M, Martinez I, Figuera L, Salazar S, et al. (2009) Intestinal Parasitism and Associated Sanitary-Hygienic Factors In Individuals of Rural Localities in Sucre State. Kasmera 37: 148-156.

192. Mordi RM, Okaka CE (2009) Prevalence of intestinal parasites in Edo State. International Journal of Health Research 2: 253-257.

193. Moubarrad FZL, Assobhei O (2005) The health effects of wastewater on the prevalence of ascariasis among the children of the discharge zone of El Jadida, Morocco. International Journal of Environmental Health Research 15: 135-142.

194. Moulinier C, Battin J, Giap G (1990) [Development of the prevalence rate of four intestinal parasites in children]. Acta Clin Belg 45: 227-239.

195. Muller M, Sanchez RM, Suswillo RR (1989) Evaluation of a sanitation programme using eggs of Ascaris lumbricoides in household yard soils as indicators. Vet Rec 124: 111-114.

196. Nasr NA, Al-Mekhlafi HM, Ahmed A, Roslan MA, Bulgiba A (2013) Towards an effective control programme of soil-transmitted helminth infections among Orang Asli in rural Malaysia. Part 2: Knowledge, attitude, and practices. Parasit Vectors 6: 28.

197. Navarrete N, Torres P (1994) [Prevalence of infection by intestinal helminths and protozoa in school children from a coastal locality in the province of Valdivia, Chile]. Med Parazitol (Mosk): 24-27.

198. Ngui R, Ishak S, Chuen CS, Mahmud R, Lim YA (2011) Prevalence and risk factors of intestinal parasitism in rural and remote West Malaysia. PLoS Negl Trop Dis 5: e974.

199. Nitulescu V (1954) [Anti-helminth preventive measures for use in rural areas]. Am J Trop Med Hyg 3: 764-772.

200. Nkenfou CN, Nana CT, Payne VK (2013) Intestinal parasitic infections in HIV infected and non-infected patients in a low HIV prevalence region, West-Cameroon. PLoS ONE 8: e57914. doi: 57910.51371/journal.pone.0057914. Epub 0052013 Feb 0057925.

201. Oberg C, Biolley MA, Duran V, Matamala R, Oxs E (1993) Intestinal parasites in the riverside population of Villarrica Lake, Chile. Bol Chil Parasitol 48: 8-11.

202. Obiamiwe BA (1977) The pattern of parasitic infection in human gut at the Specialist Hospital, Benin City, Nigeria. Br Heart J 39: 238-241.

203. Okyay P, Ertug S, Gultekin B, Onen O, Beser E (2004) Intestinal parasites prevalence and related factors in school children, a western city sample-Turkey. BMC Public Health 4.

204. Opara AA (2005) Water supplies in some rural communities around Calabar, Cross River State, Nigeria: impact on water-related diseases. Nepal Med Coll J 7: 43-46.

205. Ozturk CE, Sahin I, Yavuz T, Ozturk A, Akgunoglu M, et al. (2004) Intestinal parasitic infection in children in post-disaster situations years after earthquake. Pediatr Int 46: 656-662.

206. Ozumba UC, Ozumba NA, Anya S (2005) Helminthiasis in pregnancy in Enugu, Nigeria. Journal of Health Science 51: 291-293.

207. Pegelow K, Gross R, Pietrzik K, Lukito W, Richards AL, et al. (1997) Parasitological and nutritional situation of school children in the Sukaraja district, West Java, Indonesia. Trop Med Int Health 2: 254-260.

208. Pengpid S, Peltzer K (2012) Hygiene behaviour and health attitudes in African countries. Curr Opin Psychiatry 25: 149-154.

209. Jorge F, Carretero MA, Perera A, Harris DJ, Roca V (2012) A new species of Spauligodon (Nematoda: Oxyurida: Pharyngodonidae) in geckos from Sao Nicolau Island (Cape Verde) and its phylogenetic assessment. J Parasitol 98: 160-166.

210. Pezzani BC, Minvielle MC, De Luca MM, Radman N, Iacoy P, et al. (1996) [Intestinal parasite infections in a periurban community from the Province of Buenos Aires, Argentina]. Int J Parasitol 26: 105-108.

211. Pezzani BC, Minvielle MC, Ciarmela ML, Apezteguia MC, Basualdo JA (2009) [Community participation in the control of intestinal parasitoses at a rural site in Argentina]. Rev Panam Salud Publica 26: 471-477.

212. Pinheiro Ide O, de Castro MF, Mitterofhe A, Pires FA, Abramo C, et al. (2011) Prevalence and risk factors for giardiasis and soil-transmitted helminthiasis in three municipalities of Southeastern Minas Gerais State, Brazil: risk factors for giardiasis and soil-transmitted helminthiasis. Parasitol Res 108: 1123-1130.

213. Pruss A, Kay D, Fewtrell L, Bartram J (2002) Estimating the burden of disease from water, sanitation, and hygiene at a global level. J Cell Sci 115: 2293-2302.

214. Pullan RL, Bethony JM, Geiger SM, Cundill B, Correa-Oliveira R, et al. (2008) Human helminth co-infection: analysis of spatial patterns and risk factors in a Brazilian community. PLoS Negl Trop Dis 2: e352.

215. Pullan RL, Kabatereine NB, Quinnell RJ, Brooker S (2011) Spatial and genetic epidemiology of hookworm in a rural community in Uganda. Arch Environ Contam Toxicol 60: 479-485. doi: 410.1007/s00244-00010-09547-00249. Epub 02010 Jun 00218.

216. Quihui L, Valencia ME, Crompton DW, Phillips S, Hagan P, et al. (2006) Role of the employment status and education of mothers in the prevalence of intestinal parasitic infections in Mexican rural schoolchildren. Vet Immunol Immunopathol 114: 135-148. Epub 2006 Sep 2007.

217. Rai SK, Hirai K, Ohno Y, Matsumura T (1997) Village health and sanitary profile from eastern hilly region, Nepal. Rev Sci Tech 16: 382-390.

218. Rai DR, Rai SK, Sharma BK, Ghimire P, Bhatta DR (2004) Factors associated with intestinal parasitic infection among school children in a rural area of Kathmandu Valley, Nepal. Nepal Med Coll J 6: 78-82.

219. Rai SK, Gurung R, Saiju R, Bajracharya L, Rai N, et al. (2008) Intestinal parasitosis among subjects undergoing cataract surgery at the eye camps in rural hilly areas of Nepal. Acta Vet Hung 56: 335-340. doi: 310.1556/AVet.1556.2008.1553.1557.

220. Raja'a YA, Sulaiman SM, Mubarak JS, El-Bakri MM, Al-Adimi WH, et al. (2001) Some aspects in the control of schistosomosis and soil-transmitted helminthosis in Yemeni children. Saudi Med J 22: 428-432.

221. Raja'a YA, Sulaiman SM, Mubarak JS, El-Bakri MM, Al-Adimi WH, et al. (2001) Some aspects in the control of schistosomosis and soil-transmitted helminthosis in Yemeni children. Trends Parasitol 17: 256-261.

222. Rajeswari B, Sinniah B, Hussein H (1994) Socio-economic factors associated with intestinal parasites among children living in Gombak, Malaysia. Rev Epidemiol Sante Publique 42: 322-333.

223. Ramesh GN, Malla N, Raju GS, Sehgal R, Ganguly NK, et al. (1991) Epidemiological study of parasitic infestations in lower socio-economic group in Chandigarh (north India). J Wildl Dis 27: 102-104.

224. Ratard RC, Kouemeni LE, Ekani Bessala MM, Ndamkou CN, Sama MT, et al. (1991) Ascariasis and trichuriasis in Cameroon. Trans R Soc Trop Med Hyg 85: 84-88.

225. Rebollo M, Tchuente T, Perea C, Polentinos E, Bueno A, et al. (2011) Prevalence of soil transmitted helminthiasis on school age children in south Cameroon. Trop Med Int Health 16: 223.

226. Reinhard KJ (1994) Sanitation and parasitism at Harpers-Ferry, West Virginia. Historical Archaeology 28: 62-67.

227. Righetti AA, Koua AYG, Adiossan LG, Glinz D, Hurrell RF, et al. (2012) Etiology of anemia among infants, school-aged children, and young non-pregnant women in different settings of South-Central Cote d'Ivoire. Am J Trop Med Hyg 87: 425-434.

228. Rinne S, Rodas EJ, Galer-Unti R, Glickman N, Glickman LT (2005) Prevalence and risk factors for protozoan and nematode infections among children in an Ecuadorian highland community. Trans R Soc Trop Med Hyg 99: 585-592.

229. Roka M, Goni P, Rubio E, Clavel A (2012) Prevalence of intestinal parasites in HIV-positive patients on the island of Bioko, Equatorial Guinea: its relation to sanitary conditions and socioeconomic factors. Sci Total Environ 432:404-11.: 10.1016/j.scitotenv.2012.1006.1023. Epub 2012 Jul 1014.

230. Rollemberg CV, Santos CM, Silva MM, Souza AM, Silva AM, et al. (2011) [Epidemiological characteristics and geographical distribution of schistosomiasis and geohelminths, in the State of Sergipe, according to data from the Schistosomiasis Control Program in Sergipe]. Rev Soc Bras Med Trop 44: 91-96.

231. Rostami M, Tohidi F, Sharbatkhori M, Taherkhani H, Eteraf A (2011) Prevalence of intestinal parasitic infections in primary school students in Gorgan, Iran. Trop Med Int Health 16: 307-308.

232. Ruiz Lopes FM, Goncalves DD, Dos Reis CR, Bregano RM, Filho FA, et al. (2006) Occurrence of enteroparasitosis in schoolchildren of the municipal district of Jataizinho, State of Parana, Brazil. Acta Scientiarum - Health Sciences 28: 107-111.

233. Ryngajllo A, Bak-Romaniszyn L, Ludzik M, Mielczarek J, Malecka-Panas E (2008) Evaluation of risk factors of Ascaris species infection in children in own material. Family Medicine and Primary Care Review 10: 1325-1329.

234. Saathoff E, Olsen A, Sharp B, Kvalsvig JD, Appleton CC, et al. (2005) Ecologic covariates of hookworm infection and reinfection in rural Kwazulu-Natal/South Africa: A geographic information system-based study. Am J Trop Med Hyg 72: 384-391.

235. Sadaga GA, Kassem HH (2007) Prevalence of intestinal parasites among primary schoolchildren in Derna District, Libya. J Egypt Soc Parasitol 37: 205-214.

236. Sadek Y, el-Fakahany AF, Lashin AH, el-Salam FA (1997) Intestinal parasites among food-handlers in Qualyobia Governorate, with reference to the pathogenic parasite blastocystis hominis. J Egypt Soc Parasitol 27: 571-579.

237. Sadun EH, Vajrasthira S, Maiphoom C (1954) The effect of treatment and sanitation on hookworm infection in Cholburi Province (Central Thailand). J Comp Pathol 64: 195-205.

238. Salazar-Labori E, Navarro C, Montero M, Nino-Incani R, Cortez J, et al. (2011) Epidemiology of intestinal parasitosis in eleven states of venezuela: Partial results of an ongoing national survey. Clin Microbiol Infect 17: S213.

239. Sanches WR, Wagner EG (1954) Experience with excreta-disposal programmes in rural areas of Brazil. Bull World Health Organ 10: 229-249.

240. Reardon C, Sanchez A, Hogaboam CM, McKay DM (2001) Tapeworm infection reduces epithelial ion transport abnormalities in murine dextran sulfate sodium-induced colitis. Infect Immun 69: 4417-4423.

241. Schlosser O, Grall D, Laurenceau MN (1999) Intestinal parasite carriage in workers exposed to sewage. Arch Environ Contam Toxicol 37: 190-195.

242. Scholte RG, Freitas CC, Dutra LV, Guimaraes RJ, Drummond SC, et al. (2012) Intestinal parasitosis and shigellosis among diarrheal patients in Gondar teaching hospital, northwest Ethiopia. Acta Trop 121: 112-117. doi: 110.1016/j.actatropica.2011.1010.1011. Epub 2011 Oct 1021.

243. Schonning C, Westrell T, Stenstrom TA, Arnbjerg-Nielsen K, Hasling AB, et al. (2007) Microbial risk assessment of local handling and use of human faeces. J Water Health 5: 117-128.

244. Seidu R, Heistad A, Amoah P, Drechsel P, Jenssen PD, et al. (2008) Quantification of the health risk associated with wastewater reuse in Accra, Ghana: a contribution toward local guidelines. J Water Health 6: 461-471.

245. Shakya B, Shrestha S, Madhikarmi NL, Adhikari R (2012) Intestinal parasitic infection among school children. J Nepal Health Res Counc 10: 20-23.

246. Shang Y, Tang LH, Zhou SS, Chen YD, Yang YC, et al. (2010) Stunting and soil-transmitted-helminth infections among school-age pupils in rural areas of southern China. Parasit Vectors 3: 97.

247. Sharma BK, Rai SK, Rai DR, Choudhury DR (2004) Prevalence of intestinal parasitic infestation in schoolchildren in the northeastern part of Kathmandu Valley, Nepal. Southeast Asian J Trop Med Public Health 35: 506-511.

248. Shobha M, Bithika D, Bhavesh S (2013) The prevalence of intestinal parasitic infections in the urban slums of a city in Western India. J Infect Public Health 6: 142-149. doi: 110.1016/j.jiph.2012.1011.1004. Epub 2013 Jan 1021.

249. Shrestha A, Narayan KC, Sharma R (2012) Prevalence of intestinal parasitosis among school children in Baglung district of Nepal. Kathmandu Univ Med J 10: 3-6.

250. Siharath K, Soukphathag S, Tanyavong K, Vilaysane KD, Chanthavong M, et al. (2001) Control of intestinal parasitic infection--a pilot project in Lao PDR. Parasite Immunol 23: 411-417.

251. de Cassia Ribeiro Silva R, Barreto ML, Assis AM, de Santana ML, Parraga IM, et al. (2007) The relative influence of polyparasitism, environment, and host factors on schistosome infection. Am J Trop Med Hyg 77: 672-675.

252. Silva JC, Furtado LF, Ferro TC, Bezerra Kde C, Borges EP, et al. (2011) [Parasitism due to *Ascaris lumbricoides* and its epidemiological characteristics among children in the State of Maranhao]. Rev Soc Bras Med Trop 44: 100-102.

253. Singh C, Zargar SA, Masoodi I, Shoukat A, Ahmad B (2011) Predictors of intestinal parasitosis in school children of Kashmir: a prospective study. J Helminthol 85: 294-299. doi: 210.1017/S0022149X10000581. Epub 10002010 Sep 10000524.

254. Smith H, Dekaminsky R, Niwas S, Soto R, Jolly P (2001) Prevalence and intensity of infections of *Ascaris lumbricoides* and *Trichuris trichiura* and associated socio-demographic variables in four rural Honduran communities. Mem Inst Oswaldo Cruz 96: 303-314.

255. Aidar Sobrinho T, Coelho LM, de Oliveira SM, Martins Jde T, Rabello Junior JA, et al. (1995) [Frequency of intestinal helminth eggs in public restrooms in Sorocaba, SP]. Rev Soc Bras Med Trop 28: 33-37.

256. Sorensen E, Ismail M, Amarasinghe DK, Hettiarachchi I, Dassenaieke TS (1994) The effect of the availability of latrines on soil-transmitted nematode infections in the plantation sector in Sri Lanka. Am J Trop Med Hyg 51: 36-39.

257. Soroczan W (1976) Strongyloides stercoralis in Eastern and South-Eastern Poland. Wiad Parazytol 22: 517-520.

258. Sultana Y, Gilbert GL, Ahmed B-N, Lee R (2012) Strongyloidiasis in a high risk community of Dhaka, Bangladesh. Trans R Soc Trop Med Hyg 106: 756-762.

259. Suputtamongkol Y, Changngern N, Waywa D, Silpasakorn S, Anekthananon T, et al. (2012) Prevalence and risk factors for acquiring *Strongyloides stercoralis* infection among patients attending a tertiary hospital in Thailand. Clin Microbiol Infect 18: 600.

260. Tagajdid R, Lemkhente Z, Errami M, El Mellouki W, Lmimouni B (2011) [Prevalence of intestinal parasitic infections in Moroccan urban primary school students]. Mol Reprod Dev 78: 151-160. doi: 110.1002/mrd.21289. Epub 22011 Feb 21217.

261. Tamer GS, Erdogan S, Willke A (2008) [The frequency of the presence of intestinal parasites in students of Arslanbey Primary School]. Turkiye Parazitol Derg 32: 171-173.

262. Tang CM, Huang JJ (1993) [Field studies on different control schemes for hookworm infection]. Med Dosw Mikrobiol 45: 511-515.

263. Tanner S, Chuquimia-Choque ME, Huanca T, McDade TW, Leonard WR, et al. (2011) The effects of local medicinal knowledge and hygiene on helminth infections in an Amazonian society. Soc Sci Med 72: 701-709.

264. Taranto NJ, Cajal SP, De Marzi MC, Fernandez MM, Frank FM, et al. (2003) Clinical status and parasitic infection in a Wichi Aboriginal community in Salta, Argentina. Trans R Soc Trop Med Hyg 97: 554-558.

265. Tashima NT, Simoes MJ (2004) Enteroparasitic occurrence in fecal samples analyzed at the University of Western Sao Paulo-UNOESTE Clinical Laboratory, Presidente Prudente, Sao Paulo State, Brazil. Parasitol Res 94: 449-451. Epub 2004 Oct 2028.

266. Tatala SR, Kihamia CM, Kyungu LH, Svanberg U (2009) Risk factors for anaemia in schoolchildren in Tanga Region, Tanzania. BMC Genomics 10:202.: 10.1186/1471-2164-1110-1202.

267. Tavares-Dias M, Grandini AA (1999) Prevalence and epidemiological aspects of enteroparasites in the population of Sao Jose da Bela Vista, Sao Paulo. Rev Soc Bras Med Trop 32: 63-65.

268. Tellez A, Morales W, Rivera T, Meyer E, Leiva B, et al. (1997) Prevalence of intestinal parasites in the human population of Leon, Nicaragua. Acta Trop 66: 119-125.

269. Temcharoen P, Chularerk U, Viboolyavatana J (1987) Intestinal parasitoses among the workers and their families at three electricity generating dams in Thailand. Parasitol Today 3: 94-98.

270. Thanchomnang T, Intapan P, Sri-Aroon P, Lulitanond V, Janwan P, et al. (2011) Geographical analysis of the role of water supply and sanitation in the risk of helminth infections of children in West Africa. Mem Inst Oswaldo Cruz 106: 831-836.

271. Thofern E (1989) The success of the hygiene during the last forty years. Zentralblatt fur Bakteriologie Mikrobiologie und Hygiene - Abt 1 Orig B Umwelthygiene, Krankenhaushygiene, Arbeitshygiene, Praeventive Medizin 187: 271-294.

272. Tikasingh ES, Chadee DD, Rawlins SC (2011) The control of hookworm disease in Commonwealth Caribbean countries. Parasit Vectors 4:149.: 10.1186/1756-3305-1184-1149.

273. Toma A, Miyagi I, Kamimura K, Tokuyama Y, Hasegawa H, et al. (1999) Questionnaire survey and prevalence of intestinal helminthic infections in Barru, Sulawesi, Indonesia. Southeast Asian J Trop Med Public Health 30: 68-77.

274. Tomono N, Anantaphruti MT, Jongsuksuntigul P, Thongthien P, Leerapan P, et al. (2003) Risk factors of helminthiases among schoolchildren in southern Thailand. Southeast Asian J Trop Med Public Health 34: 269-273.

275. Torres P, Otth L, Montefusco A, Wilson G, Ramirez C, et al. (1997) [Infection by intestinal protozoa and helminths in schoolchildren from riverside sectors, with different fecal contamination levels, of Valdivia River, Chile]. Bol Chil Parasitol 52: 12-17.

276. Tronnberg L, Hawksworth D, Hansen A, Archer C, Stenstrom TA (2010) Household-based prevalence of helminths and parasitic protozoa in rural KwaZulu-Natal, South Africa, assessed from faecal vault sampling. Trans R Soc Trop Med Hyg 104: 646-652.

277. Tshikuka JG, Scott ME, Gray-Donald K (1995) *Ascaris lumbricoides* infection and environmental risk factors in an urban African setting. Ann Trop Med Parasitol 89: 505-514.

278. Udonsi JK, Amabibi MI (1992) The human environment, occupation, and possible water-borne transmission of the human hookworm, Necator americanus, in endemic coastal communities of the Niger Delta, Nigeria. Toxicology 73: 305-321.

279. Ulukanligil M, Seyrek A (2003) Demographic and parasitic infection status of schoolchildren and sanitary conditions of schools in Sanliurfa, Turkey. Rev Panam Salud Publica 14: 31-41.

280. Ulukanligil M (2008) School-based deworming programme in Sanliurfa, Turkey: Changing from externally funding phase to self-sufficient phase. J Trop Pediatr 54: 157-163.

281. van Eijk AM, Lindblade KA, Odhiambo F, Peterson E, Rosen DH, et al. (2009) Geohelminth Infections among pregnant women in rural western Kenya; a cross-sectional study. Parasitol Res 104: 1351-1359. doi: 1310.1007/s00436-00009-01334-z. Epub 02009 Jan 00427.

282. van Niekerk CH, Weinberg EG, Shore SC, de Heese H (1979) Intestinal parasitic infestation in urban and rural Xhosa children. A comparative study. Med J Aust 1: 401-403.

283. Vanek E (1967) [Spread of intestinal helminths in East African children. Living habits as favoring or inhibiting factors]. Br Med J 1: 537-538.

284. Vasconcelos IAB, Oliveira JW, Cabral FRF, Coutinho HDM, Menezes IRA (2011) Prevalence of intestinal parasite infections among 4- to 12-year-old children in Crato, Ceara State. Acta Scientiarum - Health Sciences 33: 35-41.

285. Verle P, Kongs A, De NV, Thieu NQ, Depraetere K, et al. (2003) Prevalence of intestinal parasitic infections in northern Vietnam. Acta Trop 88: 117-130.

286. Wagbatsoma VA, Aimiuwu U (2008) Sanitary provision and helminthiasis among school children in Benin City, Nigeria. PLoS One 3: e2550. doi: 2510.1371/journal.pone.0002550.

287. Wang XB, Wang GF, Zhang LX, Luo RF, Tian HC, et al. (2012) [Investigation on prevalence of soil-transmitted nematode infections and influencing factors for children in southwest areas of China]. Zhongguo Xue Xi Chong Bing Fang Zhi Za Zhi 2012 Jun;24: 268-273.

288. Wani S, Ahmad F (2009) Intestinal helminths and associated risk factors in children of district Pulwama, Kashmir, India. Indian Journal of Medical Microbiology 27: 81-82.

289. Wani SA, Ahmad F, Zargar SA, Ahmad Z, Ahmad P, et al. (2007) Prevalence of intestinal parasites and associated risk factors among schoolchildren in Srinagar City, Kashmir, India. Trop Anim Health Prod 39: 97-102.

290. Wani SA, Ahmad F, Zargar SA, Dar PA, Dar ZA, et al. (2008) Intestinal helminths in a population of children from the Kashmir valley, India. J Helminthol 82: 313-317.

291. Wani SA, Ahmad F, Zargar SA, Amin A, Dar ZA, et al. (2010) Intestinal helminthiasis in children of gurez valley of jammu and kashmir state, India. J Glob Infect Dis 2: 91-94.

292. Warunee N, Choomanee L, Sataporn P, Rapeeporn Y, Nuttapong W, et al. (2007) Intestinal parasitic infections among school children in Thailand. Trop Biomed 24: 83-88.

293. Wen-Juan L, Shao-Rong C, Yan-Hong L, Wen F, Chun-Rong K, et al. (2011) [Evaluation on effectiveness of comprehensive control model for soil-transmitted nematodiasis]. Zhongguo Xue Xi Chong Bing Fang Zhi Za Zhi 23: 524-528.

294. White RMM, Pereira LH, Embil JA, Manuel FR (1986) *Ascaris lumbricoides* in Nova Scotia. Can J Public Health 77: 201-204.

295. Worrell CM, Davis SM, Wiegand RE, Lopez G, Cosmas L, et al. (2012) Water- and sanitation-related risk factors for soil-transmitted helminth infection in urban school- and preschool-aged children in Kibera, Nairobi. Am J Trop Med Hyg 87: 380-381.

296. Boes J, Willingham AL, 3rd, Fuhui S, Xuguang H, Eriksen L, et al. (2000) Prevalence and distribution of pig helminths in the Dongting Lake Region (Hunan Province) of the People's Republic of China. J Helminthol 74: 45-52.

297. Yajima A, Jouquet P, Do TD, Dang TC, Tran CD, et al. (2009) High latrine coverage is not reducing the prevalence of soil-transmitted helminthiasis in Hoa Binh province, Vietnam. Trans R Soc Trop Med Hyg 103: 237-241.

298. Yami A, Mamo Y, Kebede S (2011) Prevalence and predictors of intestinal helminthiasis among school children in jimma zone; a cross-sectional study. Ethiop J Health Sci 21: 167-174.

299. Youmbi JG, Feumba R, Njitat VT, de Marsily G, Ekodeck GE (2013) [Water pollution and health risks at Yaounde, Cameroon]. C R Biol 336: 310-316. doi: 310.1016/j.crvi.2013.1004.1013. Epub 2013 Jul 1015.

300. Zeynudin A, Hemalatha K, Kannan S (2013) Prevalence of opportunistic intestinal parasitic infection among HIV infected patients who are taking antiretroviral treatment at Jimma Health Center, Jimma, Ethiopia. Eur Rev Med Pharmacol Sci 17: 513-516.

301. Zheng Q, Chen Y, Zhang HB, Chen JX, Zhou XN (2009) The control of hookworm infection in China. Parasit Vectors 2.

302. Ziegelbauer K, Speich B, Mausezahl D, Bos R, Keiser J, et al. (2012) Effect of sanitation on soil-transmitted helminth infection: systematic review and meta-analysis. PLoS Med 9: e1001162.

303. Zulu S, Taylor M (2011) Has improved water and sanitation changed the prevalence of schistosomiasis and soil transmitted helminths (STH) amongst primary school aged children in Ugu district of KwaZulu-Natal. Am J Trop Med Hyg 85: 79-80.

304. Zulu SG, Taylor M, Kjetland EF, Kvalsvig J, Gundersen SG, et al. (2012) Has improved water and sanitation changed the prevalence of schistosomiasis and soil transmitted helminths amongst female primary school aged children in Ugu District of KwaZulu-Natal, southern Africa? Clin Microbiol Infect 18: 599.

305. Zulu SG, Kjetland EF, Taylor M (2013) Improved water access and clean sanitation alone may not be enough to significantly reduce the problem of schistosomiasis and soil transmitted helminths in Ugu district, South Africa. Trop Med Int Health 18: 123-123.
