# Supplementary material for: Water, Sanitation, Hygiene, and Soil-Transmitted Helminth Infection: A Systematic Review and Meta-Analysis
Source: PLoS Med. 2014 Mar 25;11(3):e1001620. doi: 10.1371/journal.pmed.1001620 (PMC3965411; doi:10.1371/journal.pmed.1001620)
Supplement: Table S2 — Study bias assesment. (DOC) [file pmed.1001620.s016.doc]

Table S2. Study bias assessment

| ***Study Info*** | ***GRADE Assessment*** | | | | | |
| --- | --- | --- | --- | --- | --- | --- |
| **Study Author, Year** | **Diagnostic** | **Exposure Assessment** | **Confounding Assessment** | **Response Rate** | **Selective Reporting** | **Over All** |
| Ahmed [1] | Low | High | Low | Low | Low | Low |
| Aimpun [2] | Low | High | Low | Low | High | Low |
| Alemu [3,4] | Low | Low | High | Unclear | Low | Low |
| Al-Mekhlafi [5] | Low | High | Low | Low | High | Unclear |
| Al-Mekhlafi [6] | Low | High | Unclear | Low | Low | Low |
| Alvarado [7] | Low | High | Low | Low | Low | Low |
| Amahmid [8] | Low | Low | High | Unclear | Low | Low |
| Asaolu [9] | Low | High | Low | Low | High | Unclear |
| Awasthi [10] | Low | High | High | Unclear | Low | High |
| Balen [11] | Low | High | Low | Low | High | Unclear |
| Barreto [12] | Low | Low | Low | High | Low | Low |
| Basualdo [13] | Low | High | Low | Low | High | High |
| Belo [14] | Low | High | High | Low | Low | Unclear |
| Belyhun [15] | Low | High | Low | Low | Low | Low |
| Bieri [16] | Low | Low | Low | Low | Low | Low |
| Carneiro [17] | Low | High | Low | Low | Low | Low |
| Chongsuvivatwong [18] | Low | Low | Low | Unclear | Low | Low |
| Corrales [19] | Low | Low | Low | Low | Low | Low |
| Cundill [20] | Low | High | Low | Low | Low | Low |
| Dumba[21] | Low | Low | Low | High | Low | Low |
| Ellis [22] | Low | High | High | Low | Low | High |
| Ensink [23] | Low | High | Low | Low | Low | Low |
| Farook [24] | Low | High | High | Unclear | High | High |
| Ferreira [25] | Low | High | Low | Low | Low | Low |
| Fonseca [26] | Low | High | Low | Low | Low | Low |
| Freeman [27] | Low | Low | Low | Low | Low | Low |
| Geissler [28] | Low | Low | High | Low | Low | Low |
| Glickman [29] | Low | High | Low | Low | High | Low |
| Gunawardena [30] | Low | High | Low | Low | High | Unclear |
| Gunawardena [31] | Low | High | Low | Low | High | Unclear |
| Gunawardena [32] | Low | High | Unclear | Low | High | Unclear |
| Guo-Fei [33] | Low | Unclear | Low | Unclear | Low | Unclear |
| Gyorkos [34] | Low | High | Low | Low | High | Unclear |
| Gyorkos [35] | Low | Low | Low | Low | Low | Low |
| Habbari [36] | Low | High | High | Unclear | Low | High |
| Hall [37] | Low | High | High | Low | Low | High |
| Halpenny [38] | Low | High | Low | Low | High | Unclear |
| Henry [39] | High | Low | High | Unclear | Low | High |
| Hidayah [40] | Low | High | Unclear | Low | Low | Low |
| Hohmann [41] | Low | High | Low | Low | High | Unclear |
| Huat [42] | Low | High | Low | Low | High | Low |
| Hughes [43] | Low | Low | Low | Unclear | Low | Low |
| Humphries [44] | Low | High | Low | Low | High | Unclear |
| Ivan [45] | Low | High | Low | Low | Low | Low |
| Jiraanankul [46] | Low | High | Low | Low | Low | Low |
| Khieu [47] | Low | High | High | Low | Low | High |
| Knopp [48] | Low | High | Low | Unclear | Low | Low |
| Kounnavong [49] | Low | High | Low | Low | Low | Low |
| Koura [50] | Low | Unclear | Unclear | Low | Unclear | Unclear |
| Lee [51] | Low | High | Low | Low | Low | Low |
| Luoba [52] | Low | High | High | Low | Low | High |
| Mahmud [53] | Low | Low | Low | Low | Unclear | Low |
| Matthys [54] | Low | High | Low | Low | Low | Low |
| Mihrshahi [55] | Low | High | Low | Unclear | High | High |
| Moraes [56] | Low | Low | Low | Low | Low | Low |
| Moraes [57] | Low | Unclear | Low | Low | Low | Low |
| Morales-Espinoza [58] | Low | High | Low | Low | Low | Low |
| Narain [59] | Low | High | Low | Low | Low | Low |
| Nasr [60] | Low | Low | Low | Low | Low | Low |
| Nguyen [61] | Low | High | Low | Low | High | Unclear |
| Nishiura [62] | Low | High | Low | Low | Low | Low |
| Norhayati [63] | Low | High | High | Unclear | High | High |
| Nwaneri [64] | Low | High | Low | Low | High | Unclear |
| Olsen [65] | Low | Unclear | Low | Low | High | Unclear |
| Ortiz Valencia [66] | High | High | High | Unclear | Low | High |
| Parajuli [67] | Low | High | Low | Low | Low | Low |
| Pham-Duc [68] | Low | High | Low | Low | Low | Low |
| Phiri [69] | Low | High | Low | Low | High | Unclear |
| Quintero [70] | Low | High | Low | Low | Low | Low |
| Riess [71] | Low | High | Low | Low | Low | Low |
| Rísquez [72] | Low | High | High | Low | Low | High |
| Roy [73] | Low | Unclear | Low | Low | Unclear | Unclear |
| Saathoff [74] | Low | High | Low | Unclear | Low | Low |
| Schmidlin [75] | Low | High | Low | High | High | High |
| Scolari [76] | Low | Low | High | Low | Low | Low |
| Sherkhonov [77] | Low | High | Low | Low | Low | Low |
| Soares Magalhaes [78] | Low | High | Unclear | Unclear | Low | Unclear |
| Steenhard [79] | Low | High | Low | Low | Unclear | Low |
| Steinmann [80] | Low | High | Low | Low | Low | Low |
| Stothard [81] | Low | High | Low | Low | Low | Low |
| Teixeira[82] | Low | High | Low | Low | Low | Low |
| Trang [83] | Low | High | Low | Low | Low | Low |
| Trang [84] | Low | High | Low | Low | Low | Low |
| Traub [85] | Low | High | Low | Low | Low | Low |
| Ugbomoiko [86] | Low | High | Low | Low | High | High |
| Walker [87] | Low | High | Low | Low | Low | Low |
| Wang [88] | Low | High | Low | Low | Low | Low |
| Wordemann [89] | Low | High | Low | Low | Low | Low |
| Worrell [90] | Low | Low / High | Low | Low | High | Low |
| Xu [91] | Low | Low | Low | Low | Low | Low |
| Yajima [92] | Low | High | High | Unclear | Low | High |
| Yori [93] | Low | High | Low | High | Low | High |
| Young [94] | Low | High | Low | Unclear | Low | Low |

**References**

1. Ahmed A, Al-Mekhlafi HM, Choy SH, Ithoi I, Al-Adhroey AH, et al. (2011) The burden of moderate-to-heavy soil-transmitted helminth infections among rural malaysian aborigines: an urgent need for an integrated control programme. Parasit Vectors 4: 242.

2. Aimpun P, Hshieh P (2004) Survey for intestinal parasites in Belize, Central America. Southeast Asian J Trop Med Public Health 35: 506-511.

3. Alemu A, Atnafu A, Addis Z, Shiferaw Y, Teklu T, et al. (2011) Soil transmitted helminths and schistosoma mansoni infections among school children in zarima town, northwest Ethiopia. BMC Infect Dis 11.

4. Alemu A, Atnafu A, Addis Z, Shiferaw Y, Teklu T, et al. (2012) Soil transmitted helminths and schistosoma mansoni infections among school children in Zarima town, northwest Ethiopia. Parasitol Int 61: 101-106. doi: 110.1016/j.parint.2011.1006.1018. Epub 2011 Jun 1029.

5. Al-Mekhlafi MS, Atiya AS, Lim YA, Mahdy AK, Ariffin WA, et al. (2007) An unceasing problem: soil-transmitted helminthiases in rural Malaysian communities. Southeast Asian J Trop Med Public Health 38: 998-1007.

6. Al-Mekhlafi HM, Surin J, Atiya AS, Ariffin WA, Mohammed Mahdy AK, et al. (2008) Pattern and predictors of soil-transmitted helminth reinfection among aboriginal schoolchildren in rural Peninsular Malaysia. Acta Trop 107: 200-204.

7. Alvarado BE, Vasquez LR (2006) [Social determinants, feeding practices and nutritional consequences of intestinal parasitism in young children]. Biomedica 26: 82-94.

8. Amahmid O, Bouhoum K (2005) Assessment of the health hazards associated with wastewater reuse: transmission of geohelminthic infections (Marrakech, Morocco). Ann Agric Environ Med 12: 35-38.

9. Asaolu SO, Ofoezie IE, Odumuyiwa PA, Sowemimo OA, Ogunniyi TA (2002) Effect of water supply and sanitation on the prevalence and intensity of *Ascaris lumbricoides* among pre-school-age children in Ajebandele and Ifewara, Osun State, Nigeria. Trans R Soc Trop Med Hyg 96: 600-604.

10. Awasthi S, Verma T, Kotecha P, Venkatesh V, Joshi V, et al. (2008) Prevalence and risk factors associated with worm infestation in pre-school children (6-23 months) in selected blocks of Uttar Pradesh and Jharkhand, India. Indian J Med Sci 62: 484-491.

11. Balen J, Raso G, Li YS, Zhao ZY, Yuan LP, et al. (2011) Risk factors for helminth infections in a rural and a peri-urban setting of the Dongting Lake area, People's Republic of China. Int J Parasitol 41: 1165-1173.

12. Barreto ML, Genser B, Strina A, Teixeira MG, Assis AMO, et al. (2010) Impact of a citywide sanitation program in Northeast Brazil on intestinal parasites infection in young children. Environ Health Perspect 118: 1637-1642.

13. Basualdo JA, Cordoba MA, de Luca MM, Ciarmela ML, Pezzani BC, et al. (2007) Intestinal parasitoses and environmental factors in a rural population of Argentina, 2002-2003. Bull Soc Pathol Exot 100: 174-175.

14. Belo S, Rompao H, Goncalves L, Gracio MA (2005) Prevalence, behavioural and social factors associated with *Schistosoma intercalatum* and geohelminth infections in Sao Tome and Principe. Parassitologia 47: 227-231.

15. Belyhun Y, Medhin G, Amberbir A, Erko B, Hanlon C, et al. (2010) Prevalence and risk factors for soil-transmitted helminth infection in mothers and their infants in Butajira, Ethiopia: a population based study. BMC Public Health 10: 21.

16. Bieri FA, Gray DJ, Williams GM, Raso G, Li YS, et al. (2013) Health-education package to prevent worm infections in Chinese schoolchildren. The New England journal of medicine 368: 1603-1612.

17. Carneiro FF, Cifuentes E, Tellez-Rojo MM, Romieu I (2002) The risk of *Ascaris lumbricoides* infection in children as an environmental health indicator to guide preventive activities in Caparao and Alto Caparao, Brazil. Bull World Health Organ 80: 40-46.

18. Chongsuvivatwong V, Pas-Ong S, McNeil D, Geater A, Duerawee M (1996) Predictors for the risk of hookworm infection: experience from endemic villages in southern Thailand. Trans R Soc Trop Med Hyg 90: 630-633.

19. Corrales LF, Izurieta R, Moe CL (2006) Association between intestinal parasitic infections and type of sanitation system in rural El Salvador. Trop Med Int Health 11: 1821-1831.

20. Cundill B, Alexander N, Bethony JM, Diemert D, Pullan RL, et al. (2011) Rates and intensity of re-infection with human helminths after treatment and the influence of individual, household, and environmental factors in a Brazilian community. Parasitology 138: 1406-1416.

21. Dumba R, Kaddu JB, Wabwire-Mangen F (2013) Design and implementation of participatory hygiene and sanitation transformation (PHAST) as a strategy to control soil-transmitted helminth infections in Luweero, Uganda. Afr Health Sci 13: 512-517.

22. Ellis MK, Raso G, Li YS, Rong Z, Chen HG, et al. (2007) Familial aggregation of human susceptibility to co- and multiple helminth infections in a population from the Poyang Lake region, China. Int J Parasitol 37: 1153-1161.

23. Ensink JH, van der Hoek W, Mukhtar M, Tahir Z, Amerasinghe FP (2005) High risk of hookworm infection among wastewater farmers in Pakistan. Proc Natl Acad Sci U S A 102: 12449-12454. Epub 12005 Aug 12412.

24. Farook MU, Sudharmini S, Remadevi S, Vijayakumar K (2002) Intestinal helminthic infestations among tribal populations of Kottoor and Achankovil areas in Kerala (India). The Journal of communicable diseases 34: 171-178.

25. Ferreira MU, Ferreira CD, Monteiro CA (2000) Secular trends in child intestinal parasitic diseases in S. Paulo city, Brazil (1984-1996). Rev Saude Publica 34: 73-82.

26. Fonseca EO, Teixeira MG, Barreto ML, Carmo EH, Costa Mda C (2010) [Prevalence and factors associated with geohelminth infections in children living in municipalities with low HDI in North and Northeast Brazil]. Cad Saude Publica 26: 143-152.

27. Freeman MC, Clasen T, Brooker S, Akoko D, Rheingans R (2013) The impact of a school-based hygiene, water quality and sanitation intervention on soil-transmitted helminth re-infection: A cluster-randomized trial. Am J Trop Med Hyg 89: 875-883.

28. Geissler PW, Mwaniki D, Thiong'o F, Friis H (1998) Geophagy as a risk factor for geohelminth infections: A longitudinal study of Kenyan primary schoolchildren. Trans R Soc Trop Med Hyg 92: 7-11.

29. Glickman LT, Camara AO, Glickman NW, McCabe GP (1999) Nematode intestinal parasites of children in rural Guinea, Africa: Prevalence and relationship to geophagia. Int J Epidemiol 28: 169-174.

30. Gunawardena GS, Karunaweera ND, Ismail MM (2004) Socio-economic and behavioural factors affecting the prevalence of *Ascaris* infection in a low-country tea plantation in Sri Lanka. Ann Trop Med Parasitol 98: 615-621.

31. Gunawardena GS, Karunaweera ND, Ismail MM (2005) Effects of climatic, socio-economic and behavioural factors on the transmission of hookworm (*Necator americanus*) on two low-country plantations in Sri Lanka. Ann Trop Med Parasitol 99: 601-609.

32. Gunawardena K, Kumarendran B, Ebenezer R, Gunasingha MS, Pathmeswaran A, et al. (2011) Soil-transmitted helminth infections among plantation sector schoolchildren in Sri Lanka: prevalence after ten years of preventive chemotherapy. PLoS Negl Trop Dis 5: e1341.

33. Guo-Fei W, Ying-Dan C, Chang-Hai Z, Ting-Jun Z (2011) [Analysis of influencing factors of *Trichuris trichiura* infection in demonstration plots of comprehensive control of parasitic diseases]. Chinese journal of schistosomiasis control 23: 495-500.

34. Gyorkos TW, Maheu-Giroux M, Blouin B, Casapia M (2011) Exploring determinants of hookworm infection in peruvian schoolchildren using a gender analysis. Am J Epidemiol 173: S224.

35. Gyorkos TW, Maheu-Giroux M, Blouin B, Casapia M (2013) Impact of health education on soil-transmitted helminth infections in schoolchildren of the Peruvian Amazon: a cluster-randomized controlled trial. PLoS Negl Trop Dis 7: e2397. doi: 2310.1371/journal.pntd.0002397.

36. Habbari K, Tifnouti A, Bitton G, Mandil A (2001) Geohelminthic infections associated with raw wastewater reuse for agricultural purposes in Beni-Mellal, Morocco. J Parasitol 87: 169-172.

37. Hall A, Conway DJ, Anwar KS, Rahman ML (1994) *Strongyloides stercoralis* in an urban slum community in Bangladesh: factors independently associated with infection. Trans R Soc Trop Med Hyg 88: 527-530.

38. Halpenny CM, Paller C, Koski KG, Valdes VE, Scott ME (2013) Regional, Household and Individual Factors that Influence Soil Transmitted Helminth Reinfection Dynamics in Preschool Children from Rural Indigenous Panama. PLoS Negl Trop Dis 7: e2070.

39. Henry FJ (1988) Reinfection with Ascaris lumbricoides after chemotherapy: a comparative study in three villages with varying sanitation. Ann Parasitol Hum Comp 63: 448-454.

40. Hidayah NI, Teoh ST, Hillman E (1997) Socio-environmental predictors of soil-transmitted helminthiasis in a rural community in Malaysia. The Southeast Asian journal of tropical medicine and public health 28: 811-815.

41. Hohmann H, Panzer S, Phimpachan C, Southivong C, Schelp FP (2001) Relationship of intestinal parasites to the environment and to behavioral factors in children in the Bolikhamxay Province of Lao PDR. Southeast Asian J Trop Med Public Health 32: 4-13.

42. Huat LB, Mitra AK, Noor Jamil NI, Dam PC, Jan Mohamed HJ, et al. (2012) Prevalence and risk factors of intestinal helminth infection among rural Malay children. Journal of Global Infectious Diseases 4: 10-14.

43. Hughes RG, Sharp DS, Hughes MC, Akau'ola S, Heinsbroek P, et al. (2004) Environmental influences on helminthiasis and nutritional status among Pacific schoolchildren. Int J Environ Health Res 14: 163-177.

44. Humphries D, Mosites E, Otchere J, Twum WA, Woo L, et al. (2011) Epidemiology of hookworm infection in Kintampo North Municipality, Ghana: patterns of malaria coinfection, anemia, and albendazole treatment failure. Am J Trop Med Hyg 84: 792-800.

45. Ivan E, Crowther NJ, Mutimura E, Osuwat LO, Janssen S, et al. (2013) Helminthic infections rates and malaria in HIV-infected pregnant women on anti-retroviral therapy in Rwanda. PLoS Negl Trop Dis 7: e2380. doi: 2310.1371/journal.pntd.0002380.

46. Jiraanankul V, Aphijirawat W, Mungthin M, Khositnithikul R, Rangsin R, et al. (2011) Incidence and risk factors of hookworm infection in a rural community of central Thailand. Am J Trop Med Hyg 84: 594-598.

47. Khieu V, Schar F, Marti H, Sayasone S, Duong S, et al. (2013) Diagnosis, treatment and risk factors of *Strongyloides stercoralis* in schoolchildren in Cambodia. PLoS Negl Trop Dis 7: e2035.

48. Knopp S, Stothard JR, Rollinson D, Mohammed KA, Khamis IS, et al. (2011) From morbidity control to transmission control: time to change tactics against helminths on Unguja Island, Zanzibar. PLoS Genet 7: e1001384. doi: 1001310.1001371/journal.pgen.1001384. Epub 1002011 May 1001312.

49. Kounnavong S, Vonglokham M, Houamboun K, Odermatt P, Boupha B (2011) Soil-transmitted helminth infections and risk factors in preschool children in southern rural Lao People's Democratic Republic. Trans R Soc Trop Med Hyg 105: 160-166.

50. Koura GK, Briand V, Massougbodji A, Cot M, Garcia A (2011) Prevalence and risk factors for soil-transmitted helminth infection in beninese women during pregnancy. Am J Epidemiol 173: S225.

51. Lee VJ, Ong A, Lee NG, Lee WT, Fong KL, et al. (2007) Hookworm infections in Singaporean soldiers after jungle training in Brunei Darussalam. Vet Parasitol 150: 128-138. Epub 2007 Oct 2024.

52. Luoba AI, Geissler PW, Estambale B, Ouma JH, Alusala D, et al. (2005) Earth-eating and reinfection with intestinal helminths among pregnant and lactating women in western Kenya. Trop Med Int Health 10: 220-227.

53. Mahmud MA, Spigt M, Bezabih AM, Pavon IL, Dinant GJ, et al. (2013) Risk factors for intestinal parasitosis, anaemia, and malnutrition among school children in Ethiopia. Pathogens and Global Health 107: 58-65.

54. Matthys B, Tschannen AB, Tian-Bi NT, Comoe H, Diabate S, et al. (2007) Risk factors for *Schistosoma mansoni* and hookworm in urban farming communities in western Cote d'Ivoire. Trop Med Int Health 12: 709-723.

55. Mihrshahi S, Casey GJ, Montresor A, Phuc TQ, Thach DTC, et al. (2009) The effectiveness of 4 monthly albendazole treatment in the reduction of soil-transmitted helminth infections in women of reproductive age in Viet Nam. Int J Parasitol 39: 1037-1043.

56. Moraes LR, Cancio JA, Cairncross S (2004) Impact of drainage and sewerage on intestinal nematode infections in poor urban areas in Salvador, Brazil. Trans R Soc Trop Med Hyg 98: 197-204.

57. Moraes LRS (2007) Household solid waste bagging and collection and their health implications for children living in outlying urban settlements in Salvador, Bahia State, Brazil. Cad Saude Publica 23: S643-S649.

58. Morales-Espinoza EM, Sanchez-Perez HJ, Garcia-Gil Mdel M, Vargas-Morales G, Mendez-Sanchez JD, et al. (2003) Intestinal parasites in children, in highly deprived areas in the border region of Chiapas, Mexico. Salud Publica Mex 45: 379-388.

59. Narain K, Rajguru SK, Mahanta J (2000) Prevalence of *Trichuris trichiura* in relation to socio-economic & behavioural determinants of exposure to infection in rural Assam. Indian J Med Res 112: 140-146.

60. Nasr NA, Al-Mekhlafi HM, Ahmed A, Roslan MA, Bulgiba A (2013) Towards an effective control programme of soil-transmitted helminth infections among Orang Asli in rural Malaysia. Part 1: prevalence and associated key factors. Parasit Vectors 6: 27.

61. Nguyen PH, Nguyen KC, Nguyen TD, Le MB, Bern C, et al. (2006) Intestinal helminth infections among reproductive age women in Vietnam: prevalence, co-infection and risk factors. Southeast Asian J Trop Med Public Health 37: 865-874.

62. Nishiura H, Imai H, Nakao H, Tsukino H, Changazi MA, et al. (2002) *Ascaris lumbricoides* among children in rural communities in the Northern Area, Pakistan: prevalence, intensity, and associated socio-cultural and behavioral risk factors. Acta Trop 83: 223-231.

63. Norhayati M, Oothuman P, Fatmah MS (1999) Some risk factors of Ascaris and Trichuris infection in Malaysian aborigine (Orang Asli) children. Med J Malaysia 54: 96-101.

64. Nwaneri DU, Ibadin MO, Ofovwe GE, Sadoh AE (2012) Intestinal helminthiasis in children with chronic neurological disorders in Benin City, Nigeria: intensity and behavioral risk factors. World J Pediatr 10.1007/s12519-012-0394-9.

65. Olsen A, Samuelsen H, Onyango-Ouma W (2001) A study of risk factors for intestinal helminth infections using epidemiological and anthropological approaches. J Biosoc Sci 33: 569-584.

66. Ortiz Valencia LI, Drumond Fortes BDPM, De Andrade Medronho R (2005) Spatial Ascariasis risk estimation using socioeconomic variables. International Journal of Environmental Health Research 15: 411-424.

67. Parajuli RP, Umezaki M, Watanabe C (2009) Behavioral and nutritional factors and geohelminth infection among two ethnic groups in the Terai region, Nepal. Am J Hum Biol 21: 98-104.

68. Pham-Duc P, Nguyen-Viet H, Hattendorf J, Zinsstag J, Phung-Dac C, et al. (2013) *Ascaris lumbricoides* and *Trichuris trichiura* infections associated with wastewater and human excreta use in agriculture in Vietnam. Parasitol Int 62: 172-180.

69. Phiri K, Whitty CJ, Graham SM, Ssembatya-Lule G (2000) Urban/rural differences in prevalence and risk factors for intestinal helminth infection in southern Malawi. Ann Trop Med Parasitol 94: 381-387.

70. Quintero K, Duran C, Duri D, Medina F, Garcia J, et al. (2012) Household social determinants of ascariasis and trichuriasis in North Central Venezuela. International Health 4: 103-110.

71. Riess H, Clowes P, Kroidl I, Kowuor DO, Nsojo A, et al. (2013) Hookworm infection and environmental factors in mbeya region, Tanzania: a cross-sectional, population-based study. PLoS Negl Trop Dis 7: e2408. doi: 2410.1371/journal.pntd.0002408.

72. Rísquez P A, Márquez T MD, Quintero P GdC, Ramírez D JP, Requena JG, et al. (2010) Condiciones higiénico-sanitarias como factores de riesgo para las parasitosis intestinales en una comunidad rural venezolana. Rev Fac Med (Caracas) 33: 151-158.

73. Roy E, Hasan KZ, Haque R, Fazlul Haque AKM, Siddique AK, et al. (2011) Patterns and risk factors for helminthiasis in rural children aged under 2 in Bangladesh. SAJCH South African Journal of Child Health 5: 78-84.

74. Saathoff E, Olsen A, Kvalsvig JD, Geissler WP (2002) Geophagy and its association with geohelminth infection in rural schoolchildren from northern KwaZulu-Natal, South Africa. Trans R Soc Trop Med Hyg 96: 485-490.

75. Schmidlin T, Hurlimann E, Silue KD, Yapi RB, Houngbedji C, et al. (2013) Effects of hygiene and defecation behavior on helminths and intestinal protozoa infections in Taabo, Cote d'Ivoire. PLoS One 8: e65722. doi: 65710.61371/journal.pone.0065722. Print 0062013.

76. Scolari C, Torti C, Beltrame A, Matteelli A, Castelli F, et al. (2000) Prevalence and distribution of soil-transmitted helminth (STH) infections in urban and indigenous schoolchildren in Ortigueira, State of Parana, Brasil: implications for control. Rev Inst Med Trop Sao Paulo 42: 115-117.

77. Sherkhonov T, Yap P, Mammadov S, Sayfuddin K, Martinez P, et al. (2013) National intestinal helminth survey among schoolchildren in Tajikistan: Prevalences, risk factors and perceptions. Acta Trop 126: 93-98.

78. Soares Magalhaes RJ, Barnett AG, Clements ACA (2011) Geographical analysis of the role of water supply and sanitation in the risk of helminth infections of children in West Africa. Proc Natl Acad Sci U S A 108: 20084-20089.

79. Steenhard NR, Ornbjerg N, Molbak K (2009) Concurrent infections and socioeconomic determinants of geohelminth infection: a community study of schoolchildren in periurban Guinea-Bissau. Trans R Soc Trop Med Hyg 103: 839-845.

80. Steinmann P, Usubalieva J, Imanalieva C, Minbaeva G, Stefiuk K, et al. (2010) Rapid appraisal of human intestinal helminth infections among schoolchildren in Osh oblast, Kyrgyzstan. Acta Trop 116: 178-184.

81. Stothard JR, Imison E, French MD, Sousa-Figueiredo JC, Khamis IS, et al. (2008) Soil-transmitted helminthiasis among mothers and their pre-school children on Unguja Island, Zanzibar with emphasis upon ascariasis. Parasitology 135: 1447-1455.

82. Teixeira JC, Heller L (2004) Fatores ambientais associados às helmintoses intestinais em áreas de assentamento subnormal, Juiz de Fora, MG. Eng sanit ambient 9: 301-305.

83. Trang DT, van der Hoek W, Cam PD, Vinh KT, Hoa NV, et al. (2006) Low risk for helminth infection in wastewater-fed rice cultivation in Vietnam. East Mediterr Health J 12: 137-143.

84. Do TT, Molbak K, Phung DC, Dalsgaard A (2007) Helminth infections among people using wastewater and human excreta in peri-urban agriculture and aquaculture in Hanoi, Vietnam. Trop Med Int Health 12 Suppl 2: 82-90.

85. Traub RJ, Robertson ID, Irwin P, Mencke N, Andrew Thompson RC (2004) The prevalence, intensities and risk factors associated with geohelminth infection in tea-growing communities of Assam, India. Trop Med Int Health 9: 688-701.

86. Ugbomoiko US, Dalumo V, Ofoezie IE, Obiezue RNN (2009) Socio-environmental factors and ascariasis infection among school-aged children in Ilobu, Osun State, Nigeria. Trans R Soc Trop Med Hyg 103: 223-228.

87. Walker M, Hall A, Basanez MG (2011) Individual predisposition, household clustering and risk factors for human infection with *Ascaris lumbricoides*: new epidemiological insights. PLoS Negl Trop Dis 5: e1047.

88. Wang X, Zhang L, Luo R, Wang G, Chen Y, et al. (2012) Soil-transmitted helminth infections and correlated risk factors in preschool and school-aged children in rural southwest China. PLoS ONE 7: e45939.

89. Wordemann M, Polman K, Menocal Heredia LT, Diaz RJ, Madurga AM, et al. (2006) Prevalence and risk factors of intestinal parasites in Cuban children. Trop Med Int Health 11: 1813-1820.

90. Worrell C, Davis S, Wiegand R, Lopez G, Odero K, et al. Water, Sanitation, and Hygiene-Related Risk Factors for Soil-Transmitted Helminth Infection in Urban School- and Pre-School-Aged Children in Kibera, Nairobi 2013; Atlanta, GA.

91. Xu LQ, Xiao DH, Zhou CH, Zhang XQ, Lan SG, et al. (2001) [On cleanliness of hands in diminution of *Ascaris lumbricoides* infection in children]. Chinese journal of parasitology & parasitic diseases 19: 294-297.

92. Yajima A, Jouquet P, Do TD, Dang TC, Tran CD, et al. (2009) High latrine coverage is not reducing the prevalence of soil-transmitted helminthiasis in Hoa Binh province, Vietnam. Parasitol Res 104: 321-328. doi: 310.1007/s00436-00008-01195-x. Epub 02008 Sep 00425.

93. Yori PP, Kosek M, Gilman RH, Cordova J, Bern C, et al. (2005) Seroepidemiology of strongyloidiasis in the Peruvian Amazon. Cad Saude Publica 21: 1778-1784. Epub 2006 Jan 1779.

94. Young SL, Dave G, Farag TH, Said MA, Khatib MR, et al. (2007) Geophagia is not associated with *Trichuris* or hookworm transmission in Zanzibar, Tanzania. Trans R Soc Trop Med Hyg 101: 766-772.
