# Supplementary material for: Water, Sanitation, Hygiene, and Soil-Transmitted Helminth Infection: A Systematic Review and Meta-Analysis
Source: PLoS Med. 2014 Mar 25;11(3):e1001620. doi: 10.1371/journal.pmed.1001620 (PMC3965411; doi:10.1371/journal.pmed.1001620)
Supplement: Text S2 — MOOSE checklist. (DOC) [file pmed.1001620.s018.doc]

# Text S2: MOOSE Checklist

From: [Donna F. Stroup](http://jama.ama-assn.org/search?author1=Donna+F.+Stroup&sortspec=date&submit=Submit), PhD, MSc; [Jesse A. Berlin](http://jama.ama-assn.org/search?author1=Jesse+A.+Berlin&sortspec=date&submit=Submit), ScD; [Sally C. Morton](http://jama.ama-assn.org/search?author1=Sally+C.+Morton&sortspec=date&submit=Submit), PhD; [Ingram Olkin](http://jama.ama-assn.org/search?author1=Ingram+Olkin&sortspec=date&submit=Submit), PhD; [G. David Williamson](http://jama.ama-assn.org/search?author1=G.+David+Williamson&sortspec=date&submit=Submit), PhD; [Drummond Rennie](http://jama.ama-assn.org/search?author1=Drummond+Rennie&sortspec=date&submit=Submit), MD; [David Moher](http://jama.ama-assn.org/search?author1=David+Moher&sortspec=date&submit=Submit), MSc; [Betsy J. Becker](http://jama.ama-assn.org/search?author1=Betsy+J.+Becker&sortspec=date&submit=Submit), PhD; [Theresa Ann Sipe](http://jama.ama-assn.org/search?author1=Theresa+Ann+Sipe&sortspec=date&submit=Submit), PhD; [Stephen B. Thacker](http://jama.ama-assn.org/search?author1=Stephen+B.+Thacker&sortspec=date&submit=Submit), MD, MSc; for the Meta-analysis Of Observational Studies in Epidemiology (MOOSE) Group. **Meta-analysis of Observational Studies in Epidemiology. A Proposal for Reporting** JAMA. 2000;283(15):2008-2012. doi: 10.1001/jama.283.15.2008

|  | Reported | Comments |
| --- | --- | --- |
| **Reporting of background should include** | | |
| Problem definition | Introduction, paragraphs 4-5 |  |
| Hypothesis statement | Introduction, paragraphs 4-5 |  |
| Description of study outcomes | Introduction, paragraph 5 | Infection with STH species |
| Type of exposure or intervention used | Introduction, paragraphs 4-5 |  |
| Type of study designs used | Methods, paragraphs 2-4 |  |
| Study population | Methods, paragraphs 4-5 | Reported alongside results where appropriate |
| **Reporting of search strategy should include** | | |
| Qualifications of searchers (eg librarians and investigators) | Methods, paragraphs 2-3 |  |
| Search strategy, including time period used in the synthesis and key words | Methods, paragraphs 1-2 |  |
| Effort to include all available studies, including contact with authors | Methods, paragraphs 1-4 |  |
| Databases and registries searched | Methods, paragraphs 1-2 | Pubmed, Web of Knowledge, Embase, LILACS |
| Search software used, name and version, including special features used (eg explosion) | Methods, paragraphs 1-2 | Explosion used, EndNote primary reference management software |
| Use of hand searching (eg reference lists of obtained articles) | Methods, paragraph 1 |  |
| List of citations located and those excluded, including justification | Tables 3-5, Table S1 |  |
| Method of addressing articles published in languages other than English | Methods, paragraph 3 |  |
| Method of handling abstracts and unpublished studies | Methods, paragraph 1-2 |  |
| Description of any contact with authors | Methods, paragraph 4 |  |
| **Reporting of methods should include** | | |
| Description of relevance or appropriateness of studies assembled for assessing the hypothesis to be tested | Methods, paragraph 4 | Broad review focus with all study populations being eligible for inclusion |
| Rationale for the selection and coding of data (eg sound clinical principles or convenience) | Methods, paragraph 4-6 |  |
| Documentation of how data were classified and coded (eg multiple raters, blinding and interrater reliability) | Methods, paragraph 1 |  |
| Assessment of confounding (eg comparability of cases and controls in studies where appropriate) | Methods, paragraph 3 | Noted adjustment variables in spreadsheet |
| Assessment of study quality, including blinding of quality assessors, stratification or regression on possible predictors of study results | Methods, paragraph 9-14 |  |
| Assessment of heterogeneity | Methods, paragraph 10 | Moran’s *I*^2^ ; Cochran’s *Q*-test |
| Description of statistical methods (eg complete description of fixed or random effects models, justification of whether the chosen models account for predictors of study results, dose-response models, or cumulative meta-analysis) in sufficient detail to be replicated | Methods, paragraph 6-7 | DerSimonian and Laird method using Stata 12, natural log of odds ratios was dependent variable |
| Provision of appropriate tables and graphics | Tables, figures | Multiple figures & tables provided through results |
| **Reporting of results should include** | | |
| Graphic summarizing individual study estimates and overall estimate | Table 8, Figures 3-16 | Provided in forest plots, summary results table |
| Table giving descriptive information for each study included | Tables 3-5 |  |
| Results of sensitivity testing (eg subgroup analysis) | N/A |  |
| Indication of statistical uncertainty of findings | Table 8 | Confidence intervals listed throughout results |
| **Reporting of discussion should include** | | |
| Quantitative assessment of bias (e.g., publication bias) | Table 9 |  |
| Justification for exclusion (eg exclusion of non-English language citations) | Table S1 |  |
| Assessment of quality of included studies | Table S2, Table 9 |  |
| **Reporting of conclusions should include** | | |
| Consideration of alternative explanations for observed results | Discussion, paragraph 8 | Discussion of heterogeneity and confounding |
| Generalization of the conclusions (eg appropriate for the data presented and within the domain of the literature review) | Discussion, paragraphs 1, Conclusion paragraph 1 |  |
| Guidelines for future research | Discussion, paragraphs 3-5 |  |
| Disclosure of funding source | Competing Interests |  |
