# Supplementary material for: Water, Sanitation, Hygiene, and Soil-Transmitted Helminth Infection: A Systematic Review and Meta-Analysis
Source: PLoS Med. 2014 Mar 25;11(3):e1001620. doi: 10.1371/journal.pmed.1001620 (PMC3965411; doi:10.1371/journal.pmed.1001620)
Supplement: Text S3 — Original methods protocol. (DOC) [file pmed.1001620.s019.doc]

# Text S3: Methods Protocol Proposal

**Objective**

To examine the effect of water, sanitation, and hygiene (WASH) on soil-transmitted helminth infection (*Ascaris lumbricoides*, *Trichuris trichiura*, hookworm, and *Strongyloides stercoralis*).

**Authors**

This study will be conducted primarily by Eric Strunz (MPH) with support from Meredith Stocks (MPH) and leadership from David Addiss (MPH / MD), Matthew Freeman (MPH / PhD), and Jürg Utzinger (MsC / PhD).

**Reporting**

We will adhere to the PRISMA (Preferred Reporting Items for Systematic Reviews and Meta-Analyses) statement for reporting results. Since we expect primarily observational studies, we will also adhere to the MOOSE (Meta-analysis of Observational Studies in Epidemiology) guidelines.

**Search**

We will systematically search Medline (Pubmed), Embase, and Web of Science for relevant articles. We will also index relevant studies from the bibliography of Ziegelbauer et al. . Additionally, we will search gray literature from the Centers for Disease Control and Prevention (CDC), the Carter Center, the Task Force for Global Health, and the authors’ personal collections. We will not restrict results based on language or publication date. Our search will be performed until March 27, 2013.

Our database search will include two clusters of terms, one for STH and one for WASH. We will search for results that contain at least one term from both clusters.

*STH-related terms*:

helminth, soil-transmitted helminth, geohelminth, ascaris, lumbricoides, trichuris, trichiura, hookworm, ancylostoma, duodenale, necator, americanus, strongyloid*, stercoralis

*WASH-related terms:*

sanitation, sanitary engineering, water supply, waste management, environment*, excre*, faec*, fecal, feces, hand washing, handwashing, hygiene, latrine*, toilet*, water, soap

Note: An asterisk (*) denotes a wildcard character.

**Eligibility**

Article eligibility will be determined by three key criteria. Studies must contain:

1. A distinctly identifiable measurement of WASH variables
2. A direct measurement of STH (e.g. prevalence, intensity)
3. A distinct parameterization of WASH on STH (e.g., odds ratio)

For studies that pool multiple intestinal parasites into one outcome measure (e.g. *Giardia* and STH), we will contact original authors to request disaggregated data. If information about STH outcomes alone is not available, the study will be excluded. Studies that combine multiple WASH factors into one exposure will be included.

Two reviewers (ES, MS) will independently scrutinize the list of titles, journals, and abstracts to determine relevance of the article. Final selection will be based on the full text of all potentially applicable articles by the two reviewers independently. In cases of disagreement, a third reviewer (MF) will examine such articles. Results will be discussed until reaching consensus among all three reviewers.

**Data Collection**

Relevant data from all eligible studies will be collected by one reviewer (ES) into a designated spreadsheet. These data will be independently cross-checked by a second assessor (MS). Extracted data will include:

- Study description (e.g., study design, setting, year)
- Sample size
- The primary research question
- Details on the study population (e.g., age groups)
- The selection process (e.g., random selection)
- WASH factors measured (e.g. water access, latrine use)
- Diagnostic assay, including information about quality control
- Which STH species were measured

The reported odds ratios (ORs) will serve as effect measures. We will collect both crude and adjusted estimates where available. For adjusted ORs, we will attempt to determine what covariates have been controlled for. If not disclosed, we will contact authors to acquire relevant information. We will not exclude studies due to imperfect or nonexistent confounding assessment, though properly adjusted estimates will receive more favorable considerations during our risk of bias assessment. If ORs are reported, but insufficient information about the standard error is provided, authors will be contacted for additional information.

**Assessing Publication / Reporting Bias**

Detecting publication bias is difficult when dealing with dichotomous outcomes, especially when there is significant between-study heterogeneity. In such circumstances, the popular Egger’s test is usually inappropriate, with the potential to result in many false positives. We will primarily assess publication bias through a qualitative funnel plot analysis. We will also enlist the aid of a statistician to pursue an appropriate statistical testing strategy, potentially including those outlined by Harbord et al. , Peters et al. , or Rücker et al. .

**Assessing Internal Bias**

Assessing the risk of bias within studies represents an especially important task for reviews of observational studies. We will draw upon multiple strategies to best gauge the internal validity of eligible articles and our overall body of evidence. We will use a GRADE-inspired approach that considers key sources of potential bias in observational studies. Because GRADE is not primarily intended for observational studies (e.g. allocation concealment does not apply to cross-sectional surveys), we will form our key bias categories from the literature (especially Ziegelbauer et al., 2012) and two instruments highlighted by the Cochrane Collaboration: the Downs and Black Tool and the Newcastle - Ottawa Scale.

We will focus on five core potential sources of bias in our assessment: diagnostics, exposure assessment, confounding assessment, response rate, and selective reporting.

- **Diagnostics** – Is a diagnostic assay clearly mentioned? Is there any form of quality control in the diagnostic process (e.g. a senior technician doing spot-checks)?
- **Exposure Assessment** – Was exposure assessment (e.g. access to clean water, washing hands) ascertained via a self-reported survey response (unreliable) or observed directly by investigators (more reliable)? Is there any attempt to gauge proper use of water / hygiene or some form of “quality control” for the exposures?
- **Confounding Assessment** – Are only crude estimates computed? Or has matching and / or multiple logistic regression been undertaken to control for important potential confounders?
- **Response Rate** – is the response rate (or loss-to-follow-up) similar for infected vs. non-infected individuals?
- **Selective Reporting –** is there evidence of selective reporting within an article (e.g. outlining certain variables of interest in the methods but not providing any data on them in the results)?

Each study will receive one of three rankings for each source of bias: low, unclear, or high. This judgment will be independently assessed by two reviewers (ES, MS) with any disagreements arbitrated by a senior assessor (MF or DA). We will populate a table of these assessments for all studies when considering the overall body of evidence. When determining the risk of bias for the total body of evidence, we will use similar categories (low, unclear, or high) based on the cumulative table and group consensus. This determination will contribute to the overall GRADE score for the body of evidence (i.e. downgrading or upgrading the observational studies).

To further help us determine the overall risk of bias and quality of evidence, we will use a simple score system to inform our decision-making. This will also be independently assessed by two reviewers and arbitrated by a senior assessor. Studies will receive points based on characteristics listed in Table 1 (below):

| **Table 1: Measures & Descriptions for Bias Assessment Score** | **Points** |
| --- | --- |
| *Diagnostics* | |
| Rigorous diagnostic approach (e.g., Kato-Katz, FLOTAC) | +1 |
| Diagnosis from multiple stools, techniques and/or explicit quality control (at least 5% stools reviewed by a senior lab technician) | +1 |
| No mention of diagnostic assay | 0 |
| *Exposure Assessment* | |
| Exposure was observed directly by the research team | +1 |
| Exposure was assessed using a questionnaire | 0 |
| *Study Design* | |
| Observational study | 0 |
| Quasi-experimental design | +1 |
| Experimental design (RCT or CRT = inclusion of randomized control ) | +2 |
| Purposively calculated sample size to address STH as a WASH outcome | +1 |
| *Other Strengths and Limitations* | |
| Did not control for confounding variables | -1 |
| Evidence of selective reporting within the study | -1 |

**Analysis Plan**

We will consider pursuing a meta-analysis for appropriate sub-groupings of studies if the opportunity and sufficient evidence arises. We expect significant heterogeneity due to the nature of non-randomized studies and the different variables involved in this field (e.g. different worms, different types of WASH features). However, we will additionally use Moran’s *I^2^* and Cochrane’s *Q*-tests to determine the heterogeneity between studies.

Due to the expected heterogeneity, it is possible that no meta-analysis will be conducted. However, if analysis is deemed appropriate, SAS and RevMan will be utilized for a random-effects meta-analysis using the DerSimonian and Laird method . Our dependent variable will be the natural log of the reported odds ratios. We will weight studies based on their standard errors, and our primary analysis will consider odds ratios that have been adjusted for confounders.

If sufficient numbers of studies are acquired, we will also consider subgroup analyses and / or meta-regression strategies. We have pre-specified type of worm, WASH sector, population of interest (e.g. school-age children vs. adults), and geographical region as important possible contributors to heterogeneity. It is likely that any meta-analysis will require multiple analyses that contribute to an overall assessment of the data. This could include calculating an aggregate estimate that combines all WASH sectors—in spite of heterogeneity—in order to determine if WASH, with all its variability, contributes to any positive average effect. Such insight could prove valuable for policymakers who are considering the WASH strategy in general. Additional meta-analyses could be conducted for each possible sub-group (e.g. different worm types) to better examine the relationship between WASH and STH. We recognize that exhaustive sub-group analysis can result in data dredging and will take care to restrict our attention to our pre-specified sources of heterogeneity.

References

1. Ziegelbauer K, Speich B, Mäusezahl D, Bos R, Keiser J, et al. (2012) Effect of sanitation on soil-transmitted helminth infection: systematic review and meta-analysis. PLoS Med 9.

2. Harbord RM, Egger M, Sterne JA (2006) A modified test for small-study effects in meta-analyses of controlled trials with binary endpoints. Stat Med 25: 3443-3457.

3. Peters JL, Sutton AJ, Jones DR, Abrams KR, Rushton L (2006) Comparison of two methods to detect publication bias in meta-analysis. JAMA 295: 676-680.

4. Rücker G, Schwarzer G, Carpenter J (2008) Arcsine test for publication bias in meta-analyses with binary outcomes. Stat Med 27: 746-763.

5. DerSimonian R, Laird N (1986) Meta-analysis in clinical trials. Control Clin Trials 7: 177-188.
